# Synthesis of sp^3^-rich heterocyclic frameworks by a divergent synthesis strategy†

**DOI:** 10.1039/d3ob00351e

**Published:** 2023-05-16

**Authors:** Kim T. Mortensen, Denedy S. Y. Wong, Thomas A. King, Hannah F. Sore, David R. Spring

**Affiliations:** a Department of Chemistry, University of Cambridge Lensfield Rd Cambridge UK spring@ch.cam.ac.uk

## Abstract

Fragment-based lead discovery (FBLD) often relies on flat, aromatic compounds which display undesirable physicochemical properties with limited exit vectors for fragment growth. Herein, we report concise synthetic strategies to sp^3^-rich heterocyclic fragments encompassing polar exit vectors poised for fragment-to-lead (F2L) development.

## Abstract 2

Herein, we present sp^3^-rich fragments, that display optimal physicochemical properties and exit vectors ideal for fragment-based lead discovery.

## Graphical abstract

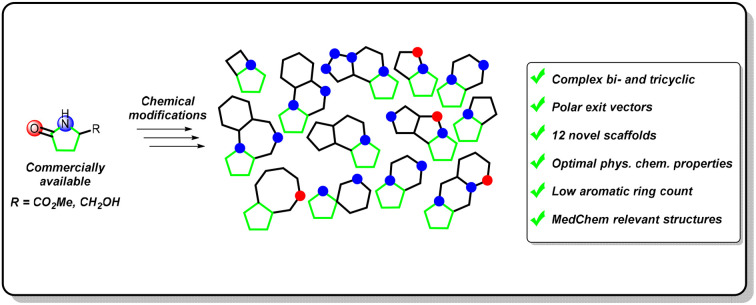

FBLD is a well-established and mature platform that provides hits against various biological targets and to this date has provided six FDA-approved drugs, including against a previously “undruggable” target.^1^ FBLD uses fewer compounds to sample a larger part of chemical space.^2,3^ The broader coverage can in theory afford hits against a variety of different biological targets. This was recently shown by Kidd *et al.*^4^ where a fragment library of 40 compounds delivered structurally diverse hits against different biological targets. Generally, high-throughput screening (HTS) affords potent, molecular obese hits, whereas FBLD affords weak, but efficient binders as more atoms are involved in the binding interactions.^5^ The low molecular weight of the fragments allows for a precise control of the physicochemical properties. This is crucial, as 90% of compounds in development are poorly soluble.^6^

Several issues have been identified regarding the commercially available fragment libraries. These libraries (a) rely heavily on “flat” (sp^2^-rich) aromatic compounds which impairs progression through the drug discovery process, (b) contain fragments which cannot be explored in multiple directions using current synthetic methods, (c) have few or no polar exit vectors built into the fragments.^7,8^

In the development of our fragment library, we especially wanted to address issues a and c above. Decreasing the number of aromatic rings and increasing the degree of saturation (fraction of sp^3^-hybrised carbons) has a direct impact on drug development.^9^ There is strong demand to devise fragments with a variety of synthetic handles branching out around that fragment to pick up binding interactions.^10^ There is an ongoing debate to whether increased complexity leads to decreased probability of ligand–protein interactions^11^ as it will be harder to find a complementary match with the protein.^12^ Therefore, synthesis of saturated polycyclic scaffolds with polar exit handles afford an ideal compromise between rigidity, complexity and diversity. We quickly realised that pyrrolidines and pyrrolidones meet these requirements for our fragment library. These scaffolds have appeared in FBLD campaigns,^13^ however it would be beneficial to further explore diverse scaffolds from this privileged building block as it is present in natural products and components of approved drugs (Fig. 1).

**Fig. 1:**
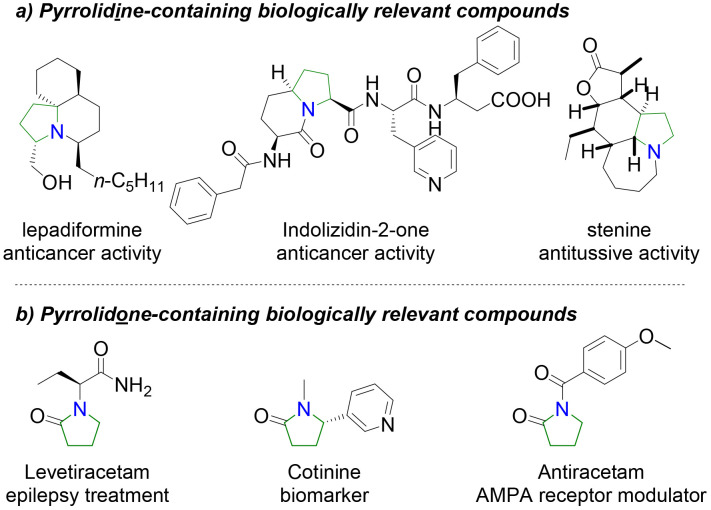
(a) Biologically relevant pyrrolidine- and (b) pyrrolidone-containing compounds.

We settled on the two cheap, commercially available building blocks 1 and 2 due to their high content of sp^3^ and synthetic handles (Scheme 1). The reactive nitrogen was first modulated to introduce an additional functional handle that was linked up with the adjacent carbonyl group. An aldehyde–amide–dienophile (AAD) multicomponent reaction^14,15^ provided complex, sp^3^-enriched [5,7,6]- and [5,6,6]-tricyclic fragments (6a, 6b, 7) (Scheme 1). This introduced an amide and an alkene for further vector exploration. Another [5,6,6]-tricyclic fragment (8) was accomplished by an intramolecular [4 + 2] cycloaddition between acylnitroso^16^ and diene in an inseparable 1 : 2 diastereomeric mixture.^17^

**Scheme 1:**
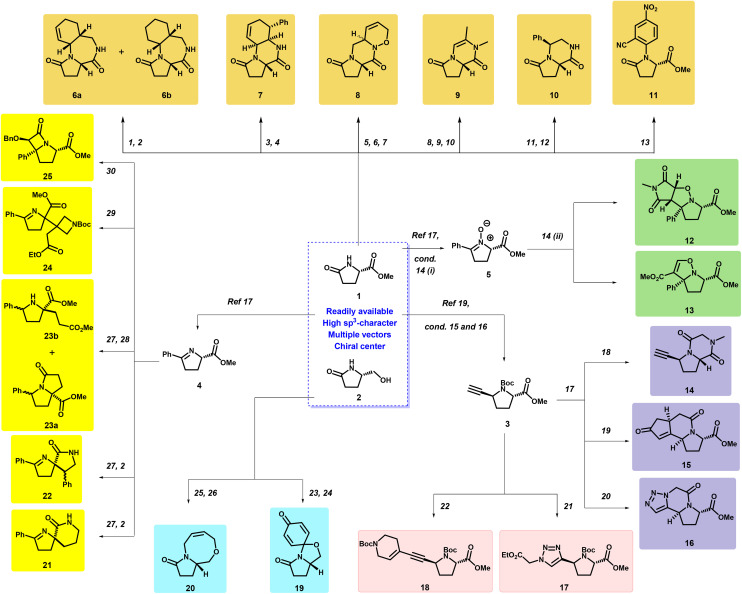
Reaction conditions: (1) *trans*-crotonaldehyde, acrylonitrile, *p*TSA·H_2_O, PhMe, MW, 44%; (2) RANEY®-nickel, MeOH; for 6a 30% + 6b 19%, 21 66%, 22 55%; (3) *trans*-β-nitrostyrene, *trans*-crotonaldehyde, *p*TSA·H_2_O, PhMe, MW, 55%; (4) (i) Zn, 6 M HCl (aq.), MeOH, (ii) PhMe, 54% over two steps; (5) ((*E*)-5-bromopenta-1,3-diene, NaH, DMF, 52%; (6) NH_2_OH·HCl, KOH, MeOH, 48%; (7) *n*Bu_4_NIO_4_, H_2_O/MeOH (5 : 1), 36%; (8) propargyl bromide, NaH, DMF, 67%; (9) MeNH_2_, 80%; (10) (i) K_2_CO_3_, KOH, TBAB, THF, MW, (ii) *p*TSA, MeOH, 46%, over two steps; (11) *trans*-β-nitrostyrene, *n*BuLi, S4-*anti* (18%) and S4-*syn* (29%); (12) Zn, 6 M HCl (aq.), MeOH, 44%; (13) 2-fluoro-5-nitrobenzonitrile, NaH, DMF, 32%; (14) (i) CH_3_ReO_3_, UHP, MeOH, (ii) for 12: *N*-methylmaleimide, CH_2_Cl_2_, 10% over two steps; for 13: methyl propiolate, CH_2_Cl_2_, 53% over two steps; (15) CuBr·Me_2_S, TMSCCMgBr, BF_3_·OEt_2_, Et_2_O, 88%; (16) K_2_CO_3_, MeOH, 97%; (17) (i) TFA, CH_2_Cl_2_; (ii) RCOOH, oxyma, EDC·HCl, CH_2_Cl_2_; for 14: *N*-(*tert*-butoxycarbonyl)-*N*-methylglycine, 57%, over two steps; for 15: 3-butenoic acid, 87%, over two steps; for 16: 2-azidoacetic acid; (18) TFA, CH_2_Cl_2_, 65%; (19) Co_2_(CO)_8_, CH_2_Cl_2_ then NMO, 68%; (20) CH_3_Cl, 48% over three steps; (21) ethyl azidoacetate, CuSO_4_·H_2_O, NaOAs, *t*BuOH/H_2_O (1 : 1), 69%; (22) 1-(*tert*-butoxycarbonyl)-1,2,3,6-tetrahydropyridin-4-yl trifluoromethanesulfonate, PdCl_2_(PPh_3_)_2_, CuI, THF, Et_3_N, 76%; (23) 4-iodoanisole, CuI, Cs_2_CO_3_, DMEDA, DMF, 89%; (24) CAN, MeCN/H_2_O (3 : 1), 56%; (25) allyl bromide, NaH, DMF, 36%; (26) Grubbs 2^nd^, CH_2_Cl_2_, 57%; (27) Cu(MeCN)_4_BF_4_, PCy_3_, Et_3_N, THF; 21: acrylonitrile, 72%; 22: *trans*-β-nitrostyrene, 75%; S20: methyl acrylate, 67%; (28) NaBH_3_CN, AcOH, PhMe, 23a 12% and 23b 39%; (29) *tert*-butyl 3-(2-ethoxy-2-oxoethylidene)azetidine-1-carboxylate, DBU, Ag_2_O, PhMe, 73%; (30) benzyloxyacetyl chloride, Et_3_N, CH_2_Cl_2_, 70%.

Next, the focus was turned to bicyclic fragments by the formation of 9 from an allenamide cycloisomerisation reaction between an alkyne and 2° amide. The use of nitro alkenes was revisited as they are readily available and can introduce a large range of synthetic handles and modulate physicochemical properties, here showcased by *trans*-β-nitrostyrene. The nitrogen of 1 was alkylated to afford two separable diastereomers, one of which was subsequently cyclised to amide 10. The stereochemistry was confirmed by NOESY. Lastly, 1 was treated with 2-fluoro-5-nitrobenzo-nitrile to afford 11.

To further explore the toolbox of enabling chemistries, the inherent amide of 1 was transformed into a nitroso (5), which was successfully treated with *N*-methyl maleimide and methyl propiolate to provide the [5,5,5]- and [5,5]-fused fragments 12 and 13, respectively. We were successful in obtaining a crystal structure of 12 to confirm its absolute stereochemistry. Next, an alkyne-handle was introduced on **1** to further expand the fragment library. The alkyne group was introduced *via* Cu(i)-mediated chemistry to selectively afford the (2*S*,5*S*)-isomer followed by TMS-deprotection (3).^19,20^ Firstly, the free amine (3) was acylated with *N*-methyl glycine followed by cyclisation of the *N*-Me to the ester moiety of 3 to afford 14. Next, the amine of 3 was acylated by alkene- and azide-containing carboxylic acids and then cyclised to the alkyne under Pauson–Khand and azide–alkyne cycloaddition conditions to afford scaffolds 15 and 16, respectively. The absolutely stereochemistry of 15 was confirmed by NOESY. Lastly, to probe the alkyne as a synthetic handle for vector exploration and as a means to introduce additional synthetic handles, treating it under copper-catalysed azide–alkyne cycloaddition and Sonogashira conditions gave successfully fragments 17 and 18, respectively.

To explore other enabling chemistries and expand, we set out to investigate the hydroxyl functionality of 2. Spirocyclic compounds are of great importance in drug discovery^18^ and to introduce a spirocyclic moiety, 2 was directly treated with iodoanisole under Buchwald conditions^21^ and then treated with CAN to provide 19. This fragment could then be further diversified. 2 could also be double-alkylated by allyl bromide followed by ring-closing metathesis (RCM) conditions to give 20.

With the presence of 1-pyrrolidines in various drugs,^22^ retaining the imine vector could expand the fragment library further. Cu-mediated Michael addition of azomethine ylide 4 to various Michael acceptors was successfully carried out to afford fragments 21–24 and their corresponding intermediates (ESI†) in undefined enantiomeric mixtures.

Integrated nitrogen-containing functional groups were ideal for internal cyclisation to spirocyclic fragments 21 and 22. For methyl acrylate, the imine was partially reduced to afford a separable mixture of the [5,5]-fused system 23a and the non-cyclised amine 23b under these mild conditions. For substituted methyl acrylate, equimolar Ag_2_O was necessary to obtain 24. Lastly, the imine vector was explored to afford [5,4]-fused fragment 25*via* ketene formation. The absolutely stereochemistry of 25 was verified by NOESY.

We have highlighted the development of a fragment library that evolves around readily accessible starting materials to generate diverse and complex heterocyclic scaffolds in a low step count. The physicochemical properties were strictly controlled during this study to generate sp^3^-rich scaffolds that display a variety of different polar exit vectors and low aromatic count. These fragments display optimal physicochemical properties and unique structural features for a F2L campaign. A total of 22 fragments, of which 12 are novel, were synthesised from commercially available starting materials and incorporated important structural moieties applied in drug discovery. A few of the reported fragments include structural alerts (*e.g.* enone or terminal alkyne),^23^ for which attention should be given in a screening campaign as these moieties can be promiscuous binders and display unwanted toxicity. For fragment 14, functionalisation of the terminal alkyne functionality could be envisioned subsequently to extensively increase the fragment library size, however this is beyond the scope of this study.

The physicochemical properties were continuously assessed during this study to ensure compliance to the “rule of three”^24^ and to our vision to generate sp^3^-rich fragments with a variety of different polar exit vectors. The deliberate integration of sp^3^-rich motifs is clearly highlighted in Table 1, with fragments displaying high Fsp^3^ and low aromatic fraction (FAr) values. A deliberate incorporation of stereocentres and polar exit vectors did not compromise the overall strong compliance to the “rule of three”.^24^ To assess the overall shape diversity of the fragment library, we applied principal moments of inertia (PMI) plots (Fig. 2).^25^ This is an effective way to evaluate 3D diversity of the fragment library. Our library was compared to a commercial fragment library from Maybridge. It is clear to see an overall good distribution of shape diversity. This also shows that even though the library was concentrated around fused ring systems, a good distribution can be achieved.

**Fig. 2:**
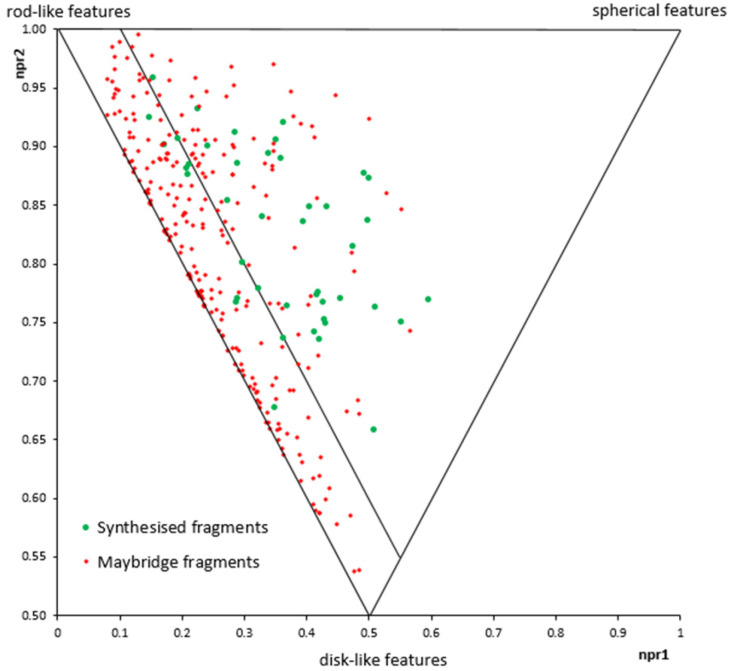
PMI plot of the synthesised fragments compared against a commercially available fragment library from Maybridge.

**Table 1:** Calculated physicochemical properties of Boc-deprotected fragments and compared to commercially available libraries

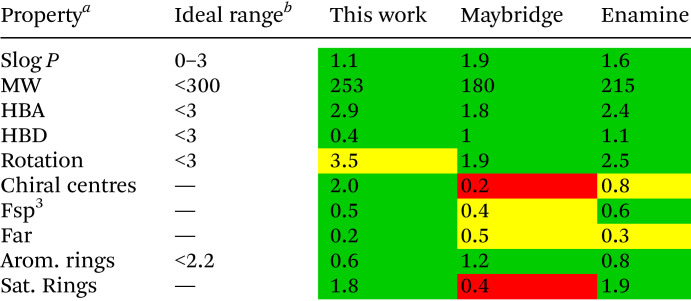

a MW = molecular weight, HBA = number of hydrogen bond acceptors, HBD = number of hydrogen bond donors, Fsp^3^ = number of sp^3^ hybridised carbons/total carbon count, FAr = number of aromatic carbons/total carbon count.

b Guidelines in accordance with “rule of three”.

A final analysis was performed to assess the natural product (NP)-likeness score of the disclosed fragment library (Fig. 3).^26^ In overall, the fragment library displays great distribution among the different NP databases.

**Fig. 3:**
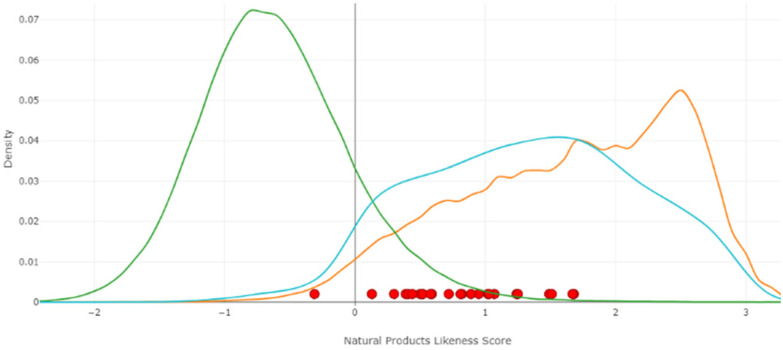
NP-likeness score of reported fragment library plotted against selected synthetic molecules from the ZINC database, a large collection of NPs and a sublibrary of NPs from the ZINC NP database.

To conclude, this communication illustrates our continuous efforts to tackle some of the current issues of fragment libraries and the challenges to advance fragments through drug discovery. There is a shortage of synthetic strategies for sp^3^-rich heterocyclic fragment libraries with a variety of exit vectors around the given scaffold. We have developed such strategies that allow for quick expansion of a given fragment. Each fragment displays improved physicochemical properties that ensures an optimal starting point in the fragment expansion stage. We have had a clear vision to limit the amount of aromatic groups, due to its strong influence on solubility, which again has a crucial negative impact on the success rate in drug discovery programs. We have moreover successfully diversified commercial building blocks to versatile fragments, which also displays great NP-likeness score. Furthermore, with the developed synthetic strategies having a strong focus on integrating medicinal chemistry relevant moieties, this fragment library has a strong potential to provide hits for multiple biological targets in the future. These findings will be able to advance the area of FBLD and support the further development of fragments into approved drugs that can change the life of patients worldwide.

## Conflicts of interest

There are no conflicts to declare.

## Supplementary Material

OB-021-D3OB00351E-s001

OB-021-D3OB00351E-s002
