# Supplementary material for: Synthesis of sp3-rich heterocyclic frameworks by a divergent synthesis strategy
Source: Org Biomol Chem. 2023 May 16;21(22):4591–5. doi: 10.1039/d3ob00351e (PMC10246434; doi:10.1039/d3ob00351e)
Supplement: OB-021-D3OB00351E-s001 [file OB-021-D3OB00351E-s001.pdf]

Electronic Supplementary Material (ESI) for Organic & Biomolecular Chemistry.
This journal is © The Royal Society of Chemistry 2023

Synthesis of sp³-rich Heterocyclic Frameworks by a Divergent Synthesis Strategy

Kim T. Mortensen,[a] Denedy S. Y. Wong,[a] Thomas A. King,[a] Hannah F. Sore[a] and David R. Spring*[a]

[a]Department of Chemistry, University of Cambridge, Lensfield Rd, Cambridge, UK.

*Correspondence: spring@ch.cam.ac.uk

Contents

General Information 2
Synthetic procedures 3
NMR spectra 22
Crystallographic data 68
Computational Analysis 70
References 72

General Information

When reactions required inert conditions, heat gun-dried glassware and inert nitrogen atmosphere were applied. All reagents were used as received from commercial sources or prepared as described in the literature unless otherwise stated. Acetonitrile (MeCN), dichloromethane (CH_2Cl_2), methanol (MeOH) and toluene were distilled from calcium hydride. Tetrahydrofuran (THF) was dried using sodium wire and distilled from a mixture of calcium hydride and lithium aluminium hydride with triphenylmethane as indicator. Diethyl ether (Et_2O) was distilled from a mixture of calcium hydride and lithium aluminium hydride. Ethyl acetate (EtOAc) and petroleum ether (PE) were distilled before use. Anhydrous dimethylformamide (DMF) and 1,2-dichloroethane (DCE) were purchased from commercial sources and used without further purification. Reactions were monitored by thin layer chromatography (TLC) using pre-coated Merck glass backed silica gel 60 F_{254} plates and visualised by quenching of UV fluorescence or by staining with a $KMnO_4$ solution. Flash column chromatography was carried out using Merck 9385 Kieselgel 60 SiO_2 (230-400 mesh). Optical rotations were measured on an Anton Paar MCP 100 Modular Compact Polarimeter. Infrared (IR) spectra were recorded neat on a Perkin-Elmer Spectrum One spectrometer using an ATR sampling accessory either as solids or liquid films. Selected absorptions (ν) are reported in wavenumbers (cm^{-1}). Proton magnetic resonance spectra were recorded using a Bruker Avance III HD (400 MHz; Smart probe), Bruker Avance III (400 MHz; QNP Cryoprobe) or Bruker Avance III (500 MHz, DUL Cryoprobe) spectrometers. Chemical shifts (δ) are quoted in ppm to the nearest 0.01 ppm and are referenced to the residual non-deuterated solvent peak. Coupling constants (*J*) are reported in Hertz, rounded to the nearest 0.1 Hz. Carbon magnetic resonance spectra were recorded using a Bruker Avance III HD (101 MHz), Bruker Avance III (101 MHz) or Bruker Avance 500 (126 MHz) spectrometers with broadband proton decoupling. Chemical shifts (δ) are quoted in ppm to the nearest 1 ppm. High resolution mass spectrometry (HRMS) measurements were recorded on a Micromass Q-TOF, Waters Vion IMS Qtof or a Waters LCT Premier TOF mass spectrometer using electrospray ionisation (ESI) techniques. Mass values are reported within the ±5 ppm error limit.

Synthetic procedures

General procedure A: To a solution of substrate in MeOH under nitrogen atmosphere was added Raney-Nickel (50% in H_2O). A hydrogen atmosphere was established by three vacuum/H_2 cycles before H_2 was bubbled through the solution for 5-10 min and then the mixture was vigorously stirred under an atmosphere of H_2. The reaction mixture was filtered through a pad of celite using EtOAc and MeOH. The filtrate was concentrated under reduced pressure and the crude product was purified by flash column chromatography.

General procedure B[1]: To a stirring solution of amine substrate and carboxylic acid in CH_2Cl_2 were added Oxyma and EDC·HCl. The mixture was stirred overnight. The following day, sat. $NaHCO_3$ was added and the aqueous solution was extracted with CH_2Cl_2 (3 x). The combined organic layers were dried over Na_2SO_4, filtered and concentrated *in vacuo*. The crude product was purified by flash column chromatography.

General procedure C: To a stirring solution of substrate in DMF at 0 °C was added NaH (60% in mineral oil) in one portion and the mixture was stirred at this temperature for 10 min followed by 15 min at rt. The resulting mixture was then down to 0 °C for 10 min. Alkylation reagent was added and the reaction mixture was stirred at rt for the specified time. The reaction mixture was quenched by the addition of brine and extracted with EtOAc (3 x). The combined organic layers were washed with brine (6x), dried over Na_2SO_4, filtered and concentrated *in vacuo*. The crude product was purified by flash column chromatography.

Methyl (*S*)-1-((1*R*,6*S*)-6-cyanocyclohex-2-en-1-yl)-5-oxopyrrolidine-2-carboxylate[2] (S1)

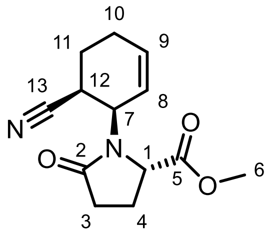

Literature procedure was modified by microwave heating. A microwave vial (2-5 mL) containing methyl (*S*)-(+)-2-pyrrolidone-5-carboxylate (237 µL, 2.03 mmol), *trans*-crotonaldehyde (252 µL, 3.04 mmol, 1.5 equiv.), acrylonitrile (531 µL, 8.10 mmol, 4.0 equiv.), *p*TSA·H_2O (7.7 mg, 0.041 mmol, 2 mol%) in toluene (4.0 mL) was stirred at 180 °C in a microwave irradiation for 40 min. *Two identical reactions were performed and combined*. The combined reaction mixture was concentrated under reduced pressure and directly purified by flash column chromatography (EtOAc:PE_{40-60} 7:13 to 3:1) to afford methyl (*S*)-1-((1*R*,6*R*)-6-cyanocyclohex-2-en-1-yl)-5-oxopyrrolidine-2-carboxylate **S1** (463.2 mg, 44%) as a clear oil. Spectral data is in correspondence with reported literature values.[2] R_f = 0.57 (EtOAc:PE_{40-60} (7:3), $KMnO_4$); IR (neat) ν (cm^{-1}): 2955, 2239, 1740, 1692, 1453, 1403, 1201, 1176; ^{1}H-NMR (400 MHz, $CDCl_3$): δ 6.03-5.97 (1H, m, H-9), 5.44-5.40 (1H, m, H-8), 4.93-4.89 (1H, m, H-7), 4.56-4.54 (1H, m, H-1), 3.77 (3H, s, H-6), 3.47-3.43 (1H, m, H-12), 2.63-2.34 (4H, m, H-10a/H-3a/H-4), 2.16-1.90 (4H, m, H-10b/H-11/H-3b); ^{13}C-NMR (100 MHz, $CDCl_3$): δ 176.2 (C-2), 173.9 (C-5), 132.5 (C-9), 121.8 (C-8), 119.8 (C-13), 58.5 (C-1), 52.8 (C-6), 48.6 (C-7), 30.3 (C-12), 29.5 (C-4), 25.3 (C-3), 23.7 (C-11), 21.7 (C-10); LCMS (ESI): *m/z* calcd for $C_{13}H_{17}N_2O_3$ $[M+H]^+$: 249.1; found: 249.1; $[\alpha]_D^{20}$ = -54.1 (c 0.32, $CHCl_3$).

Hydrogenation of S1

According to the **general procedure A**, methyl (*S*)-1-((1*R*,6*R*)-6-cyanocyclohex-2-en-1-yl)-5-oxopyrroli-dine-2-carboxylate **S1** (50.0 mg, 0.20 mmol) in MeOH (2.0 mL) and Raney-Nickel (2.0 mL, 50% in H_2O) were vigorously stirred for 5 h. The residues were purified by flash column chromatography (CH_2Cl_2:MeOH 99:1 to 9:1) to the fully and partially hydrogenated products, affording (3a*S*,6a*S*,10a*S*)-3,3a,5,6,6a,7,8,10a-octahydro-1*H*-

benzo[*f*]pyrrolo[1,2-*a*][1,4]diazepine-1,4(2*H*)-dione **(6a)** and (3a*S*,6a*S*,10a*S*)-decahydro-1*H*-benzo[*f*]pyrrolo[1,2-*a*][1,4]diazepine-1,4(2*H*)-dione **(6b)**, respectively. *The two products were difficult to separate by flash column chromatography. Besides a few pure fractions, co-eluting fractions were collected affording 10 mg of a 1:1 mixture.*

(3a*S*,6a*R*,10a*R*)-3,3a,5,6,6a,7,8,10a-Octahydro-1*H*-benzo[*f*]pyrrolo[1,2-*a*][1,4]diazepine-1,4(2*H*)-dione (6a)

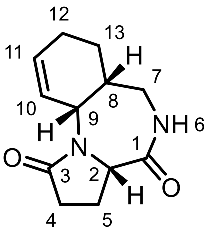

Off-white amorphous solid (13.1 mg, 30%). R_f = 0.21 (CH_2Cl_2:MeOH (9:1), $KMnO_4$); IR (neat) ν (cm^{-1}): 3229, 3082, 2916, 2868, 1668, 1643, 1256; ^{1}H-NMR (400 MHz, $CDCl_3$): δ 6.62 (1H, t, *J* 5.00 Hz, H-6), 6.06-6.01 (1H, m, H-11), 5.85-5.82 (1H, m, H-10), 4.43-4.39 (1H, m, H-2), 3.90-3.86 (1H, m, H-9), 3.30 (1H, ddd, *J* 14.57, 10.86, 4.93 Hz, H-7a), 3.16 (1H, ddd, *J* 14.57, 7.73, 4.93 Hz, H-7b), 2.52-2.40 (3H, m, H-4/H-5a), 2.27-2.11 (3H, m, H-5b/H-8/H-13a), 2.04-1.95 (1H, m, H-13b), 1.68-1.58 (1H, m, H-12a), 1.52-1.46 (1H, m, H-12b); ^{13}C-NMR (100 MHz, $CDCl_3$): δ 175.4 (C-1), 174.3 (C-3), 130.3 (C-11), 123.7 (C-10), 65.3 (C-2), 51.7 (C-9), 45.0 (C-7), 40.3 (C-8), 31.6 (C-4), 24.5 (C-13), 23.59 (C-12), 23.57 (C-5); HRMS (ESI): *m/z* calcd for $C_{12}H_{17}N_2O_2$ $[M+H]^+$: 221.1290; found: 221.1293; $[\alpha]_D^{20}$ = - 229.8 (c 0.5, $CHCl_3$).

(3a*S*,6a*R*,10a*R*)-Decahydro-1*H*-benzo[*f*]pyrrolo[1,2-*a*][1,4]diazepine-1,4(2*H*)-dione (785-01) (6b)

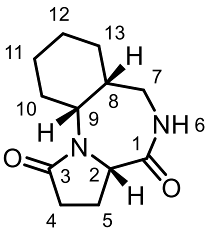

White amorphous solid (8.5 mg, 19%). R_f = 0.26 (CH_2Cl_2:MeOH (9:1), $KMnO_4$); IR (neat) ν (cm^{-1}): 3205, 3090, 2935, 2872, 1702, 1675, 1656, 1418; ^{1}H-NMR (400 MHz, $CDCl_3$): δ 6.19 (1H, br s, H-6), 4.35 (1H, dd, *J* 7.75, 5.62 Hz, H-2), 3.90-3.85 (1H, m, H-9), 3.63-3.57 (1H, m, H-7a), 3.28-3.21 (1H, m, H-7b), 2.59-2.47 (2H, m, H-4a/H-5a), 2.40-2.23 (2H, m, H-8/H-4b), 2.14-2.05 (1H, m, H-5b), 1.96-1.85 (3H, m, H-10/H-11a), 1.57-1.46 (5H, m, H-11b/H-12/H-13); ^{13}C-NMR (100 MHz, $CDCl_3$): δ 175.1 (C-3), 171.9 (C-1), 59.8 (C-2), 55.7 (C-9), 44.5 (C-7), 36.2 (C-8), 31.0 (C-4), 29.8 (C-13), 27.7 (C-10), 24.2 (C-11), 21.7 (C-12), 20.3 (C-5); HRMS (ESI): *m/z* calcd for $C_{12}H_{19}N_2O_2$ $[M+H]^+$: 223.1447; found: 223.1447; $[\alpha]_D^{20}$ = 22.0 (c 0.25, $CHCl_3$).

Methyl (*S*)-1-((1*R*,2*R*,3*S*)-2-nitro-1,2,3,6-tetrahydro-[1,1'-biphenyl]-3-yl)-5-oxopyrrolidine-2-carboxylate (S2)

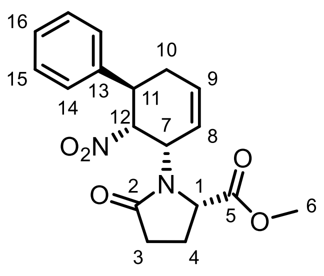

Modified literature procedure by Weber *et al.*[3] A microwave vial (2-5 mL) containing methyl (*S*)-(+)-2-pyrrolidone-5-carboxylate (155 μL, 0.985 mmol), *trans*-crotonaldehyde (245 μL, 2.96 mmol, 3.0 equiv.), *trans*-β-nitrostyrene (293.8 mg, 1.97 mmol, 2.0 equiv.) and *p*TSA·H_2O (3.7 mg, 0.020 mmol, 2 mol%) in toluene (3.2 mL) was stirred at 150 °C under microwave irridation. When full conversion was observed by TLC (2 h), the reaction mixture was concentrated under reduced pressure and directly purified by flash column chromatography (EtOAc:$PE_{40\text{-}60}$ 1:4 to 11:9) to afford methyl (*S*)-1-((1*R*,2*R*,3*S*)-2-nitro-1,2,3,6-tetrahydro-[1,1'-biphenyl]-3-yl)-5-oxopyrrolidine-2-carboxylate **S2** (188.1 mg, 55%) as an orange foam. *Minor impurities were observed by ^{1}H-NMR*. R_f = 0.27 (CH_2Cl_2:MeOH (9:1), $KMnO_4$); IR (neat) ν (cm^{-1}): 2957, 1735, 1698, 1548, 1399, 1202, 1174, 736; ^{1}H-NMR (400 MHz, $CDCl_3$): δ 7.34-7.30 (2H, m, H-15), 7.25-7.22 (3H, m, H-14/H-16), 6.07-6.03 (1H, m, H-8), 5.63-5.59 (2H, m, H-7/H-9), 5.22 (1H, dd, *J* 12.57, 5.43 Hz, H-12), 4.60-4.58 (1H, m, H-1), 3.75 (3H, s, H-6), 3.60-3.52 (1H, m, H-11), 2.68-2.60 (1H, m, H-10a), 2.56-2.51 (1H, m, H-3a), 2.38-2.30 (3H, m, H-3b/H-4a/H-10b), 2.05-1.99 (1H, m, H-4b); ^{13}C-NMR (100

MHz, $CDCl_3$): δ 175.9 (C-2), 173.6 (C-5), 139.9 (C-13), 131.9 (C-8), 129.2 (C-15), 128.0 (C-16), 127.3 (C-14), 122.0 (C-9), 89.2 (C-12), 59.5 (C-1), 52.6 (C-6), 47.1 (C-7), 40.5 (C-11), 33.3 (C-10), 28.9 (C-3), 24.4 (C-4); HRMS (ESI): *m/z* calcd. for $C_{18}H_{21}N_2O_5$ $[M+H]^+$: 345.1450; found: 345.1435.

Methyl (*S*)-1-((1*R*,2*R*,3*S*)-2-amino-1,2,3,6-tetrahydro-[1,1'-biphenyl]-3-yl)-5-oxopyrrolidine-2-carboxylate (S3)

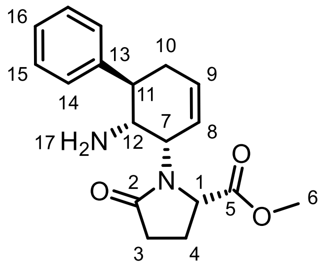

To a solution of methyl (*S*)-1-((1*R*,2*R*,3*S*)-2-nitro-1,2,3,6-tetrahydro-[1,1'-biphenyl]-3-yl)-5-oxopyrrolidine-2-carboxylate **S2** (71.8 mg, 0.21 mmo) in MeOH (1.6 mL) was added 6 M HCl (1.2 mL, aq.) followed by portion wise addition of Zn (139.7 mg, 2.14 mmol, 10.3 equiv.) over 5 min giving an orange solution. When full conversion was observed by TLC (15 min), the clear reaction mixture was quenched by careful addition of sat. $NaHCO_3$ (10 mL, aq.). The white suspension was extracted with CH_2Cl_2 (3 x 30 mL). The combined organic layers were dried over Na_2SO_4, filtered and concentrated *in vacuo*. The crude title product taken further without purification. R_f = 0.26 (CH_2Cl_2:MeOH (9:1), $KMnO_4$); ^{1}H-NMR (400 MHz, $CDCl_3$): δ 7.34-7.30 (2H, m, H-15), 7.26-7.21 (3H, m, H-14/H-16), 6.00-5.96 (1H, m, H-8), 5.59-5.55 (1H, m, H-9), 4.97-4.94 (1H, m, H-1), 4.49 (1H, dd, *J* 8.63, 1.84 Hz, H-7), 3.72 (3H, s, H-6), 3.44 (1H, dd, *J* 11.39, 5.78 Hz, H-12), 2.74 (1H, dt, *J* 11.39, 5.78 Hz, H-11), 2.64-2.55 (1H, m, H-10a), 2.47-2.37 (4H, m, H-3a/H-10b/H-17), 2.25-2.18 (2H, m, H-3b/H-4a), 2.02-1.94 (1H, m, H-4b); ^{13}C-NMR (100 MHz, $CDCl_3$): δ 177.0 (C-2), 174.1 (C-5), 143.1 (C-13), 132.0 (C-8), 128.9 (C-Ar), 127.9 (C-Ar), 127.0 (C-Ar), 123.8 (C-9), 60.5 (C-1), 54.6 (C-12), 52.3 (C-6), 49.5 (C-7), 44.8 (C-11), 33.7 (C-10), 29.6 (C-3), 23.8 (C-4); HRMS (ESI): *m/z* calcd for $C_{18}H_{23}N_2O_3$ $[M+H]^+$: 315.1709; found: 315.1709.

(3a*S*,5a*R*,6*R*,9a*S*)-6-Phenyl-3,3a,5a,6,7,9a-hexahydropyrrolo[1,2-*a*]quinoxaline-1,4(2*H*,5*H*)-dione (7)

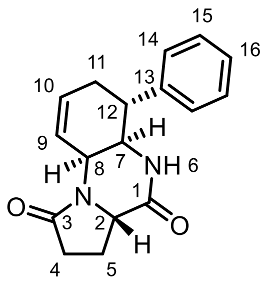

A solution of crude methyl (*S*)-1-((1*R*,2*R*,3*S*)-2-amino-1,2,3,6-tetrahydro-[1,1'-biphenyl]-3-yl)-5-oxopyrrolidine-2-carboxylate **S3** (26.5 mg, 0.084 mmol) in toluene (1.4 mL) was refluxed overnight. The reaction mixture was concentrated under reduced pressure and directly purified by flash column chromatography (CH_2Cl_2:MeOH 99:1 to 95:5) to afford (3a*S*,5a*R*,6*R*,9a*S*)-6-phenyl-3,3a,5a,6,7,9a-hexahydropyrrolo[1,2-*a*]quinoxaline-1,4(2*H*,5*H*)-dione **7** (15.2 mg, 54%, over two steps) as an off-white solid. R_f = 0.36 (CH_2Cl_2:MeOH (9:1), $KMnO_4$); IR (neat) ν (cm^{-1}): 3203, 3006, 2902, 1682, 1642, 1494, 1382, 747, 702; ^{1}H-NMR (400 MHz, $CDCl_3$): δ 7.36-7.32 (2H, m, H-15), 7.28-7.25 (1H, m, H-16), 7.22-7.20 (2H, m, H-14), 6.20-6.16 (1H, m, H-10), 6.04-6.00 (2H, m, H-6/H-9), 4.15-4.09 (2H, m, H-2/H-8), 3.77-3.72 (1H, m, H-7), 3.11-3.05 (1H, m, H-12), 2.57-2.32 (5H, m, H-4/H-5a/H-11), 2.16-2.05 (1H, m, H-5b); ^{13}C-NMR (100 MHz, $CDCl_3$): δ 174.4 (C-3), 170.3 (C-1), 140.6 (C-13), 129.3 (C-15), 128.2 (C-9), 127.8 (C-14), 127.7 (C-16), 122.8 (C-10), 58.9 (C-2), 55.7 (C-7), 49.7 (C-8), 42.7 (C-12), 31.8 (C-11), 31.0 (C-4), 21.7 (C-5); HRMS (ESI): *m/z* calcd for $C_{17}H_{19}N_2O_2$ $[M+H]^+$: 283.1447; found: 283.1449; $[\alpha]_D^{20}$ = -234.8 (c 0.4, $CHCl_3$).

Methyl (S,E)-5-oxo-1-(penta-2,4-dien-1-yl)pyrrolidine-2-carboxylate (S4)

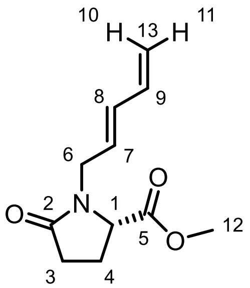

According to **general procedure C**, methyl (*S*)-(+)-2-pyrrolidone-5-carboxylate (833 µL, 7.13 mmol, 1.5 equiv) in DMF (5.1 mL) was added NaH (285.2 mg, 7.13 mmol, 1.5 equiv.) and freshly prepared (*E*)-5-bromopenta-1,3-diene[5] (4.76 mmol, 1.0 equiv.) in DMF (2.0 mL). The reaction mixture and stirred overnight at rt. The title compound was purified by flash column chromatography (EtOAc:$PE_{40\text{-}60}$ 1:3 to 3:2) to afford methyl (*S*,*E*)-5-oxo-1-(penta-2,4-dien-1-yl)pyrrolidine-2-carboxylate **S4** (512.7 mg, 52%, over two steps) as a clear oil. *Degradation of the material was observed within less than a month*. R_f = 0.45 (EtOAc:$PE_{40\text{-}60}$ (1:1), $KMnO_4$); IR (neat) ν (cm^{-1}): 2953, 1735, 1699, 1437, 1206; ^{1}H-NMR (400 MHz, $CDCl_3$): δ 6.29 (1H, dt, *J* 16.93, 10.21 Hz, H-9), 6.13 (1H, dd, *J* 15.01, 10.60 Hz, H-8), 5.58-5.51 (1H, m, H-7), 5.19 (1H, d, *J* 16.93 Hz, H-10), 5.09 (1H, d, *J* 10.07 Hz, H-11), 4.32 (1H, dd, *J* 15.27, 5.87 Hz, H-6a), 4.17-4.14 (1H, m, H-1), 3.73 (3H, s, H-12), 3.61 (1H, dd, *J* 15.27, 7.83 Hz, H-6b), 2.56-2.46 (1H, m, H-3a), 2.41-2.25 (2H, m, H-3b/H-4a), 2.10-2.04 (1H, m, H-4b); ^{13}C-NMR (100 MHz, $CDCl_3$): δ 174.9 (C-2), 172.6 (C-5), 135.9 (C-9), 134.9 (C-8), 127.3 (C-7), 118.2 (C-13), 59.1 (C-1), 52.5 (C-12), 43.6 (C-6), 29.6 (C-3), 23.0 (C-4); HRMS (ESI): *m/z* calcd for $C_{11}H_{16}NO_3$ $[M+H]^+$: 210.1125; found: 210.1123; $[\alpha]_D^{20}$ = -7.8 (c 0.6, $CHCl_3$).

(S,E)-N-Hydroxy-5-oxo-1-(penta-2,4-dien-1-yl)pyrrolidine-2-carboxamide (S5)

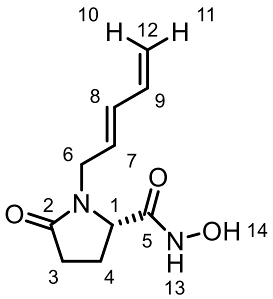

To a stirring solution of $NH_2OH{\cdot}HCl$ (298.9 mg, 4.30 mmol, 3.0 equiv.) in MeOH (6.0 mL) was added pulverized KOH (425.1 mg, 7.58 mmol, 5.3 equiv) and stirred at rt for 30 min. The mixture was added to a solution of methyl (*S*,*E*)-5-oxo-1-(penta-2,4-dien-1-yl)pyrrolidine-2-carboxylate **S4** (300.0 mg, 1.43 mmol) in MeOH (4.0 mL) and the reaction mixture was refluxed under nitrogen. When full conversion was observed by TLC (70 min), the pH of the reaction mixture was adjusted to ~ 4 by the addition of 25% AcOH (aq.) followed by evaporation at reduced pressure. Residues were redissolved in H_2O and extracted with EtOAc (4 x 20 mL). The combined organic layers were dried over Na_2SO_4, filtered and concentrated *in vacuo*. The title product **S5** (145.9 mg, 48%) was used without further purification. R_f = 0.22 (CH_2Cl_2:MeOH (9:1), $KMnO_4$); IR (neat) ν (cm^{-1}): 3129, 3019, 2760, 1645, 1539, 1461, 1254, 1157; ^{1}H-NMR (400 MHz, MeOD): δ 6.36 (1H, dt, *J* 16.90, 10.23 Hz, H-9), 6.21 (1H, dd, *J* 14.73, 10.82 Hz, H-8), 5.63-5.55 (1H, m, H-7), 5.24-5.08 (2H, m, H-10/H-11), 4.34-4.29 (1H, m, H-6a), 4.07-4.04 (1H, m, H-1), 3.42 (1H, dd, *J* 15.53, 7.83 Hz, H-6b), 2.61-2.53 (1H, m, H-3a), 2.43-2.27 (2H, m, H-3b/H-4a), 2.06-1.97 (1H, m, H-4b); ^{13}C-NMR (100 MHz, MeOD): δ 178.0 (C-2), 170.3 (C-5), 137.3 (C-9), 136.0 (C-8), 128.0 (C-7), 118.3 (C-12), 59.5 (C-1), 44.2 (C-6), 30.8 (C-3), 23.9 (C-4); HRMS (ESI): *m/z* calcd for $C_{10}H_{14}N_2NaO_3$ $[M+H]^+$: 233.0987; found: 233.0890; $[\alpha]_D^{20}$ = 2.0 (c 0.3, MeOH).

(9a*S*)-4a,5,9,9a-Tetrahydro-2*H*,7*H*-pyrrolo[1',2':4,5]pyrazino[1,2-*b*][1,2]oxazine-7,10(8*H*)-dione (8)

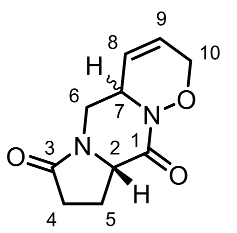

To a solution of (*S*,*E*)-*N*-hydroxy-5-oxo-1-(penta-2,4-dien-1-yl)pyrrolidine-2-carboxamide **S5** (50.0 mg, 0.24 mmol) in H_2O:MeOH (5:1, 2.16 mL) under vigorous stirring was added *n*Bu_4NIO_4 (181.4 mg, 0.42 mmol, 1.8 equiv.). When full conversion was observed by TLC (20 min), the reaction mixture was quenched by the addition of 10% $Na_2S_2O_3$ (aq., 5 mL) and extracted with CH_2Cl_2 (3 x 20 mL). The combined organic layers were dried with $MgSO_4$, filtered and concentrated *in vacuo*. The crude product was purified by flash column chromatography (CH_2Cl_2:MeOH 100:0 to 97.5:2.5) to afford (9a*S*)-4a,5,9,9a-tetrahydro-2*H*,7*H*-pyrrolo[1',2':4,5]pyrazino[1,2-b][1,2]oxazine-7,10(8*H*)-dione **8** (17.9 mg, 36%) as an off-white amorphous solid contaminated with *tetra*-butyl species. A 1:2 diastereomeric mixture was observed. R_f = 0.38 (CH_2Cl_2:MeOH (9:1), $KMnO_4$); IR (neat) ν (cm^{-1}): 3242, 2954, 1740, 1661, 1435, 1416, 1203, 1173; ^{1}H-NMR (400 MHz, $CDCl_3$): δ 5.95 (3/9H, m, H-9), 5.93 (6/9H, m, H-9), 5.87 (6/9H, m, H-8), 5.84 (3/9H, m, H-8), 4.82-4.77 (1H, m, H-10a), 4.61-4.57 (1H, m, H-7), 4.28-4-18 (3H, m, H-2/H-6a/H-10b), 3.42 (1H, dd, *J* 13.82, 5.32 Hz, H-6b), 2.45-2.35 (3H, m, H-4/H-5a), 2.32-2.22 (1H, m, H-5b); ^{13}C-NMR (100 MHz, $CDCl_3$): δ 173.4 (C-3), 167.7 (C-1), 127.5 (C-9), 126.2 (C-8), 67.9 (C-10), 58.4 (C-2), 56.6 (C-7), 40.9 (C-6), 29.9 (C-4), 20.5 (C-5); HRMS (ESI): *m/z* calcd for $C_{10}H_{13}N_2O_3$ $[M+H]^+$: 209.0921; found: 209.0912; $[\alpha]_D^{20}$ = 0.67 (c 0.6, $CHCl_3$).

Methyl (*S*)-5-oxo-1-(prop-2-yn-1-yl)pyrrolidine-2-carboxylate (S6)

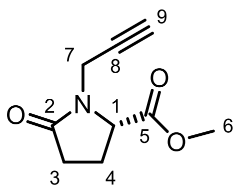

According to **general procedure C**, methyl (*S*)-(+)-2-pyrrolidone-5-carboxylate (1.7 mL, 14.56 mmol) in DMF (29 mL) was added NaH (698.8 mg, 17.47 mmol, 1.2 equiv.) and propargyl bromide (3.24 mL, 80% in toluene, 29.12 mmol, 2.0 equiv.). The reaction mixture was stirred overnight at rt. The title compound was purified by flash column chromatography (EtOAc:$PE_{40\text{-}60}$ 2:3 to 3:2) to afford methyl (*S*)-5-oxo-1-(prop-2-yn-1-yl)pyrrolidine-2-carboxylate **S6** (1.78 g, 67%) as a brown thick oil. R_f = 0.14 (EtOAc, $KMnO_4$); IR (neat) ν (cm^{-1}): 3272, 2957, 1738, 1685, 1437, 1409, 1195, 1177; ^{1}H-NMR (400 MHz, $CDCl_3$): δ 4.60 (1H, dd, *J* 17.71, 2.60 Hz, H-7a), 4.44-4.41 (1H, m, H-1), 3.82-3.75 (4H, m, H-6/H-7b), 2.52-2.34 (3H, m, H-3/H-4a), 2.23 (1H, t, *J* 2.62 Hz, H-9), 2.15-2.07 (1H, m, H-4b); ^{13}C-NMR (100 MHz, $CDCl_3$): δ 174.5 (C-2), 172.2 (C-5), 77.3 (C-8), 73.1 (C-9), 58.5 (C-1), 52.7 (C-6), 31.4 (C-7), 29.5 (C-3), 22.9 (C-4); HRMS (ESI): *m/z* calcd for $C_9H_{12}NO_3$ $[M+H]^+$: 182.0812; found: 182.0816; $[\alpha]_D^{20}$ = -16.5 (c 0.7, $CHCl_3$).

(*S*)-*N*-Methyl-5-oxo-1-(prop-2-yn-1-yl)pyrrolidine-2-carboxamide (S7)

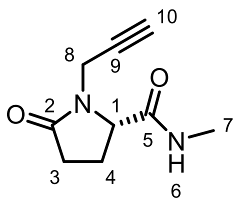

A solution of methyl (*S*)-5-oxo-1-(prop-2-yn-1-yl)pyrrolidine-2-carboxylate **S6** (348.8 mg, 1.93 mmol) in $MeNH_2$ (14.44 mL, 2.0 M in MeOH, 28.88 mmol, 15.0 equiv.) was stirred overnight. The reaction mixture was concentrated under reduced pressure and directly purified by flash column chromatography (CH_2Cl_2:MeOH 99:1 to 95:5) to afford (*S*)-*N*-methyl-5-oxo-1-(prop-2-yn-1-yl)pyrrolidine-2-carboxamide **S7** (277.9 mg, 80%) as an off-white amorphous solid. R_f = 0.19 (CH_2Cl_2:MeOH (9:1), $KMnO_4$); IR (neat) ν (cm^{-1}): 3337, 3204, 2928, 1680, 1654, 1564, 1438, 1403; ^{1}H-NMR (400 MHz, $CDCl_3$): δ 6.23 (1H, br s, H-6), 4.53-4.48 (1H, m, H-8a), 4.25-4.21 (1H, m, H-1), 3.74-3.69 (1H, m, H-8b), 2.86 (3H, d, *J* 4.86 Hz, H-7), 2.56-2.47 (1H, m, H-3a), 2.41-2.32 (2H, m, H-3b/H-4a), 2.26 (1H, t, *J* 2.51 Hz, H-10), 2.13-2.06 (1H, m, H-4b); ^{13}C-NMR (100 MHz, $CDCl_3$): δ 175.4 (C-2), 171.6 (C-5), 77.4

(C-9), 73.2 (C-10), 60.7 (C-1), 31.6 (C-8), 29.7 (C-3), 26.5 (C-7), 23.6 (C-4); HRMS (ESI): *m/z* calcd for $C_9H_{13}N_2O_2$ $[M+H]^+$: 181.0972; found: 181.0967; $[\alpha]_D^{20}$ = 4.7 (c 0.3, MeOH).

(*S*)-2,3-Dimethyl-8,8a-dihydropyrrolo[1,2-*a*]pyrazine-1,6(2*H*,7*H*)-dione (9)

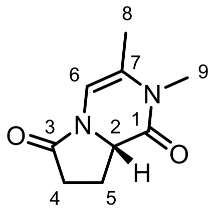

A microwave vial (2-5 mL) containing (*S*)-*N*-methyl-5-oxo-1-(prop-2-yn-1-yl)pyrrolidine-2-carboxamide **S7** (60.0 mg, 0.33 mmol), K_2CO_3 (92.0 mg, 0.67 mmol, 2.0 equiv.), pulverised KOH (20.5 mg, 0.37 mmol, 1.1 equiv.) and TBAB (21.5 mg, 0.067 mmol, 20 mol%) in THF (3.3 mL) was heated at 120 °C for 10 min. *Two identical reactions were performed and combined before aqueous work up*. Sat. NH_4Cl (3mL, aq.) was added and the resulting mixture was extracted with EtOAc (3 x 20 mL). The combined organic layers were dried over Na_2SO_4, filtered and concentrated *in vacuo*. The two crude isomerised products (112.8 mg, 0.63 mmol) were left on a vacuum pump for 2 h before a nitrogen atmosphere was established followed by addition of dried *p*TSA (53.9 mg, 0.31 mmol, 0.5 equiv.) and MeOH (6.3 mL). The reaction mixture was stirred overnight at rt. The following day, volatiles were removed under reduced pressure and redissolved in sat. NH_4Cl (5 mL, aq.) and extracted with EtOAc (3 x 30 mL). The combined organic layers were washed with sat. $NaHCO_3$ (10 mL, aq.) and dried over Na_2SO_4, filtered and concentrated *in vacuo*. The crude product was purified by flash column chromatography (EtOAc:PE_{40-60} 9:1) to afford (*S*)-2,3-dimethyl-8,8a-dihydropyrrolo[1,2-*a*]pyrazine-1,6(2*H*,7*H*)-dione **9** (54.8 mg, 46%) as an off-white solid. R_f = 0.19 (EtOAc, $KMnO_4$); IR (neat) ν (cm^{-1}): 3089, 2921, 1663, 1372, 1153; ^{1}H-NMR (400 MHz, $CDCl_3$): δ 6.26 (1H, s, H-6), 4.11 (1H, t, *J* 7.87 Hz, H-2), 3.14 (3H, s, H-9), 2.52-2.45 (3H, m, H-4/H-5a), 2.29-2.22 (1H, m, H-5b), 2.01 (3H, d, *J* 1.20 Hz, H-8); ^{13}C-NMR (100 MHz, $CDCl_3$): δ 172.0 (C-3), 166.7 (C-1), 125.3 (C-7), 104.4 (C-6), 56.7 (C-2), 30.8 (C-4), 29.1 (C-9), 22.5 (C-5), 16.6 (C-8); HRMS (ESI): *m/z* calcd for $C_9H_{13}N_2O_2$ $[M+H]^+$: 181.0972; found: 181.0967; $[\alpha]_D^{20}$ = 3.0 (c 0.3, $CHCl_3$).

Methyl (*S*)-1-((*S*/*R*)-2-nitro-1-phenylethyl)-5-oxopyrrolidine-2-carboxylate

To a solution of methyl (*S*)-(+)-2-pyrrolidone-5-carboxylate (141.0 µL, 1.21 mmol, 1.2 equiv.) in THF (8.0 mL) at -78 °C under nitrogen atmosphere was added *n*BuLi (616.0 µL, 1.6 M in hexanes, 0.99 mmol, 1.0 equiv.) dropwise and the mixture was kept at this temperature for 75 min. *trans*-β-Nitrostyrene (190.3 mg, 1.276 mmol, 1.3 equiv.) in THF (2.5 mL) was dropwise added to the cold solution and the reaction mixture was stirred at this temperature for 1 h before left at rt for 2h. The reaction mixture was quenched by the addition of NH_4Cl (4 mL) and extracted with Et_2O (3 x 40 mL). Combined organic layers were washed with H_2O, brine, dried over Na_2SO_4, filtered and concentrated *in vacuo*. The crude product was purified by flash column chromatography (EtOAc:PE_{40-60} 1:4 to 1:1) to afford the two diastereoisomers.

Methyl (*S*)-1-((*S*)-2-nitro-1-phenylethyl)-5-oxopyrrolidine-2-carboxylate (S8-*anti*)

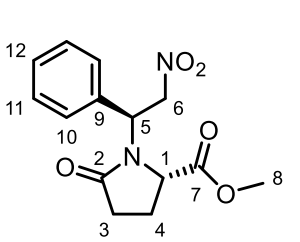

S,S-Diastereoisomer was afforded as a thick oil (53.2 mg, 18%). R_f = 0.23 (EtOAc:PE_{40-60} (1:1), $KMnO_4$); IR (neat) ν (cm^{-1}): 2956, 1738, 1689, 1549, 1377, 1206, 1177; ^{1}H-NMR (400 MHz, $CDCl_3$): δ 7.38-7.35 (3H, m, H-11/H-12), 7.32-7.30 (2H, m, H-10), 5.69 (1H, t, *J* 7.53 Hz, H-5), 5.38 (1H, dd, *J* 13.88, 7.53 Hz, H-6a), 4.94 (1H, dd, *J* 4.94 Hz, H-6b), 3.87 (1H, dd, *J* 9.21, 2.99 Hz, H-1), 3.60 (3H, s, H-8), 2.59 (1H, dt, *J* 16.95, 9.53 Hz, H-3a), 2.41 (1H, ddd, *J* 16.95, 9.53, 3.47 Hz, H-3b), 2.27-2.21 (1H, m, H-4a), 2.08-2.01 (1H, m, H-4b); ^{13}C-NMR (100 MHz,

$CDCl_3$): δ 175.7 (C-2), 172.9 (C-7), 134.0 (C-9), 129.32 (C-11), 129.31 (C-12), 128.1 (C-10), 74.2 (C-6), 60.0 (C-1), 55.9 (C-5), 52.7 (C-8), 30.1 (C-3), 23.8 (C-4); HRMS (ESI): *m/z* calcd for $C_{14}H_{17}N_2O_5$ $[M+H]^+$: 293,1133; found: 293.1137; $[\alpha]_D^{20}$ = -18.62 (c 0.29, $CHCl_3$).

Methyl (*S*)-1-((*R*)-2-nitro-1-phenylethyl)-5-oxopyrrolidine-2-carboxylate (S8-*syn*)

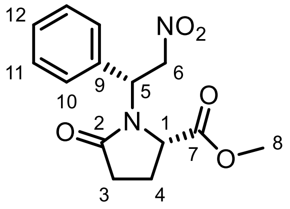

S,R-Diastereoisomer was afforded as a thick oil (83.6 mg, 29%). R_f = 0.19 (EtOAc:PE_{40-60} (1:1), $KMnO_4$); IR (neat) ν (cm^{-1}): 3028, 2956, 2928, 1736, 1681, 1551, 1380, 1207, 1179; ^{1}H-NMR (400 MHz, $CDCl_3$): δ 7.36-7.32 (5H, m, H-10/H-11/H-12), 5.55 (1H, t, *J* 7.34 Hz, H-5), 5.37 (1H, dd, *J* 12.46, 8.38 Hz, H-6a), 5.05 (1H, dd, *J* 12.46, 6.78 Hz, H-6b), 4.20 (1H, d, *J* 8.56 Hz, H-1), 3.46 (3H, s, H-8), 2.61-2.53 (1H, m, H-3a), 2.39-2.21 (2H, m, H-3b/H-4a), 2.08-2.02 (1H, m, H-4b); ^{13}C-NMR (100 MHz, $CDCl_3$): δ 176.0 (C-2), 172.0 (C-7), 134.5 (C-9), 129.3 (C-12), 129.2 (C-Ar), 128.2 (C-Ar), 75.4 (C-6), 59.2 (C-1), 55.7 (C-5), 52.5 (C-8), 29.9 (C-3), 23.9 (C-4); HRMS (ESI): *m/z* calcd $C_{14}H_{17}N_2O_5$ $[M+H]^+$: 293,1132; found: 293.1137; $[\alpha]_D^{20}$ = -45.17 (c 0.6, $CHCl_3$).

(4*S*,8a*S*)-4-Phenyltetrahydropyrrolo[1,2-*a*]pyrazine-1,6(2*H*,7*H*)-dione (10)

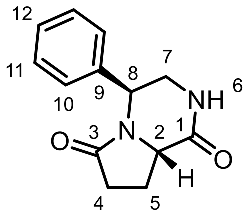

To a solution of methyl (*S*)-1-((*S*)-2-nitro-1-phenylethyl)-5-oxopyrrolidine-2-carboxylate **S8-*anti*** (44.0 mg, 0.15 mmol) in MeOH (1.0 mL) was added 6 M HCl (0.7 mL, aq.) followed by portion wise addition of Zn (83.5 mg, 1.28 mmol, 8.5 equiv.) over 5 min. When full conversion was observed by TLC (20 min), the reaction mixture was quenched by careful addition of sat. $NaHCO_3$ (7 mL, aq.). The white suspension was extracted with CH_2Cl_2 (3 x 30 mL). The combined organic layers were dried over Na_2SO_4, filtered and concentrated *in vacuo*. The crude product was purified by flash column chromatography (CH_2Cl_2:MeOH 99:1 to 97:3) to afford (4*S*,8a*S*)-4-phenyltetrahydropyrrolo[1,2-*a*]pyrazine-1,6(2*H*,7*H*)-dione **10** (15.4 mg, 44%) as a clear oil. R_f = 0.22 (CH_2Cl_2:MeOH (9:1), $KMnO_4$); IR (neat) ν (cm^{-1}): 3212, 2907, 2851, 1667, 749, 715; ^{1}H-NMR (400 MHz, $CDCl_3$): δ 7.39-7.26 (5H, m, H-10/H-11/H-12), 6.92 (1H, br s, H-6), 5.33 (1H, t, *J* 5.27 Hz, H-8), 4.11-4.07 (1H, m, H-2), 3.81-3.70 (2H, m, H-7), 2.55-2.47 (2H, m, H-4), 2.46-2.38 (1H, m, H-5a), 2.37-2.29 (1H, m, H-5b); ^{13}C-NMR (100 MHz, $CDCl_3$): δ 173.9 (C-3), 171.2 (C-1), 137.1 (C-9), 129.2 (C-Ar), 128.4 (C-12), 126.6 (C-Ar), 55.5 (C-2), 50.0 (C-8), 44.4 (C-7), 30.5 (C-4), 20.9 (C-5),; HRMS (ESI): *m/z* calcd for $C_{13}H_{15}N_2O_2$ $[M+H]^+$: 231.1134; found: 231.1137; $[\alpha]_D^{20}$ = 43.0 (c 0.5, $CHCl_3$).

Methyl (*S*)-1-(2-cyano-4-nitrophenyl)-5-oxopyrrolidine-2-carboxylate (11)

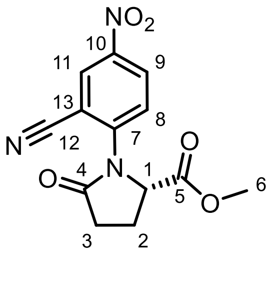

According to **general procedure C**, methyl (*S*)-(+)-2-pyrrolidone-5-carboxylate (295 µL, 2.53 mmol, 1.4 equiv) in DMF (4.4 mL) was added NaH (96.0 mg, 4.01 mmol, 2.3 equiv.) and 2-fluoro-5-nitrobenzonitrile (295.0 mg, 1.78 mmol). The reaction mixture was stirred overnight. The title compound was purified by flash column chromatography (EtOAc:PE_{40-60} 1:1 to 17:3) to afford methyl (*S*)-1-(2-cyano-4-nitrophenyl)-5-oxopyrrolidine-2-carboxylate **11** (166.4 mg, 32%) as a brown foam. R_f = 0.13 (EtOAc:PE_{40-60} (1:1), UV); IR (neat) ν (cm^{-1}): 3098, 2953, 2241, 1720, 1612, 1580, 1526, 1488, 1338, 1208, 1148; ^{1}H-NMR (400 MHz, $CDCl_3$): δ 8.56 (1H, d, *J* 2.44 Hz, H-11), 8.47 (1H, dd, *J* 8.94, 2.44 Hz, H-9), 7.75 (1H, d, *J* 8.94 Hz, H-8), 5.08-5.05 (1H, m, H-1), 3.73 (3H, s, H-6), 2.78-2.64 (3H, m, H-2a/H-3), 2.39-2.32 (1H, m,

H-2b); ^{13}C-NMR (100 MHz, $CDCl_3$): δ 174.5 (C-4), 171.1 (C-5), 146.0 (C-10/C-7), 129.9 (C-8), 129.0 (C-11), 128.4 (C-9), 114.8 (C-12), 111.9 (C-13), 61.5 (C-1), 53.1 (C-6), 29.7 (C-3), 23.9 (C-2); HRMS (ESI): *m/z* calcd for $C_{13}H_{11}N_3NaO_5$ $[M+H]^+$: 312.0591; found: 312.0587; $[\alpha]_D^{20}$ = -28.3 (c 0.35, $CHCl_3$).

(*S*)-2-(methoxycarbonyl)-5-Phenyl-3,4-dihydro-2*H*-pyrrole 1-oxide (S9)

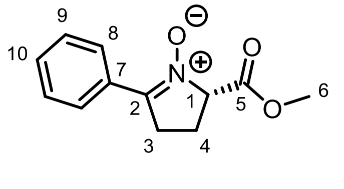

To a solution of urea hydrogen peroxide (3.0 equiv.). in MeOH (0.74 M) was added CH_3ReO_3 (2 mol%) and the yellow solution was stirred for 15 min before a solution of methyl (*S*)-5-phenyl-3,4-dihydro-2*H*-pyrrole-2-carboxylate (1.0 equiv.) in MeOH (0.167 M) was added. The reaction mixture stirred for 2 h and then concentrated under reduced pressure and treated with CH_2Cl_2. The precipitate was removed by filtration over celite and the filtrate was concentrated *in vacuo*. The yellow oil was used without further purification.

Methyl (3a*R*,6*S*,8a*S*,8b*S*)-2-methyl-1,3-dioxo-8a-phenyloctahydro-1*H*-dipyrrolo[1,2-*b*:3',4'-*d*]isoxazole-6-carboxylate (12)

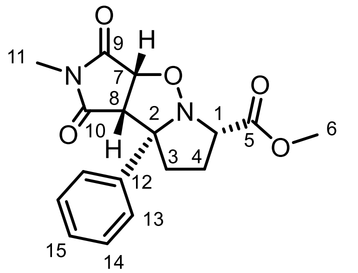

To a solution of **S9** (prepared from 201.8 mg of methyl (*S*)-5-phenyl-3,4-dihydro-2*H*-pyrrole-2-carboxylate) in CH_2Cl_2 (1.5 mL) was added *N*-methylmaleimide (120.2 mg, 1.08 mmol, 1.1 equiv.) followed by stirring overnight. The reaction mixture was concentrated under reduced pressure and the residues were dissolved in a minimal amount of boiling Et_2O (40 mL) and allowed to precipitate at rt. The title product was recrystallised from *i*PrOH (12 mL) to afford methyl (3a*R*,6*S*,8a*S*,8b*S*)-2-methyl-1,3-dioxo-8a-phenyloctahydro-1*H*-dipyrrolo[1,2-*b*:3',4'-*d*]isoxazole-6-carboxylate **12** (28.7 mg, 10%) as a white crystalline powder. R_f = 0.43 (CH_2Cl_2:MeOH = 99:1); IR (neat) ν (cm^{-1}): 2954, 1782, 1731, 1701, 1438, 1276, 1053; ^{1}H NMR (400 MHz, $CDCl_3$) δ 7.43 (2H, dd, *J* 8.2, 1.4 Hz, H-13), 7.32 – 7.23 (3H, m, H-14/H-15), 5.04 (1H, d, *J* 7.6 Hz, H-7), 4.17 (1H, t, *J* 8.6 Hz, H-1), 3.70 (1H, d, *J* 7.6 Hz, H-8), 3.67 (3H, s, H-6), 2.70 – 2.60 (1H, m, H-3a), 2.65 (3H, s, H-11), 2.55 – 2.47 (1H, m, H-3b), 2.42 – 2.32 (1H, m, H-4a), 2.09 – 1.99 (1H, m, H-4b); ^{13}C NMR (100 MHz, $CDCl_3$) δ 174.2 (C(O)), 172.4 (C(O)), 171.2 (C-5), 136.8 (C-12), 128.4 (2xAr), 126.9 (C-15), 81.4 (C-2), 78.2 (C-7), 68.5 (C-8), 58.6 (C-1), 52.5 (C-6), 35.7 (C-3), 26.9 (C-4), 24.6 (C-11); HRMS (ESI) *m/z* calcd for $C_{17}H_{19}N_2O_5$ $[M+H]^+$: 331.1288; found: 331.1292; $[\alpha]_D^{20}$ = -74.1° (*c* 0.45, CH_2Cl_2).

Dimethyl (3a*R*,6*S*)-3a-phenyl-3a,4,5,6-tetrahydropyrrolo[1,2-*b*]isoxazole-3,6-dicarboxylate (13)

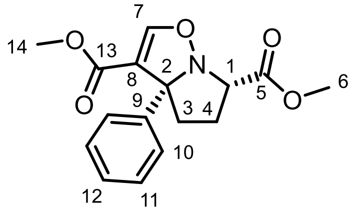

To a solution of **S9** (prepared from 100 mg of methyl (*S*)-5-phenyl-3,4-dihydro-2*H*-pyrrole-2-carboxylate) in CH_2Cl_2 (0.5 mL) was added methyl propiolate (50.0 µL, 0.54 mmol, 1.1 equiv.) and the reaction mixture was stirred overnight at rt. The crude material was purified by flash column chromatography (CH_2Cl_2:MeOH 99:1) to afford dimethyl (3a*R*,6*S*)-3a-phenyl-3a,4,5,6-tetrahydropyrrolo[1,2-*b*]isoxazole-3,6-dicarboxylate **13** (78.7 mg, 53%, over two steps) as a brown oil. R_f = 0.68 (CH_2Cl_2:MeOH = 99:1); IR (neat) ν (cm^{-1}): 2961, 1738, 1706, 1619, 1437, 1259, 1080, 1014, 792; ^{1}H-NMR (400 MHz, $CDCl_3$) δ 7.61 (2H, d, *J* 7.3 Hz, H-10), 7.36 (1H, s, H-7), 7.32 (2H, t, *J* 7.5 Hz, H-11), 7.23 (1H, t, *J* 7.3 Hz, H-12), 4.07 (1H, t, *J* 7.6 Hz, H-1), 3.76 (3H, s, H-6), 3.69 (3H, s, H-14), 2.86 (1H, dt, *J* 13.4, 7.6 Hz, H-3a), 2.62 – 2.56 (1H, m, H-3b), 2.25 – 2.09 (2H, m,

H-4); ^{13}C NMR (100 MHz, $CDCl_3$) δ 171.5 (C-5), 163.9 (C-13), 152.6 (C-7), 144.1 (C-9), 128.3 (C-Ar), 127.4 (C-Ar), 126.3 (C-Ar), 112.7 (C-8), 79.8 (C-2), 72.0 (C-1), 52.6 (OCH_3), 51.4 (OCH_3), 35.6 (C-3), 27.0 (C-4); HRMS (ESI) *m/z* calcd for $C_{16}H_{18}NO_5$ $[M+H]^+$: 304.1179; found: 304.1176; $[\alpha]_D^{20}$ = -332.3° (c 0.74, CH_2Cl_2).

1-(*tert*-butyl) 2-Methyl (2*S*,5*S*)-5-((trimethylsilyl)ethynyl)pyrrolidine-1,2-dicarboxylate[6] (S10)

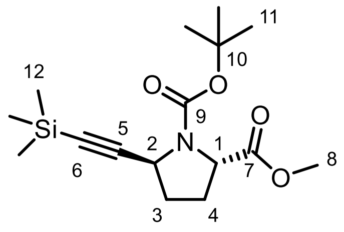

Adaptation of literature procedure developed by Claraz *et al.*[7] To a stirring solution of $CuBr{\cdot}Me_2S$ (6.21 g, 30.21 mmol, 3.0 equiv.) in Et_2O (45 mL) at -40 °C was added freshly prepared ((trimethylsilyl)ethynyl)magnesium bromide[8] (26.3 mL, 30.21 mmol, 1.15 M in THF, 3.0 equiv.) under nitrogen atmosphere and stirred for 1.5 h at this temperature before cooled to -78 °C. $BF_3{\cdot}OEt_2$ (3.73 mL, 30.21 mmol, 3.0 equiv.) was added and followed by subsequently addition of 1-(*tert*-butyl) 2-methyl (2*S*)-5-methoxypyrrolidine-1,2-dicarboxylate[9] (2.61 g, 10.07 mmol) in Et_2O (20 mL) and the mixture was stirred for 40 min at this temperature. The reaction mixture was then stirred at 0 °C. When full conversion was observed by TLC (7 h), the reaction mixture was quenched by the addition of a solution of sat. NH_4Cl (aq.) and NH_4OH (aq.) (150 mL, 1:1) and stirred at rt for 30 min. The solution was diluted with H_2O (100 mL) and Et_2O (100 mL) and the aqueous layer was extracted with Et_2O (2 x 200 mL). The combined organic layers were dried over Na_2SO_4, filtered and concentrated *in vacuo*. The crude product was purified by flash column chromatography (EtOAc:PE_{40-60} 1:19 to 3:17) to afford 1-(*tert*-butyl) 2-methyl (2*S*,5*S*)-5-((trimethylsilyl)ethynyl)pyrrolidine-1,2-decarboxylate **S10** (2.89 g, 88%) as a yellow thick oil. A ~ 1:2 mixture of rotamers was observed.
R_f = 0.54 (EtOAc:PE_{40-60} (1:3), $KMnO_4$); IR (neat) ν (cm^{-1}): 2957, 2171, 1748, 1700, 1385 1163; ^1H-NMR (400 MHz, $CDCl_3$): δ 4.72 (1/3H, d, *J* 7.85 Hz, H-1), 4.59 (2/3H, d, *J* 7.85 Hz, H-1), 4.41 (2/3H, d, *J* 8.94 Hz, H-2), 4.31 (1/3H, d, *J* 8.94 Hz, H-2), 3.71-3.70 (3H, m, H-8), 2.54-2.36 (1H, m, H-4a), 2.26-2.13 (1H, m, H-3a), 2.01-1.96 (2H, m, H-3b/H-4b), 1.48 (6H, s, H-11), 1.41 (3H, s, H-11), 0.14 (9H, m, H-12); ^{13}C-NMR (100 MHz, $CDCl_3$): δ 173.5/173.2 (C-7), 153.9/153.0 (C-9), 106.0 (C-6), 86.1 (C-5), 80.6/80.5 (C-10), 59.1/58.6 (C-1), 52.4/52.2 (C-8), 49.6/49.5 (C-2), 32.0/31.5 (C-3), 29.7/28.7 (C-4), 28.5/28.4 (C-11), 0.1 (C-12); LCMS (ESI): *m/z* calcd for $C_{16}H_{27}NNaO_4Si$ $[M+Na]^+$ 348.5, found: 348.4; $[\alpha]^{20}_D$ = -126.6 (c 1.84, $CHCl_3$), lit.[6] $[\alpha]_D^{20}$ = -126.0 (c 1.84, $CHCl_3$).

1-(*tert*-butyl) 2-Methyl (2*S*,5*S*)-5-ethynylpyrrolidine-1,2-dicarboxylate (S11)

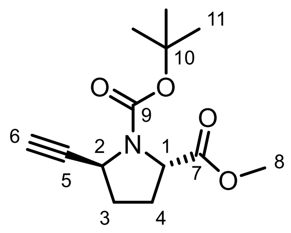

To a solution of 1-(*tert*-butyl) 2-methyl (2*S*,5*S*)-5-((trimethylsilyl)ethynyl)pyroli-dine-1,2-decarboxylate **S10** (2.00 g, 6.15 mmol) in MeOH (14 mL) was added K_2CO_3 (1.02 g, 7.40 mmol, 1.2 equiv.) and the mixture was vigorously stirred. When full conversion was observed by TLC (75 min), the reaction mixture was quenched by the addition of sat. NH_4Cl (10 mL, aq.) and MeOH was removed under reduced pressure. The residual solution was extracted with Et_2O (3 x 40 mL). The combined organic layers were washed with H_2O, brine, dried over $MgSO_4$, filtered and concentrated *in vacuo*. This afforded 1-(*tert*-butyl) 2-methyl (2*S*,5*S*)-5-ethynylpyrrolidine-1,2-dicarbox-ylate **S11** (1.52 g, 97%) as off-white amorphous solid that was used without further purification. A ~ 1:1 mixture of rotamers was observed; R_f = 0.57 (EtOAc:PE_{40-60} (1:1), $KMnO_4$); IR (neat) ν (cm^{-1}): 3259, 2979, 1745, 1697, 1379, 1365, 1161; ^1H-NMR (400 MHz, $CDCl_3$): δ 4.72 (11/20H, d, *J* 8.15, 0.55 H, H-2), 4.66 (9/20H, d, *J* 8.15 Hz, H-2), 4.40 (9/20H, d, *J* 8.99 Hz, H-1), 4.32 (11/20H, d, *J* 8.99 Hz, H-

1), 3.714-3.708 (3H, m, H-8), 2.54-2.38 (1H, m, H-4a), 2.29-2.19 (2H, m, H-3a/H-6), 2.05-1.96 (2H, m, H-4b/H-3b), 1.48 (9/20H, s, H-11), 1.41 (11/20H, s, H-11); ^{13}C-NMR (100 MHz, $CDCl_3$): δ 173.3/173.1 (C-7), 153.8/153.2 (C-9), 84.1/83.7 (C-5), 80.83/80.79 (C-10), 70.5/70.0 (C-6), 59.1/58.6 (C-1), 52.4/52.2 (C-8), 48.9/48.6 (C-2), 32.0/31.1 (C-3), 29.6/28.6 (C-4), 28.5/28.4 (C-11); HRMS (ESI): *m/z* calcd for $C_{13}H_{19}NNaO_4$ $[M+H]^+$: 276.1206; found: 276.1211; $[\alpha]_D^{20}$= -118.0 (c 0.5, $CHCl_3$).

Methyl (2*S*,5*S*)-5-ethynylpyrrolidine-2-carboxylate[10] (S12)

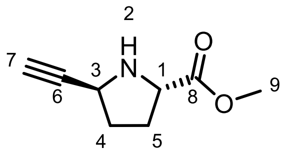

To a solution of 1-(*tert*-butyl) 2-methyl (2*S*,5*S*)-5-ethynylpyrrolidine-1,2-dicarboxylate **S11** (395.5 mg, 1.56 mmol) in CH_2Cl_2 (5.2 mL) in an open system at 0 °C was dropwise added TFA (1.195 mL, 15.61 mmol, 10.0 equiv.) and the reaction mixture was stirred at rt. When full conversion was observed by TLC (1 h), the reaction mixture was quenched by the addition of H_2O (5 mL) and $NaHCO_3$ (1.57 g, 18.74 mmol, 12.0 equiv.) (*caution, violent reaction occurs during addition*) and extracted with CH_2Cl_2 (5 x 20 mL). The combined organic layers were dried over Na_2SO_4, filtered and concentrated *in vacuo*. This afforded crude methyl (2*S*,5*S*)-5-ethynylpyrrolidine-2-carboxylate **S12** (471.1 mg, 93%) as a brown oil that was used without further purification. R_f = 0.34 (CH_2Cl_2:MeOH (9:1), $KMnO_4$); IR (neat) ν (cm^{-1}): 3260, 2952, 2070, 1738, 1673, 1202, 1175; H-NMR (400 MHz, $CDCl_3$): δ 4.05 (1H, t, *J* 5.93 Hz, H-3), 3.97 (1H, dd, *J* 7.63, 5.07 Hz, H-1), 3.72 (3H, s, H-9), 2.36-2.26 (3H, m, H-2/H-4a/H-7), 2.13-2.05 (1H, m, H-5a), 1.96-1.86 (2H, m, H-4b/H-5b); ^{13}C-NMR (100 MHz, $CDCl_3$): δ 175.5 (C.8), 85.8 (C-6), 70.7 (C-7), 58.9 (C-1), 52.4 (C-9), 48.6 (C-3), 32.6 (C-5), 29.1 (C-4); LCMS (ESI): *m/z* calcd for $C_8H_{12}NO_2$ $[M+H]^+$: 154.1; found: 154.0.

Methyl (2*S*,5*S*)-1-(*N*-(*tert*-butoxycarbonyl)-*N*-methylglycyl)-5-ethynylpyrrolidine-2-carboxylate (S13)

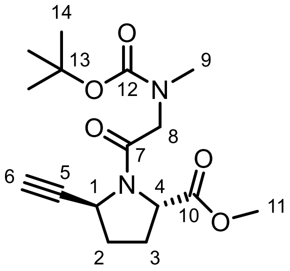

According to **general procedure B**, methyl (2*S*,5*S*)-5-ethynylpyrrolidine-2-carboxylate **S12** (100.7 mg, 0.66 mmol), *N*-(*tert*-butoxycarbonyl)-*N*-methylglycine (141.2 mg, 0.75 mmol, 1.1 equiv.), EDC·HCl (189.0 mg, 0.99 mmol, 1.5 equiv.) and Oxyma (140.1 mg, 0.99 mmol, 1.5 equiv.) in CH_2Cl_2 (24 mL) were stirred overnight. The crude product was purified by flash column chromatography (EtOAc:$PE_{40\text{-}60}$ 2:3 to 11:9) to afford methyl (2*S*,5*S*)-1-(*N*-(*tert*-butoxycarbonyl)-*N*-methylglycyl)-5-ethynylpyrrolidine-2-carboxylate **S13** (121.9 mg, 57%, over two steps) as an orange oil and used without further purification. A 1:1 mixture of rotamers was observed. R_f = 0.26 (EtOAc:$PE_{40\text{-}60}$ (1:1), $KMnO_4$); IR (neat) ν (cm^{-1}): 3252, 2972, 2950, 1736, 1706, 1653, 1389, 1326, 1149; ^{1}H-NMR (400 MHz, $CDCl_3$): δ 4.70 (1/2H, d, *J* 5.88 Hz, H-1), 4.62 (1/2H, d, *J* 5.88 Hz, H-1), 4.58-4.56 (1H, m, H-4), 4.37 (1/2H, m, H-8a), 4.15-4.08 (3/2H, m, H-8a/H-8b), 3.75 (2/5H, s, H-11), 3.69 (13/5H, s, H-11), 2.92-2.90 (3H, m, H-9), 2.40-2.35 (3H, m, H-2a/H-3a/H-6), 2.13-2.03 (2H, m, H-2b/H-3b), 1.45-1.42 (9H, m, H-14); ^{13}C-NMR (100 MHz, $CDCl_3$): δ 172.22/172.17 (C-10), 168.1 (C-7), 156.4/155.9 (C-12), 82.2/82.1 (C-5), 80.1/80.0 (C-13), 73.0/72.7 (C-6), 59.1/59.0 (C-4), 52.9/52.4 (C-11), 51.5/50.7 (C-8), 48.2/48.1 (C-1), 35.7/35.7 (C-9), 32.7 (C-2), 28.5/28.3 (C-14), 27.6 (C-3,); HRMS (ESI): *m/z* calcd for $C_{16}H_{24}N_2NaO_5$ $[M+H]^+$: 347.1577; found: 347.1581; $[\alpha]_D^{20}$ = -92.86 (c 0.35, $CHCl_3$).

(6*S*,8a*S*)-6-Ethynyl-2-methylhexahydropyrrolo[1,2-*a*]pyrazine-1,4-dione (14)

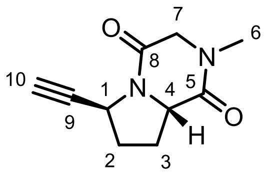

To a solution of methyl (2*S*,5*S*)-1-(*N*-(*tert*-butoxycarbonyl)-*N*-methylglycyl)-5-ethynylpyrrolidine-2-carboxylate **S13** (90.6 mg, 0.28 mmol) in CH_2Cl_2 (2.5 mL) in an open system at 0 °C was dropwise added TFA (214 µL, 2.79 mmol, 10.0 equiv.) and the reaction mixture was stirred at rt. When full conversion was observed by TLC (100 min), the reaction mixture was quenched by the addition of H_2O (5 mL) and $NaHCO_3$ (281.6 mg, 3.35 mmol, 12.0 equiv.) and the aqueous layer was extracted with CH_2Cl_2 (3 x 30 mL). The combined organic layers were dried over Na_2SO_4, filtered and concentrated *in vacuo*. The crude product was purified by flash column chromatography (CH_2Cl_2:MeOH 49:1 to 48:3) to afford (6*S*,8a*S*)-6-ethynyl-2-methylhexahydropyrrolo[1,2-*a*]pyrazine-1,4-dione **14** (34.8 mg, 65%) as an orange oil. R_f = 0.10 (CH_2Cl_2:MeOH (9:1), $KMnO_4$); IR (neat) ν (cm^{-1}): 3255, 2940, 2114, 1645, 1441, 1205; ^{1}H-NMR (400 MHz, $CDCl_3$): δ 4.83-4.79 (1H, m, H-1), 4.28-4.24 (1H, m, H-4), 4.22 (1H, dd, *J* 16.66, 1.70 Hz, H-7a), 3.81 (1H, d, *J* 16.66 Hz, H-7b), 2.98 (3H, s, H-6), 2.53-2.45 (1H, m, H-3a), 2.36 (1H, d, *J* 2.20 Hz, H-10), 2.33-2.25 (1H, m, H-2a), 2.16 (1H, dq, *J* 12.71, 8.23 Hz, H-3b), 2.08-2.01 (1H, m, H-2b); ^{13}C-NMR (100 MHz, $CDCl_3$): δ 166.7 (C-5), 162.2 (C-8), 81.8 (C-9), 71.9 (C-10), 58.2 (C-4), 53.6 (C-7), 47.8 (C-1), 33.8 (C-6), 31.0 (C-2), 28.3 (C-3); HRMS (ESI): *m/z* calcd for $C_{10}H_{13}N_2O_2$ $[M+H]^+$: 193.0972; found: 193.0969; $[\alpha]_D^{20}$ = -184.6 (c 0.35, MeOH).

Methyl (2*S*,5*S*)-1-(but-3-enoyl)-5-ethynylpyrrolidine-2-carboxylate (S14)

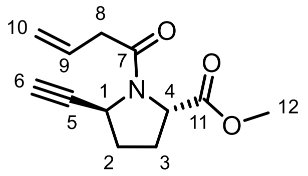

According to **general procedure B**, crude methyl (2*S*,5*S*)-5-ethynylpyrrolidine-2-carboxylate **S12** (92.0 mg, 0.60 mmol), 3-butenoic acid (61.3 µL, 0.72 mmol, 1.2 equiv.), EDC·HCl (172.7 mg, 0.90 mmol, 1.5 equiv.) and Oxyma (128.0 mg, 0.90 mmol, 1.5 equiv.) in CH_2Cl_2 (22 mL) were stirred overnight. The crude product was purified by flash column chromatography (EtOAc:$PE_{40\text{-}60}$ 4:14 to 7:13) to afford methyl (2*S*,5*S*)-1-(but-3-enoyl)-5-ethynylpyrrolidine-2-carboxylate **S14** (116.1 mg, 87%, over two steps) as an orange oil. A 1:9 mixture of rotamers was observed, however only the major was assigned. *Minor impurities were observed by ^{1}H-NMR*. R_f = 0.17 (EtOAc:$PE_{40\text{-}60}$ (1:3), $KMnO_4$); IR (neat) ν (cm^{-1}): 3245, 2954, 2115, 1741, 1649, 1431, 1173; ^{1}H-NMR (400 MHz, $CDCl_3$): δ 6.02-5.92 (1H, m, H-9), 5.25 (1/2H, q, *J* 1.56 Hz, H-10), 5.21-5.20 (1H, m, H-10), 5.18 (1/2H, q, *J* 1.56 Hz, H-10), 4.72-4.69 (1H, m, H-1), 4.57-4.54 (1H, m, H-4), 3.71 (3H, s, H-12), 3.33 (2H, dq, *J* 10.80, 2.16, 1.43 Hz, H-8), 2.47-2.32 (3H, m, H-2a, H-3a, H-6), 2.14-2.09 (1H, m, H-2b), 2.06-2.01 (1H, m, H-3b); ^{13}C-NMR (100 MHz, $CDCl_3$): δ 172.3 (C-11), 170.3 (C-7), 130.7 (C-9), 118.2 (C-10), 82.5 (C-5), 72.4 (C-6), 58.6 (C-4), 52.3 (C-12), 48.8 (C-1), 39.2 (C-8), 32.4 (C-2), 27.7 (C-3); HRMS (ESI): *m/z* calcd for $C_{12}H_{16}NO_3$ $[M+H]^+$: 222.1125; found: 222.1117; $[\alpha]_D^{20}$ = -116.0 (c 0.3, $CHCl_3$).

Methyl (3*S*,9b*S*)-5,8-dioxo-2,3,5,6,6a,7,8,9b-octahydro-1H-cyclopenta[*g*]indolizine-3-carboxylate (15)

To a solution of $Co_2(CO)_8$ (111.9 mg, 0.33 mmol, 1.25 equiv.) in CH_2Cl_2 (10 mL) was added methyl (2*S*,5*S*)-1-(but-3-enoyl)-5-ethynylpyrrolidine-2-carboxylate **S14** (57.9 mg, 0.26 mmol) in CH_2Cl_2 (3.0 mL) and stirred under nitrogen atmosphere. When full conversion was observed by TLC (3 h), NMO (894.8 mg, 2.617 mmol, 10 equiv.) was added portion wise over 10 min to the black solution and the reaction was stirred overnight. The violet precipitates were removed by filtration through a silica plug using

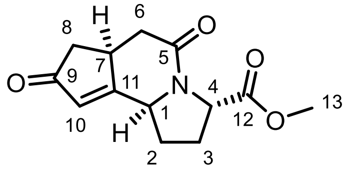

CH_2Cl_2:MeOH (19:1). The desired fractions were concentrated under reduced pressure and the crude product was purified by flash column chromatography (CH_2Cl_2:MeOH 100:0 to 97.2:2.8) to afford methyl (3*S*,9b*S*)-5,8-dioxo-2,3,5,6,6a,7,8,9b-octahydro-1H-cyclopenta[*g*]indolizine-3-carboxylate **15** (44.4 mg, 68%,) as an off-white solid. R_f = 0.72 (EtOAc:PE_{40-60} (1:1), $KMnO_4$); IR (neat) ν (cm^{-1}): 2922, 2852, 1747, 1703, 1690, 1630, 1444, 1175. ^{1}H-NMR (400 MHz, $CDCl_3$): δ 6.04-6.03 (1H, dt, *J* 1.81, 0.47 Hz, H-10), 4.65 (1H, t, *J* 8.57 Hz, H-4), 4.55 (1H, dd, *J* 11.07, 5.07 Hz, H-1), 3.77 (3H, s, H-13), 3.44-3.36 (1H, m, H-7), 3.03 (1H, dd, *J* 17.60, 7.81 Hz, H-6a), 2.74 (1H, dd, *J* 18.94, 6.59 Hz, H-8a), 2.54-2.46 (1H, m, H-3a), 2.43-2.37 (1H, m, H-2a), 2.16-2.08 (2H, m, H-6b/H-8b), 2.01-1.91 (1H, m, H-3b), 1.82 (1H, dq, *J* 11.56, 7.84 Hz, H-2b); ^{13}C-NMR (100 MHz, $CDCl_3$): δ 206.9 (C-9), 173.2 (C-5), 172.6 (C-12), 166.9 (C-11), 127.5 (C-10), 59.1 (C-1), 57.5 (C-4), 52.6 (C-13), 42.0 (C-8), 38.6 (C-6), 37.0 (C-7), 29.8 (C-2), 27.5 (C-3); HRMS (ESI): *m/z* calcd for $C_{13}H_{16}NO_4$ $[M+H]^+$ 250.1079: found: 250.1071; $[\alpha]_D^{20}$ = -11.1 (c 0.28, $CHCl_3$).

Methyl (2*S*,5*S*)-1-(2-azidoacetyl)-5-ethynylpyrrolidine-2-carboxylate (S15)

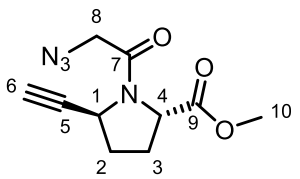

According to **general procedure B**, methyl (2*S*,5*S*)-5-ethynylpyrrolidine-2-carboxylate **S12** (60.6 mg, 0.40 mmol), azidoacetic acid (35.5 µL, 0.48 mmol, 1.2 equiv.), EDC·HCl (114.0 mg, 0.60 mmol, 1.5 equiv.) and Oxyma (85.0 mg, 0.60 mmol, 1.5 equiv.) in CH_2Cl_2 (15 mL) were stirred overnight. The crude product was purified by flash column chromatography (EtOAc:PE_{40-60} 1:4 to 3:2) to afford a mixture of methyl (2*S*,5*S*)-1-(2-azidoacetyl)-5-ethynylpyrrolidine-2-carboxylate and its spontaneous cyclised product. The mixture was carried through to the subsequently cyclisation step.

Methyl (8*S*,10a*S*)-6-oxo-5,6,8,9,10,10a-hexahydropyrrolo[1,2-*a*][1,2,3]triazolo[5,1-*c*]pyrazine-8-carboxylate (16)

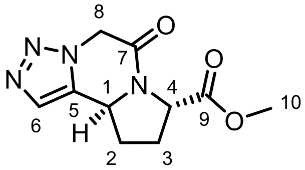

The partly cyclized mixture of methyl (2S,5S)-1-(2-azidoacetyl)-5-ethynylpyrrolidine-2-carboxylate **S15** (56.0 mg) was refluxed in $CHCl_3$ (7.0 mL) in an open system overnight. The reaction mixture was concentrated under reduced pressure and the crude product was purified by flash column chromatography (CH_2Cl_2:MeOH 100:0 to 99:1) to afford methyl (8*S*,10a*S*)-6-oxo-5,6,8,9,10,10a-hexahydropyrrolo[1,2-*a*][1,2,3]triazolo[5,1-*c*]pyrazine-8-carboxylate **16** (45.2 mg, 48%, over three steps) as an off-white solid. R_f = 0.41 (CH_2Cl_2:MeOH (9:1), $KMnO_4$); IR (neat) ν (cm^{-1}): 2951, 1746, 1652, 1441, 1195, 1173; ^{1}H-NMR (400 MHz, $CDCl_3$): δ 7.63 (1H, s, H-6), 5.22 (1H, d, *J* 17.18 Hz, H-8a), 5.04-4.94 (2H, m, H-1/H-8b), 4.65 (1H, t, *J* 8.37 Hz, H-4), 3.80 (3H. s, H-10), 2.70-2.56 (2H, m, H-2a/H-3a), 2.15-2.05 (1H, m, H-3b), 1.93 (1H, dq, *J* 11.46, 7.78 Hz, H-2b); ^{13}C-NMR (100 MHz, $CDCl_3$): δ 171.7 (C-9), 161.2 (C-7), 132.0 (C-5), 129.0 (C-6), 57.6 (C-4), 53.5 (C-1), 52.9 (C-10), 49.6 (C-8), 31.3 (C-2), 28.0 (C-3); HRMS (ESI): *m/z* calcd for $C_{10}H_{13}N_4O_3$ $[M+H]^+$: 237.0982; found: 237.0976; $[\alpha]_D^{20}$ = -192.5 (c 0.36, $CHCl_3$).

1-(*tert*-butyl) 2-Methyl (2*S*,5*S*)-5-(1-(2-ethoxy-2-oxoethyl)-1*H*-1,2,3-triazol-4-yl)pyrrolidine-1,2-dicarboxylate (17)

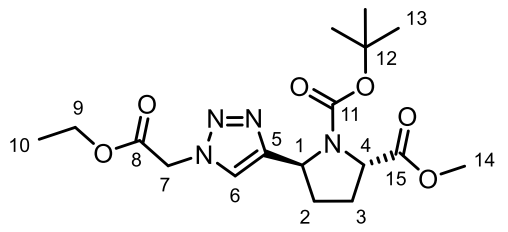

To a solution of 1-(*tert*-butyl) 2-methyl (2*S*,5*S*)-5-ethynylpyrrolidine-1,2-dicarboxylate **S11** (70.0 mg, 0.28 mmol) and ethyl azidoacetate (45.7 mg, 0.35 mmol, 1.3 equiv.) in *t*BuOH:H_2O (2.8 mL, 1:1) was added $CuSO_4 \cdot H_2O$ (6.9 mg, 0.028 mmol, 10 mol%) and NaOAs (16.4 mg, 0.083 mmol, 30 mol%) under nitrogen atmosphere and the reaction mixture was stirred overnight at 40 °C. The following day, the reaction mixture was quenched by the addition of 1 M HCl (aq.) to pH ~ 2-3 and extracted with EtOAc (3 x 20 mL). The combined organic layers were washed with brine, dried over Na_2SO_4, filtered and concentrated *in vacuo*. The crude product was purified by flash column chromatography (EtOAc:PE_{40-60} 3:7 to 11:9) to afford 1-(*tert*-butyl) 2-methyl (2*S*,5*S*)-5-(1-(2-ethoxy-2-oxoethyl)-1*H*-1,2,3-triazol-4-yl)pyrrolidine-1,2-dicarboxylate **17** (73.2 mg, 69%) as a yellow thick oil. A 1:2 mixture of rotamers was observed. R_f = 0.33 (EtOAc:PE_{40-60} (1:1), $KMnO_4$); IR (neat) ν (cm^{-1}): 3141, 2978, 1744, 1691, 1386, 1365, 1200, 1161; ^{1}H-NMR (400 MHz, $CDCl_3$): δ 7.67 (2/3H, s, H-6), 7.49 (1/3H, s, H-6), 5.25-5.23 (2/3H, m, H-1), 5.20 (1/3H, m, *J* 7.32 Hz, H-1), 5.13-5.09 (2H, m, H-7), 4.50-4.48 (1/3H, m, H-4), 4.39 (2/3H, dd, *J* 9.10, 0.74 Hz, H-4), 4.26 (2H, dq, *J* 7.12, 5.45 Hz, H-9), 3.754 (1H, s, H-14), 3.745 (2H, s, H-14), 2.72-2.62 (1H, m, H-3a), 2.46-2.33 (2H, m, H-2), 2.09-2.01 (1H, m, H-3b), 1.38 (6H, s, H-13), 1.37 (3H, s, H-13), 1.32-1.28 (3H, m H-10); ^{13}C-NMR (100 MHz, $CDCl_3$): δ 173.8/173.3 (C-15), 166.5/166.3 (C-8), 154.1/153.8 (C-11), 150.9/149.4 (C-5), 123.9/122.5 (C-6), 80.7/80.5 (C-12), 62.52/62.47 (C-9), 59.9/59.4 (C-4), 54.1/53.4 (C-1), 52.4/52.2 (C-14), 31.1/29.7 (C-3), 29.1/28.4 (C-2), 28.2 (C-13), 14.23/14.19 (C-10); HRMS (ESI): *m/z* calcd for $C_{17}H_{26}N_4NaO_6$ $[M+H]^+$: 405.1745; found: 405.1751; $[\alpha]_D^{20}$ = -54.4 (c 0.25, $CHCl_3$).

1-(*tert*-butyl) 2-Methyl (2*S*,5*S*)-5-((1-(tert-butoxycarbonyl)-1,2,3,6-tetrahydropyridin-4-yl)ethynyl)pyrrolidine-1,2-dicarboxylate (18)

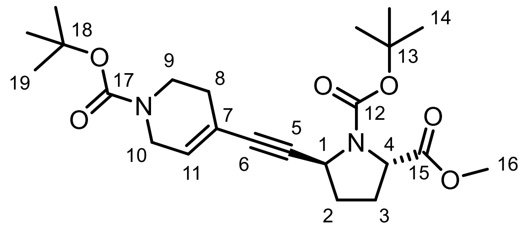

A round-bottomed flask containing $PdCl_2(PPh_3)_2$ (6.9 mg, 0.0099 mmol, 5 mol%) was evacuated with three times vacuum/nitrogen atmosphere before CuI (1.9 mg, 0.0099 mmol, 5 mol%) was added followed by degassed THF (1.2 mL) and degassed Et_3N (0.3 mL). A mixture of 1-(*tert*-butyl) 2-methyl (2*S*,5*S*)-5-ethynylpyrrolidine-1,2-dicarboxylate **S11** (51.6 mg, 0.20 mmol, 1.0 equiv.) and 1-(*tert*-butoxycarbonyl)-1,2,3,6-tetrahydropyridin-4-yl trifluoromethanesulfonate (65.4 mg, 0.197 mmol) in degassed THF (0.5 mL) was added to the Pd-mixture and the solution was stirred at rt. When full conversion was observed by TLC (2 h), the reaction mixture was concentrated under reduced pressure and purified by flash column chromatography (EtOAc:PE_{40-60} 1:19 to 22.5:77.5) to afford 1-(*tert*-butyl) 2-methyl (2*S*,5*S*)-5-((1-(tert-butoxycarbonyl)-1,2,3,6-tetrahydropyridin-4-yl)ethynyl)pyrrolidine-1,2-dicarboxylate **18** (65.4 mg, 76%) as a brown oil. A 1:1 mixture of rotamers was observed. R_f = 0.45 (EtOAc:PE_{40-60} (1:2), $KMnO_4$); IR (neat) ν (cm^{-1}): 2978, 2931, 1744, 1696, 1391, 1363, 1156, 1112; ^{1}H-NMR (400 MHz, $CDCl_3$): δ 5.95-5.94 (1H, m, H-11), 4.85 (1/2H, d, *J* 7.66 Hz, H-1), 4.72 (1/2H, d, *J* 7.66 Hz, H-1), 4.40 (1/2H, d, *J* 8.99 Hz, H-4), 4.30 (1/2H, d, *J* 8.99 Hz, H-4), 3.94-3.93 (2H, m, H-10), 3.72-3.71

(3H, m, H-16), 3.50-3.44 (2H, m, H-9), 2.50-2.35 (1H, m, H-3a), 2.29-2.20 (3H, m, H-2/H-8a), 2.02-1.93 (2H, m, H-3b/H-8b), 1.48-1.41 (18H, m, H-14/H-21); ^{13}C-NMR (100 MHz, $CDCl_3$): δ 173.3/173.0 (C-15), 154.7 (N**C**(O)C), 153.8/153.0 (N**C**(O)C), 130.3 (C-11), 119.1 (C-7), 88.6/88.2 (C-5), 82.0/81.8 (**C**(CH_3)$_3$), 80.4 (C-6), 79.9/79.8 (**C**(CH_3)$_3$), 59.0/58.4 (C-4), 52.2/52.0 (C-16), 49.4/49.2 (C-1), 43.8/43.1 (C-10), 40.6/39.3 (C-9), 32.0/31.3 (C-2), 29.3/29.2 (C-8), 28.5/28.3 (C-3), 28.4 (C(**C**H_3)$_3$), 28.2 (C(**C**H_3)$_3$); HRMS (ESI): *m/z* calcd for $C_{23}H_{34}N_2NaO_6$ $[M+H]^+$: 457.2315; found: 457.2306; $[\alpha]_D^{20}$ = -103.0 (c 0.4, $CHCl_3$).

(*S*)-5-(Hydroxymethyl)-1-(4-methoxyphenyl)pyrrolidin-2-one (S16)

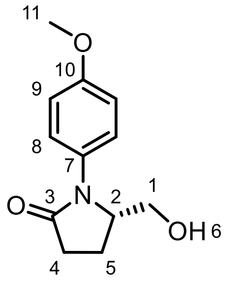

A microwave vial (2-5 mL) containing CuI (10.6 mg, 0.056 mmol, 5 mol%)**,** (*S*)-5-(hydroxymethyl)pyrrolidin-2-one (239.8 mg, 1.67 mmol, 1.5 equiv.) and Cs_2CO_3 (724.0 mg, 2.22 mmol, 2.0 equiv.) was evacuated and a nitrogen atmosphere was established. *N*,*N*'-Dimethylethylenediamine (12 µL, 0.11 mmol, 10 mol%), 4-iodoanisole (260.0 mg, 1.11 mmol) and DMF (1.1 mL) were added and the vial was capped. The resulting blue reaction mixture was stirred 24 h at 110 °C. The dark brown reaction mixture was filtered through a silica pad and eluted with a CH_2Cl_2:MeOH (9:1) solution. The filtrate was concentrated under reduced pressure and the crude product was purified by flash column chromatography (CH_2Cl_2:MeOH 99.5:0.5 to 23:2) to afford (*S*)-5-(hydroxymethyl)-1-(4-methoxyphenyl)pyrrolidin-2-one **S16** (219.3 mg, 89%) as a brownish thick oil. R_f = 0.35 (CH_2Cl_2:MeOH (9:1), UV); IR (neat) ν (cm^{-1}): 3279, 2959, 2840, 1639, 1611, 1580, 1509, 1437, 1250; ^{1}H-NMR (400 MHz, $CDCl_3$): δ 7.28-7.25 (2H, m, H-8), 6.92-6.90 (2H, m, H-9), 4.19-4.13 (1H, m, H-2), 3.80 (3H, s, H-11), 3.71-3.65 (1H, m, H-1a), 3.60-3.56 (1H, m, H-1b), 2.67 (1H, ddd, *J* 17.06, 10.14, 7.16 Hz, H-4a), 2.51 (1H, ddd, *J* 17.06, 10.14, 6.06 Hz, H-4b), 2.33 (1H, m, H-5a), 2.17-2.09 (1H, m, H-5b), 1.74 (1H, t, *J* 5.61 Hz, H-6); ^{13}C-NMR (100 MHz, $CDCl_3$): δ 175.4 (C-3), 158.1 (C-10), 130.2 (C-7), 126.3 (C-8), 114.7 (C-9), 62.7 (C-1), 61.7 (C-2), 55.6 (C-11), 31.4 (C-4), 21.1 (C-5); HRMS (ESI): *m/z* calcd for $C_{12}H_{16}NO_3$ $[M+H]^+$: 222.1125; found: 222.1121; $[\alpha]_D^{20}$ = -38.8 (c 0.25, $CHCl_3$).

(*S*)-1',6',7',7a'-Tetrahydro-5'*H*-spiro[cyclohexane-1,3'-pyrrolo[1,2-*c*]oxazole]-2,5-diene-4,5'-dione (19)

To a solution of (*S*)-5-(hydroxymethyl)-1-(4-methoxyphenyl)pyrrolidin-2-one **S16** (50.0 mg, 0.23 mmol) in

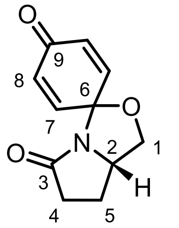

MeCN:H_2O (3:1, 9.1 mL) at -5 °C was added CAN (495.6 mg, 0.90 mmol, 4.0 equiv.). When full conversion was observed by TLC (5 min), the reaction mixture was diluted with EtOAc (5 mL) and quenched by the addition of sat. $NaHCO_3$ (5 mL, aq.). The aqueous layer was extracted with EtOAc (3 x 10 mL) and the combined organic layers were dried over Na_2SO_4, filtered and concentrated *in vacuo*. The crude product was purified by flash column chromatography (CH_2Cl_2:MeOH 100:0 to 24:1) to afford (*S*)-1',6',7',7a'-tetrahydro-5'*H*-spiro[cyclohexane-1,3'-pyrrolo[1,2-*c*]oxazole]-2,5-diene-4,5'-dione **19** (219.3 mg, 56%) as a brownish thick oil. R_f = 0.30 (CH_2Cl_2:MeOH (9:1), $KMnO_4$); ^{1}H-NMR (400 MHz, $CDCl_3$): δ 6.69 (1H, dd, *J* 10.06, 2.42 Hz, H-7), 6.52 (1H, dd, *J* 9.94, 2.42 Hz, H-7), 6.34 (1H, d, *J* 10.00 Hz, H-8), 6.21 (1H, d, *J* 9.88 Hz, H-8), 4.41 (1H, q, *J* 7.25 Hz, H-2), 4.30-4.26 (1H, m, H-1a), 3.58 (1H, t, *J* 8.62 Hz, H-1b), 2.88-2.79 (1H, m, H-4a), 2.61-2.55 (1H, m, H-4b), 2.39-2.32 (1H, m, H-5a), 1.99 (1H, m, H-5b); ^{13}C-NMR (100 MHz, $CDCl_3$): δ 185.0 (C-9), 172.2 (C-3), 142.9 (C-7), 142.5 (C-7), 130.4 (C-8), 129.2 (C-8), 82.9 (C-6), 71.1 (C-

1), 60.9 (C-2), 35.9 (C-4), 24.0 (C-5); HRMS (ESI): *m/z* calcd for $C_{11}H_{11}NNaO_3$ $[M+H]^+$: 228.0631; found: 228.0629; $[\alpha]_D^{20}$ = -106.6 (c 0.35, $CHCl_3$).

(*S*)-1-Allyl-5-((allyloxy)methyl)pyrrolidin-2-one (S17)

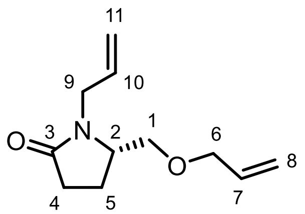

According to **general procedure C**, (*S*)-5-(hydroxymethyl)pyrrolidin-2-one (500.0 mg, 4.34 mmol) in DMF (15 mL) was added NaH (191.1 mg, 4.78 mmol, 1.1 equiv.) and allyl bromide (498 µL, 5.65 mmol, 1.3 equiv.). The reaction mixture was stirred for 75 min at rt. The title compound was purified by flash column chromatography (EtOAc:$PE_{40\text{-}60}$ 7:3 to 17:3) to afford (*S*)-1-allyl-5-((allyloxy)methyl)pyrrolidin-2-one **S17** (244.3 mg, 36%) as a clear oil. R_f = 0.20 (EtOAc:$PE_{40\text{-}60}$ (7:3), $KMnO_4$); IR (neat) ν (cm^{-1}): 2865, 3082, 1680, 1644, 1413; ^{1}H-NMR (400 MHz, $CDCl_3$): δ 5.91-5.81 (1H, m, H-7), 5.79-5.69 (1H, m, H-10), 5.28-5.22 (1H, dq, *J* 17.24, 1.61 Hz, H-8a), 5.19-5.13 (m, 3H, H-8b/H-11), 4.26 (1H, ddt, *J* 15.48, 4.9, 1.53 Hz, H-9a), 3.96 (2H, dt, *J* 5.53, 1.37 Hz, H-6), 3.78-3.73 (1H, m, H-2), 3.70-3.62 (1H, m, H-9b), 3.49-3.42 (2H, m, H-1), 2.52-2.44 (1H, m, H-4a), 2.32 (1H, m, H-4b), 2.16-2.06 (1H, m, H-5a), 1.91-1.83 (1H, m, H-5b); ^{13}C-NMR (100 MHz, $CDCl_3$): δ 175.3 (C-3), 134.4 (C-7), 133.1 (C-10), 117.5 (C-8), 117.3 (C-11), 72.3 (C-6), 71.3 (C-1), 57.4 (C-2), 43.9 (C-9), 30.3 (C-4), 22.0 (C-5); HRMS (ESI): *m/z* calcd for $C_{11}H_{18}NO_2$ $[M+H]^+$: 196.1332; found: 196.1336; $[\alpha]_D^{20}$ = 55.14 (c 0.37, $CHCl_3$).

(*S*,*Z*)-1,3,6,9,10,10a-hexahydro-8*H*-pyrrolo[2,1-*c*][1,4]oxazocin-8-one (20)

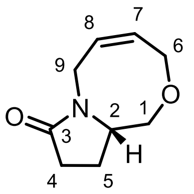

A solution of (*S*)-1-allyl-5-((allyloxy)methyl)pyrrolidin-2-one **S17** (50.0 mg, 0.26 mmol) in CH_2Cl_2 (60 mL) was degassed for 15 min, before Grubbs 2nd generation catalyst (21.7 mg, 0.026 mmol, 10 mol%) was added. The system was evacuated by three cycles of vacuum/nitrogen before a nitrogen atmosphere was established and the reaction mixture was refluxed. When full conversion was observed by TLC (1.5 h), the reaction mixture was concentrated under reduced pressure. The residual oil was filtered through silica gel using CH_2Cl_2 and increasing gradients of MeOH. The fractions with the crude product were concentrated and the crude product was purified by flash column chromatography (CH_2Cl_2:MeOH 100:0 to 98.6:1.4) to afford (*S*,*Z*)-1,3,6,9,10,10a-hexahydro-8*H*-pyrrolo[2,1-*c*][1,4]oxazocin-8-one **20** (24.6 mg, 57%) as an oil. R_f = 0.63 (EtOAc), $KMnO_4$); IR (neat) ν (cm^{-1}): 2927, 2868, 1667, 1418, 1121; ^{1}H-NMR (400 MHz, $CDCl_3$): δ 5.89-5.82 (1H, m, H-8), 5.58-5.53 (1H, m, H-7), 4.41-4.37 (1H, m, H-6a), 4.28-4.20 (2H, m, H-9a/H-6b), 3.95-3.82 (3H, m, H-2/H-1a/H-9b), 3.39-3.33 (1H, m, H-1b), 2.41 (2H, t, *J* 8.24 Hz, H-4), 2.11-2.02 (1H, m, H-5a), 1.49-1.40 (1H, m, H-5b); ^{13}C-NMR (100 MHz, $CDCl_3$): δ 174.0 (C-3), 131.5 (C-7), 124.9 (C-8), 75.3 (C-1), 70.7 (C-6), 58.0 (C-2), 38.8 (C-9), 30.7 (C-4), 20.0 (C-5); HRMS (ESI): *m/z* calcd for $C_9H_{14}NO_2$ $[M+H]^+$: 168.1019; found: 168.1014; $[\alpha]_D^{20}$ = -59.8 (c 0.4, $CHCl_3$).

Methyl 2-(2-cyanoethyl)-5-phenyl-3,4-dihydro-2*H*-pyrrole-2-carboxylate (S18)

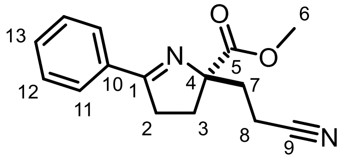

To a stirring mixture of acrylonitrile (171 µL, 2.61 mmol, 1.1 equiv.), $Cu(MeCN)_4BF_4$ (38.7 mg, 0.12 mmol, 5 mol%), PCy_3 (73.1 mg, 0.26 mmol, 11 mol%.) and Et_3N (198 µL, 1.42 mmol, 0.6 equiv.) in THF (1.4 mL) under nitrogen atmosphere was added methyl (*S*)-5-phenyl-3,4-dihydro-2*H*-pyrrole-2-carboxylate[12] (481.0 mg, 2.37 mmol) in THF (1.0 mL). When full conversion was observed by TLC (2 h), the reaction mixture was filtered through a pad of celite using EtOAc and concentrated under reduced pressure. The crude

product was purified by flash column chromatography (EtOAc:PE_{40-60} 3:7 to 2:3) to afford methyl 2-(2-cyanoethyl)-5-phenyl-3,4-dihydro-2*H*-pyrrole-2-carboxylate **S18** (434.5 mg, 72%) as a green oil. R_f = 0.60 (EtOAc:PE_{40-60} (1:1), UV); IR (neat) ν (cm^{-1}): 2953, 2245, 1728, 1616, 1200; ^{1}H-NMR (400 MHz, $CDCl_3$): δ 7.89-7.86 (2H, m, H-11), 7.50-7.41 (3H, m, H-12/H-13), 3.75 (3H, s, H-6), 3.22-3.06 (2H, m, H-2), 2.70-2.62 (1H, m, H-8a), 2.59-2.43 (3H, m, H-3a/H-7a/H-8b), 2.10-1.95 (2H, m, H-3b/H-7b); ^{13}C-NMR (100 MHz, $CDCl_3$): δ 176.1 (C-1), 173.7 (C-5), 133.7 (C-10), 131.5 (C-13), 128.7 (C-11), 128.3 (C-12), 119.9 (C-9), 81.7 (C-4), 52.8 (C-6), 35.8 (C-2), 34.7 (C-7), 33.0 (C-3), 13.3 (C-8); HRMS (ESI): *m/z* calcd for $C_{15}H_{17}N_2O_2$ $[M+H]^+$: 257.1290; found: 257.1289.

2-Phenyl-1,7-diazaspiro[4.5]dec-1-en-6-one (21)

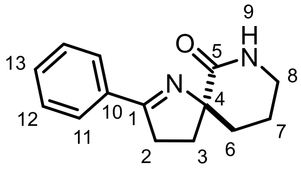

According to the **General procedure A**, methyl 2-(2-cyanoethyl)-5-phenyl-3,4-dihydro-2*H*-pyrrole-2-carboxylate **S18** (50.4 mg, 0.20 mmol) in MeOH (2.0 mL) and Raney-Nickel (2.0 mL, 50% in H_2O) was vigorously stirred overnight. The crude product was purified by flash column chromatography (CH_2Cl_2:MeOH 99:1 to 93:7) to afford 2-phenyl-1,7-diazaspiro[4.5]dec-1-en-6-one **21** (30.0 mg, 66%) as an off-white amorphous solid. R_f = 0.26 (CH_2Cl_2:MeOH (9:1), UV); IR (neat) ν (cm^{-1}): 3192, 2945, 2859, 1668, 1615; ^{1}H-NMR (400 MHz, $CDCl_3$): δ 7.88-7.86 (2H, m, H-11), 7.44-7.36 (3H, m, H-12/H-13), 6.02 (1H, s, H-9), 3.51-3.46 (1H, m, H-8a), 3.42-3.36 (1H, m, H-8b), 3.28 (1H, dddd, *J* 16.97, 10.03, 6.97 Hz, H-2a), 3.10-3.02 (1H, m, H-2b), 2.65-2.58 (1H, m, H-3a), 2.32-2.23 (1H, m, H-7a), 2.15-2.09 (1H, m, H-6a), 1.89-1.76 (3H, m, H-6b/H-7b/H-3b); ^{13}C-NMR (100 MHz, $CDCl_3$): δ 174.5 (C-1), 173.9 (C-5), 134.5 (C-10), 130.7 (C-13), 128.4 (C-12), 128.2 (C-11), 79.4 (C-4), 43.1 (C-8), 36.2 (C-2), 35.3 (C-6), 34.1 (C-3), 20.4 (C-7); HRMS (ESI): *m/z* calcd for $C_{14}H_{17}N_2O$ $[M+H]^+$:229.1341; found: 229.1343.

Methyl 2-(2-nitro-1-phenylethyl)-5-phenyl-3,4-dihydro-2*H*-pyrrole-2-carboxylate (S19)

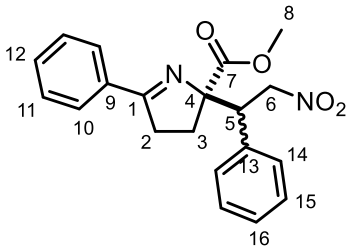

To a stirring mixture of *trans*-β-nitrostyrene (176.0 mg, 1.18 mmol, 1.2 equiv.), $Cu(MeCN)_4BF_4$ (16.1 mg, 0.051 mmol, 5 mol%), PCy_3 (30.4 mg, 0.011 mmol, 11 mol%) and Et_3N (82 μL, 0.59 mmol, 0.6 equiv.) in THF (0.5 mL) under nitrogen atmosphere was added methyl (*S*)-5-phenyl-3,4-dihydro-2*H*-pyrrole-2-carboxylate[12] (200.0 mg, 0.98 mmol) in THF (0.5 mL) and the reaction mixture was stirred overnight. The reaction mixture was filtered through a pad of celite using EtOAc and concentrated under reduced pressure. The crude product was purified by flash column chromatography (EtOAc:PE_{40-60} 1:9 to 7:13) to afford methyl 2-(2-nitro-1-phenylethyl)-5-phenyl-3,4-dihydro-2*H*-pyrrole-2-carboxylate **S19** (262.0 mg, 75%) as a greenish foam. *A mixture of two diastereoisomers was inseparable by column chromatography*. R_f = 0.57 (EtOAc:PE_{40-60} (1:1), UV); IR (neat) ν (cm^{-1}): 3031, 2953, 1726, 1616, 1548, 1378, 1236, 1164; ^{1}H-NMR (400 MHz, $CDCl_3$): δ 7.84-7.81 (2H, m, H-10), 7.51-7.41 (3H, m, H-11/H-12), 7.20-7.18 (5H, m, H-14/H-15/H-16), 5.14 (1H, dd, *J* 13.4, 10.9 Hz, H-6a), 5.00 (1H, dd, *J* 13.4, 4.0 Hz, H-6b), 4.39 (1H, dd, *J* 10.9, 4.0 Hz, H-5), 3.81 (3H, s, H-8), 2.85-2.77 (1H, m, H-2a), 2.23-2.15 (1H, m, H-3a), 2.06-1.98 (2H, m, H-2b/H-3b); ^{13}C-NMR (100 MHz, $CDCl_3$): δ 177.7 (C-1), 174.2 (C-7), 135.3 (C-13), 133.5 (C-9), 131.5 (C-12), 129.9 (C-Ar), 128.7 (C-Ar), 128.5 (C-10), 128.22 (C-Ar), 128.15 (C-16), 84.9 (C-4), 77.7 (C-6),

53.1 (C-8), 50.3 (C-5), 35.4 (C-2), 31.6 (C-3); HRMS (ESI): *m/z* calcd for $C_{20}H_{21}N_2O_4$ $[M+H]^+$: 353.1501; found: 353.1486.

2,9-Diphenyl-1,7-diazaspiro[4.4]non-1-en-6-one (22)

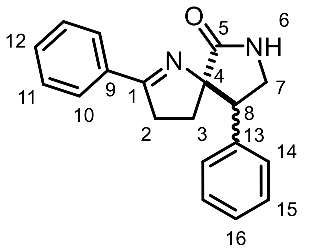

According to the **General procedure A**, the diastereomeric mixture of methyl 2-(2-nitro-1-phenylethyl)-5-phenyl-3,4-dihydro-2*H*-pyrrole-2-carboxylate **S19** (82.6 mg, 0.23 mmol) in MeOH (2.4 mL) and Raney-Nickel (2.4 mL, 50% in H_2O) was vigorously stirred overnight. The crude product was purified by flash column chromatography (EtOAc:PE_{40-60} 9:11 to 17:3) to afford 2,9-diphenyl-1,7-diazaspiro[4.4]non-1-en-6-one **22** (37.3 mg, 55%) as a white amorphous solid. *A mixture of two diastereoisomers was inseparable by column chromatography*. R_f = 0.18 (EtOAc:PE_{40-60} (4:1), UV); IR (neat) ν (cm^{-1}): 3195, 2926, 2855, 1694, 1605; ^{1}H-NMR (400 MHz, $CDCl_3$): δ 7.74-7.72 (2H, m, H-10), 7.40-7.32 (5H, m, H-Ar), 7.25-7.16 (3H, m, H-Ar), 6.46 (1H, br s, H-6), 4.07-4.02 (1H, m, H-7a), 3.67-3.63 (1H, m, H-7b), 3.52-3.48 (1H, m, H-8), 3.17-3.09 (1H, m, H-2a), 2.67-2.60 (1H, m, H-3a), 2.56-2.48 (1H, m, H-2b), 1.88-1.81 (1H, m, H-3b); ^{13}C-NMR (100 MHz, $CDCl_3$): δ 177.3 (C-5), 175.0 (C-1), 135.9 (C-13), 134.3 (C-9), 130.7 (C-12), 130.2 (C-Ar), 128.4 (C-Ar), 128.1 (C-Ar), 128.0 (C-Ar), 127.6 (C-Ar), 84.0 (C-4), 54.0 (C-8), 46.3 (C-7), 36.1 (C-2), 28.0 (C-3); HRMS (ESI): *m/z* calcd for $C_{19}H_{19}N_2O$ $[M+H]^+$: 291.1497; found: 291.1483.

Methyl 2-(3-methoxy-3-oxopropyl)-5-phenyl-3,4-dihydro-2*H*-pyrrole-2-carboxylate (S20)

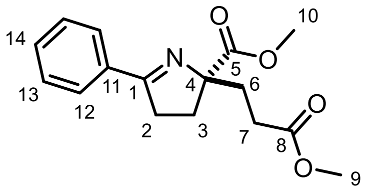

To a stirring mixture of methyl acrylate (106 µL, 1.18 mmol, 1.2 equiv.), $Cu(MeCN)_4BF_4$ (16.1 mg, 0.05 mmol, 5 mol%), PCy_3 (30.4 mg, 0.11 mmol, 11 mol%) and Et_3N (82.4 µL, 0.59 mmol, 0.6 equiv.) in THF (0.5 mL) under nitrogen atmosphere was added methyl (*S*)-5-phenyl-3,4-dihydro-2*H*-pyrrole-2-carboxylate[12] (200.0 mg, 0.98 mmol) in THF (0.5 mL) and the reaction mixture was stirred overnight. The reaction mixture was filtered through a pad of celite using EtOAc and concentrated under reduced pressure. The crude product was purified by flash column chromatography (EtOAc:PE_{40-60} 3:17 to 2:3) to afford methyl 2-(3-methoxy-3-oxopropyl)-5-phenyl-3,4-dihydro-2*H*-pyrrole-2-carboxylate **S20** (190.0 mg, 67%) as a clear oil. R_f = 0.50 (EtOAc:PE_{40-60} (1:1), UV); IR (neat) ν (cm^{-1}): 2952, 1728, 1615, 1194, 1167; ^{1}H-NMR (400 MHz, $CDCl_3$): δ 7.88-7.85 (2H, m, H-12), 7.47-7.38 (3H, m, H-13/H-14), 3.74 (3H, s, H-10), 3.64 (3H, s, H-9), 3.17-3.00 (2H, m, H-2), 2.59-2.51 (1H, m, H-7a), 2.47-2.36 (3H, m, H-3a/H-6a/H-7b), 2.20-2.12 (1H, m, H-6b), 1.99 (1H, dddd, *J* 13.38, 9.60, 6.54 Hz, H-3b); ^{13}C-NMR (100 MHz, $CDCl_3$): δ 174.9 (C-1), 174.6 (C-5), 173.9 (C-8), 134.0 (C-11), 131.1 (C-14), 128.6 (C-13), 128.2 (C-12), 82.5 (C-4), 52.6 (C-10), 51.8 (C-9), 35.6 (C-2), 34.0 (C-6), 32.4 (C-3), 29.8 (C-7); HRMS (ESI): *m/z* calcd for $C_{16}H_{20}NO_4$ $[M+H]^+$: 290.1392; found: 290.1392.

Reduction of methyl 2-(3-methoxy-3-oxopropyl)-5-phenyl-3,4-dihydro-2*H*-pyrrole-2-carboxylate

To a cooled solution of methyl 2-(3-methoxy-3-oxopropyl)-5-phenyl-3,4-dihydro-2*H*-pyrrole-2-carboxylate **S20** (75.0 mg, 0.26 mmol) in toluene (2.6 mL) under nitrogen atmosphere was added AcOH (178 µL, 3.11 mmol, 12.0 equiv.) and $NaBH_3CN$ (195.5 mg, 3.11 mmol, 12.0 equiv.) and the mixture was stirred overnight. The following day, the reaction mixture was quenched by the addition of H_2O (5 mL) and extracted with EtOAc (3 x 30 mL).

The combined organic layers were dried over Na_2SO_4, filtered and concentrated *in vacuo*. The crude products were purified by flash column chromatography (EtOAc:PE_{40-60} 1:9 to 7:3).

Methyl 3-oxo-5-phenyltetrahydro-1*H*-pyrrolizine-7a(5*H*)-carboxylate (23a)

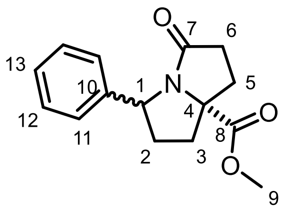

Off-white amorphous solid (8.3 mg, 12%). R_f = 0.14 (EtOAc:PE_{40-60} (1:1), $KMnO_4$); IR (neat) ν (cm^{-1}): 2956, 1726, 1686, 1603, 1371, 1176; ^{1}H-NMR (400 MHz, $CDCl_3$): δ 7.30-7.29 (4H, m, H-11/H-12), 7.23-7.19 (1H, m, H-13), 4.97 (1H, t, *J* 8.32 Hz, H-1), 3.73 (3H, s, H-9), 3.07-2.98 (1H, m, H-5a), 2.71-2.62 (2H, m, H-2), 2.54-2.48 (2H, m, H-6a/H-5b), 2.21-2.12 (1H, m, H-6b), 2.10-2.00 (1H, m, H-3a), 1.79-1.71 (1H, m, H-3b); ^{13}C-NMR (100 MHz, $CDCl_3$): δ 176.7 (C-7), 174.3 (C-8), 141.6 (C-10), 128.6 (C-Ar), 127.1 (C-13), 125.8 (C-Ar), 74.3 (C-4), 59.1 (C-1), 52.7 (C-9), 36.8 (C-3), 36.7 (C-2), 34.6 (C-5), 33.2 (C-6); HRMS (ESI): *m/z* calcd for $C_{15}H_{18}NO_3$ $[M+H]^+$: 260.1287; found: 260.1277.

Methyl 2-(3-methoxy-3-oxopropyl)-5-phenylpyrrolidine-2-carboxylate (23b)

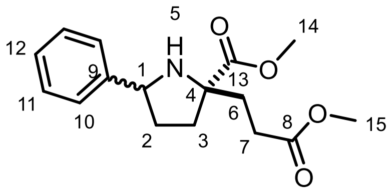

A clear oil (30.0 mg, 39%). R_f = 0.74 (EtOAc:PE_{40-60} (1:1), UV); IR (neat) ν (cm^{-1}): 3348, 2952, 1726, 1602, 1194, 1370,1167; ^{1}H-NMR (400 MHz, $CDCl_3$): δ 7.40-7.38 (2H, m, H-10), 7.30 (2H, t, *J* 7.43 Hz, H-11), 7.24-7.20 (1H, m, H-12), 4.29-4.25 (1H, m, H-1), 3.76 (3H, s, H-14), 3.67 (3H, s, H-15), 2.73 (1H, br s, H-5), 2.53-2.45 (1H, m, H-7a), 2.41-2.33 (1H, m, H-7b), 2.26-2.19 (2H, m, H-2a/-H6a), 2.14-2.01 (2H, m, H-3a/H-6b), 1.98-1.92 (1H, m, H-2b), 1.72-1.63 (1H, m, H-3b); ^{13}C-NMR (100 MHz, $CDCl_3$): δ 177.9 (C-13), 174.2 (C-8), 144.7 (C-9), 128.3 (C-11), 127.0 (C-12), 126.7 (C-10), 68.0 (C-4), 62.2 (C-1), 52.6 (C-14), 51.8 (C-15), 35.3 (C-2), 35.2 (C-6), 34.8 (C-3), 29.8 (C-7); HRMS (ESI): *m/z* calcd for $C_{16}H_{22}NO_4$ $[M+H]^+$: 292.1549; found: 292.1537.

Methyl 2-(1-(*tert*-butoxycarbonyl)-3-(2-ethoxy-2-oxoethyl)azetidin-3-yl)-5-phenyl-3,4-dihydro-2H-pyrrole-2-carboxylate (24)

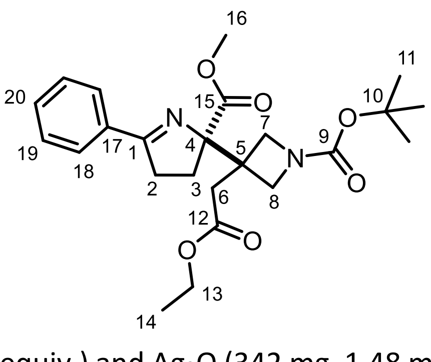

To a stirring solution of methyl (*S*)-5-phenyl-3,4-dihydro-2*H*-pyrrole-2-carboxylate[12] (300.0 mg, 1.48 mmol) and *tert*-butyl 3-(2-ethoxy-2-oxoethylidene)azetidine-1-carboxylate[13] (356.2 mg, 1.48 mmol, 1.0 equiv.) in toluene (59 mL) was added DBU (221 µL, 1.48 mmol, 1.0 equiv.) and Ag_2O (34.2 mg, 0.15 mmol, 10 mol%) under a nitrogen atmosphere. The reaction mixture was stirred for 64 h. Incomplete conversion was observed by TLC and therefore additional *tert*-butyl 3-(2-ethoxy-2-oxoethylidene)azetidine-1-carboxylate (356.2 mg, 1.48 mmol, 1.0 equiv.), DBU (331 µL, 2.21 mmol, 1.5 equiv.) and Ag_2O (342 mg, 1.48 mmol, 1.0 equiv.) were added and the reaction mixture was stirred overnight to achieve full conversion. The reaction mixture was quenched by the addition of H_2O (20 mL) and extracted with EtOAc (3 x 100 mL). Combined organic layers were dried over Na_2SO_4, filtered and concentrated *in vacuo*. The crude product was purified by flash column chromatography (EtOAc:PE_{40-60} 5:1) to afford methyl 2-(1-(*tert*-butoxycarbonyl)-3-(2-ethoxy-2-oxoethyl)azetidin-3-yl)-5-phenyl-3,4-dihydro-2H-pyr-role-2-carboxy-late **24** (481.0 mg, 73%) as a clear oil. R_f = 0.58 (EtOAc:PE_{40-60} (1:1), UV); IR (neat) ν (cm^{-1}): 2978, 2959, 1729, 1695, 1615,

1576, 1390, 1365, 1148; ^{1}H-NMR (400 MHz, $CDCl_3$): δ 7.86 (2H, d, *J* 7.24 Hz, H-18), 7.48-7.38 (3H, m, H-19/H-20), 4.27-4.14 (3H, m, H-7a/H-13), 4.02-3.87 (2H, m, H-7b/H-8a), 3.79-3.75 (1H, m, H-8b), 3.69 (3H, s, H-16), 3.24-3.10 (2H, m, H-6), 2.90-2.83 (1H, m, H-2a), 2.66 (1H, d, *J* 14.3 Hz, H-2b), 2.56-2.50 (1H, m, H-3a), 2.01 (1H, m, H-3b), 1.43 (9H, s, H-11), 1.27 (3H, t, *J* 7.1 Hz, H-14); ^{13}C-NMR (100 MHz, $CDCl_3$): δ 176.5 (C-1), 173.0 (C-15), 171.2 (C-12), 156.5 (C-9), 133.8 (C-17), 131.3 (C-20), 128.6 (C-19), 128.4 (C-18), 86.9 (C-4), 79.4 (C-10), 60.8 (C-13), 55.8/55.0 (C-7/C-8), 54.5/53.8 (C-7/C-8), 52.6 (C-16), 41.8 (C-5), 39.7 (C-2), 36.5 (C-6), 28.9 (C-3), 28.5 (C-11), 14.3 (C-14); HRMS (ESI): *m/z* calcd for $C_{24}H_{33}N_2O_6$ [M+H]$^+$: 445.2333; found: 445.2325.

Methyl (2*S*)-6-(benzyloxy)-7-oxo-5-phenyl-1-azabicyclo[3.2.0]heptane-2-carboxylate (25)

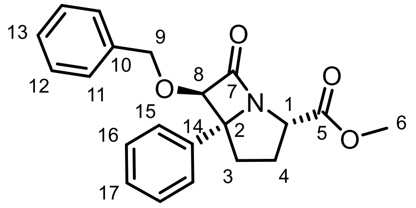

To a cold solution of methyl (*S*)-5-phenyl-3,4-dihydro-2*H*-pyrrole-2-carboxylate[12] (50.0 mg, 0.25 mmol) and Et_3N (134 µL, 0.96 mmol, 3.9 equiv.) in CH_2Cl_2 (1.2 mL) was dropwise added a solution of benzyloxyacetyl chloride (50.5 µL, 0.32 mmol, 1.3 equiv.) in CH_2Cl_2 (0.6 mL). The resulting mixture was stirred overnight at rt. The reaction was quenched by the addition of H_2O (10 mL) and extracted with CH_2Cl_2 (3 x 10 mL). The combined organic layers were dried over $MgSO_4$, filtered and concentrated *in vacuo*. The crude was crystallised by dissolving the residues in a minimum of hot acetone and then pouring it into a stirring solution of PE_{40-60}. This afford methyl (2*S*)-6-(benzyloxy)-7-oxo-5-phenyl-1-azabicyclo[3.2.0]heptane-2-carboxylate **25** (60.5 mg, 70%) as grey solid. R_f = 0.14 (EtOAc:PE_{40-60} (1:3), UV); IR (neat) ν (cm^{-1}): 3032, 2952, 1768, 1735, 1453, 1162, 1015; ^{1}H-NMR (400 MHz, $CDCl_3$): δ 7.58 (2H, d, *J* 7.64 Hz, H-15), 7.42 (2H, t, *J* 7.38 Hz, H-16), 7.36-7.33 (1H, m, H-17), 7.22-7.21 (3H, m, H-12/H-13), 6.91-6.87 (2H, m, H-11), 4.65 (1H, s, H-8), 4.54 (1H, t, *J* 8.12 Hz, H-1), 4.39 (1H, d, *J* 11.02 Hz, H-9a), 4.28 (1H, d, *J* 11.02 Hz, H-9b), 3.76 (3H, s, H-6), 2.55-2.48 (2H, m, H-3a/H-4a), 2.25-2.15 (1H, m, H-4b), 1.96 (1H, dt, *J* 12.21, 7.25 Hz, H-3b); ^{13}C-NMR (100 MHz, $CDCl_3$): δ 173.6 (C-7), 171.3 (C-5), 137.8 (C-14), 136.6 (C-10), 128.43 (C-16), 128.39 (C-12), 128.1 (C-11), 128.0 (C-13), 127.8 (C-17), 127.4 (C-15), 87.9 (C-8), 74.2 (C-2), 72.0 (C-9), 59.4 (C-1), 52.6 (C-6), 37.2 (C-3), 33.6 (C-4); HRMS (ESI): *m/z* calcd for $C_{21}H_{22}NO_4$ [M+H]$^+$: 352.1543; found: 352.1533; $[\alpha]_D^{20}$ = -156.7 (c 0.30, $CHCl_3$).

NMR spectra

Methyl (*S*)-1-((1*R*,6*R*)-6-cyanocyclohex-2-en-1-yl)-5-oxopyrrolidine-2-carboxylate (S1)

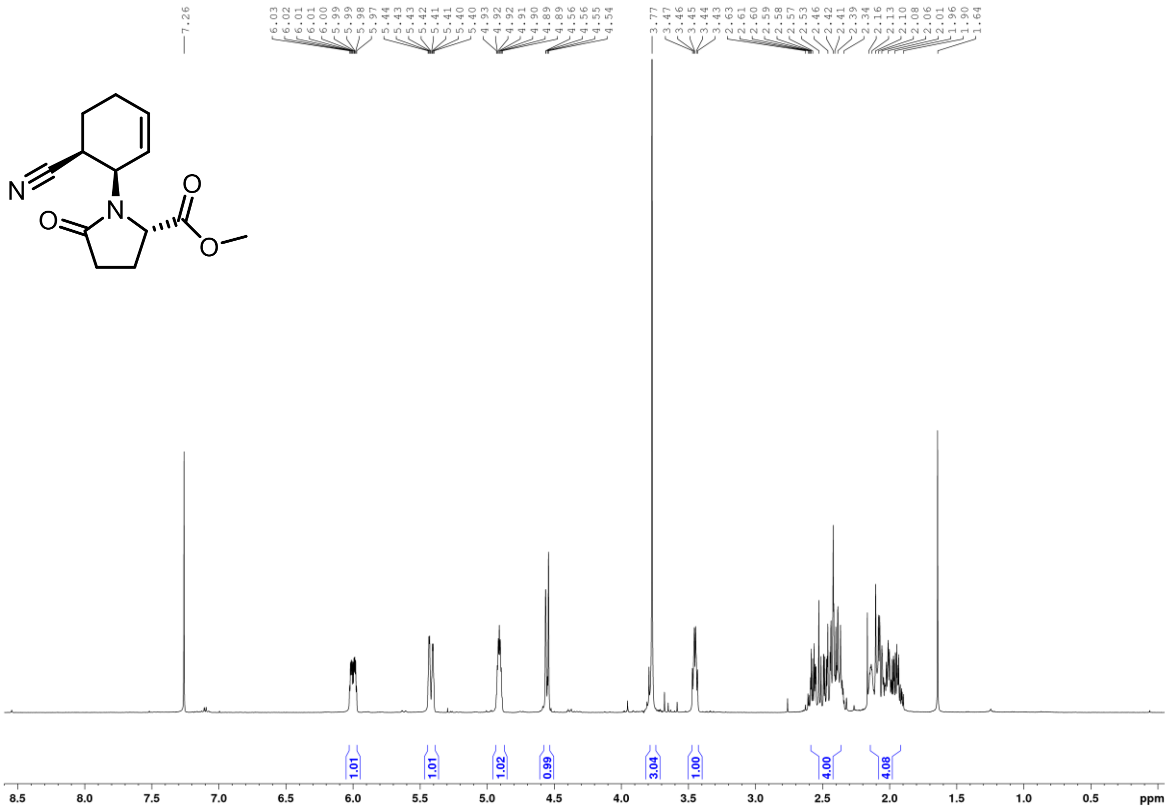

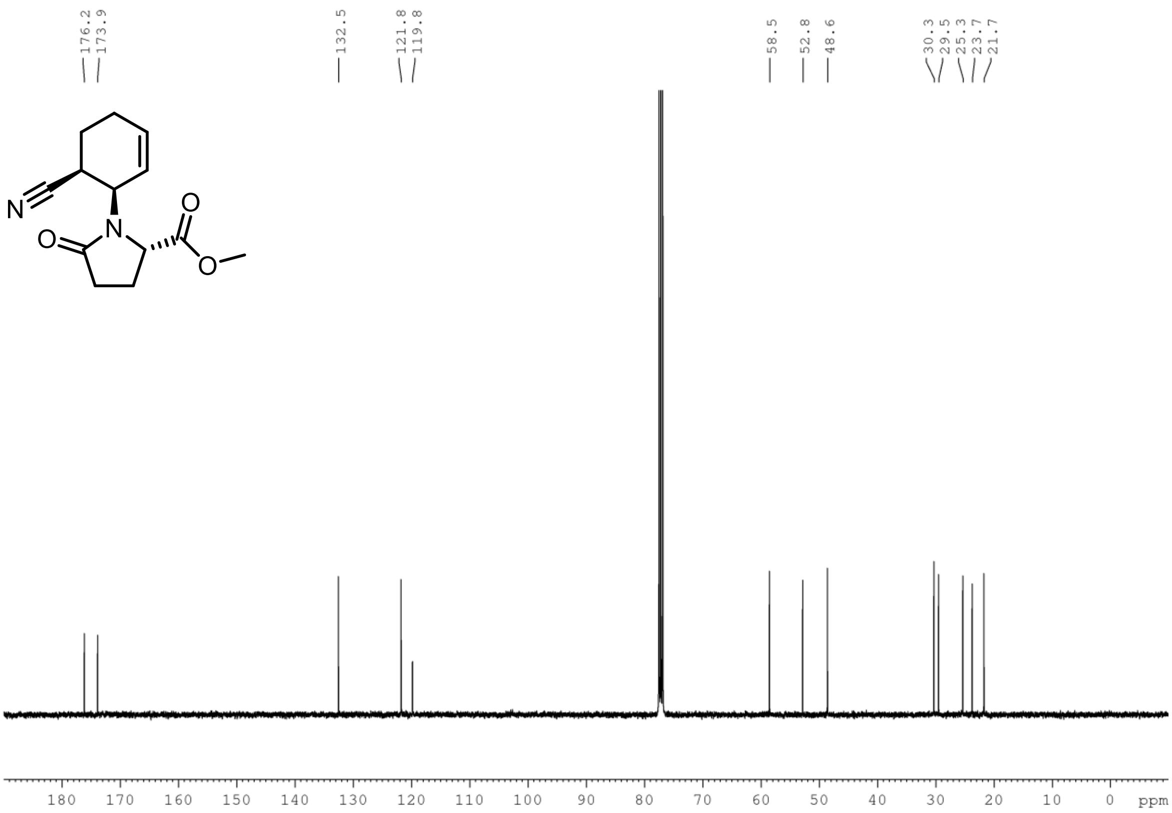

(3a*S*,6a*S*,10a*S*)-3,3a,5,6,6a,7,8,10a-Octahydro-1*H*-benzo[*f*]pyrrolo[1,2-*a*][1,4]diazepine-1,4(2*H*)-dione (6a)

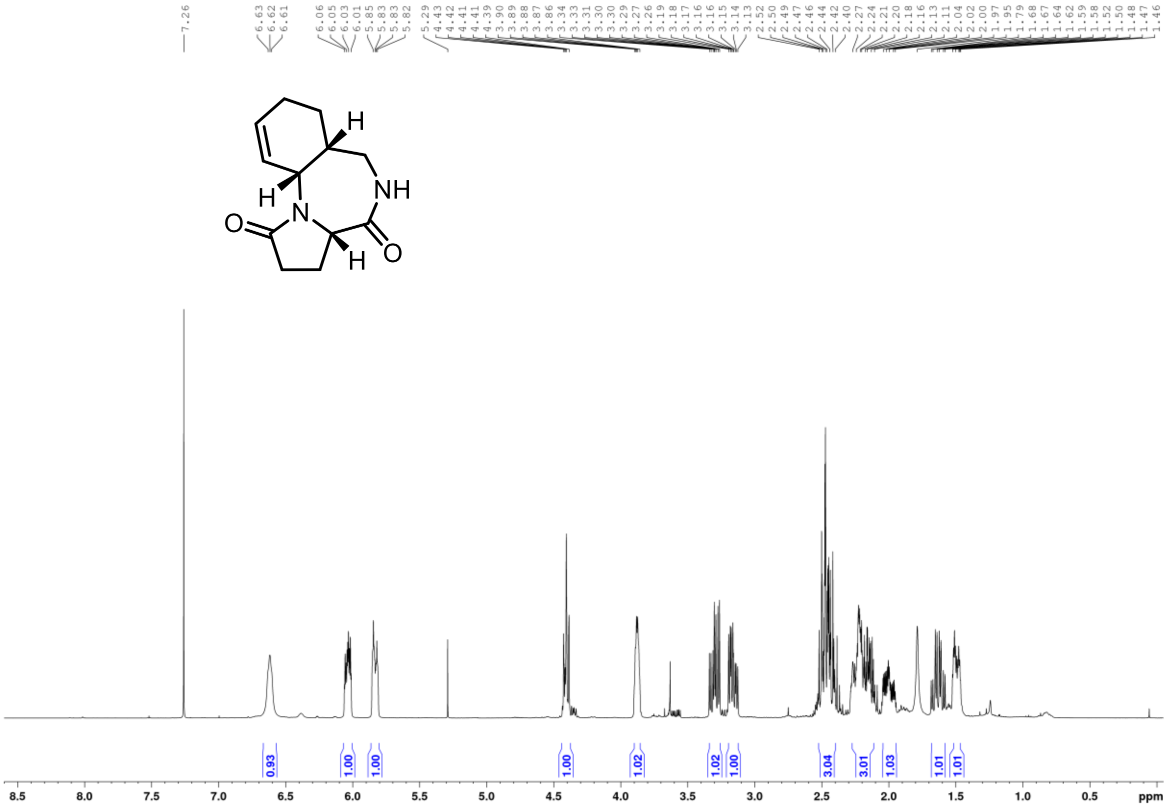

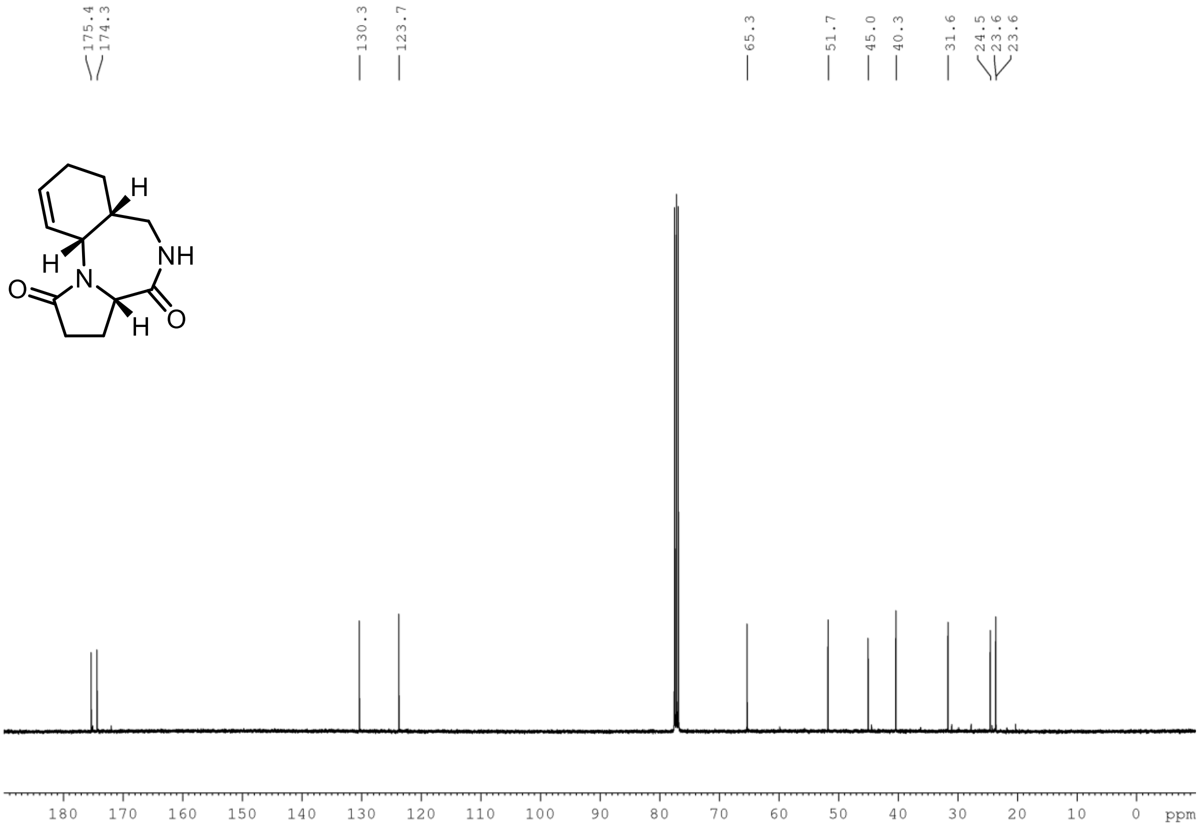

(3a*S*,6a*S*,10a*S*)-Decahydro-1*H*-benzo[*f*]pyrrolo[1,2-*a*][1,4]diazepine-1,4(2*H*)-dione (6b)

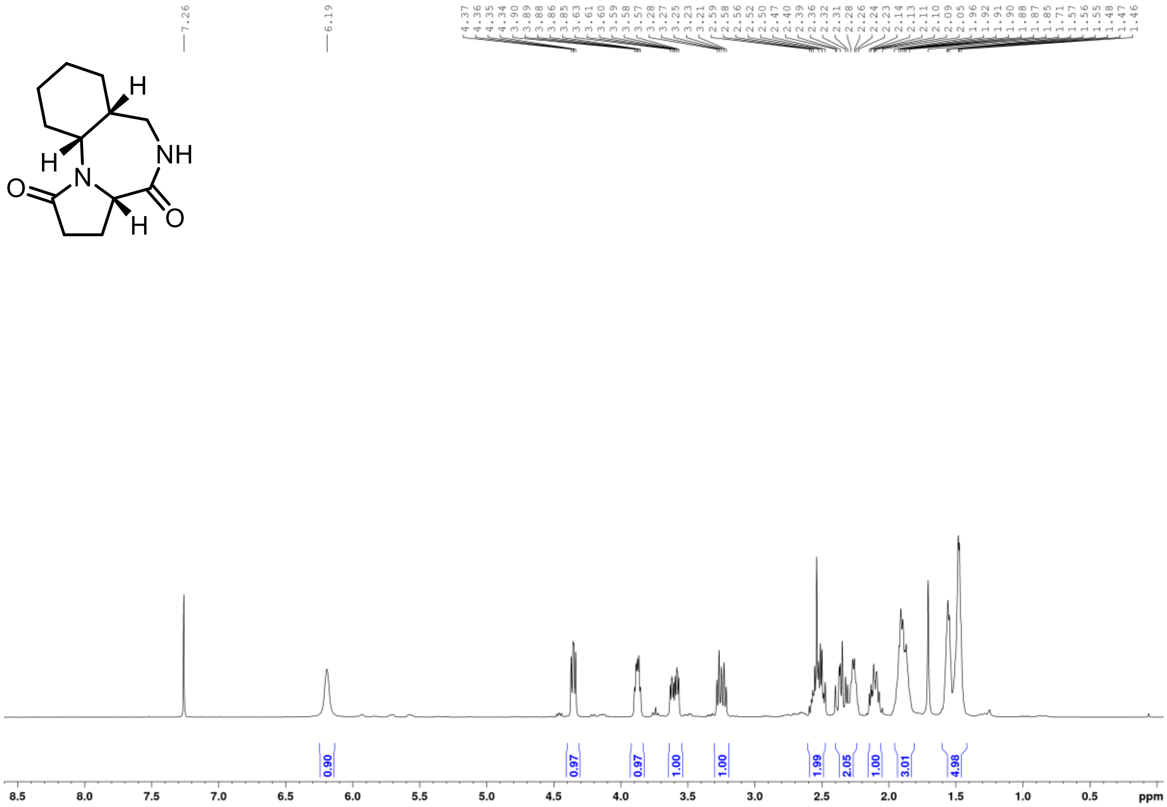

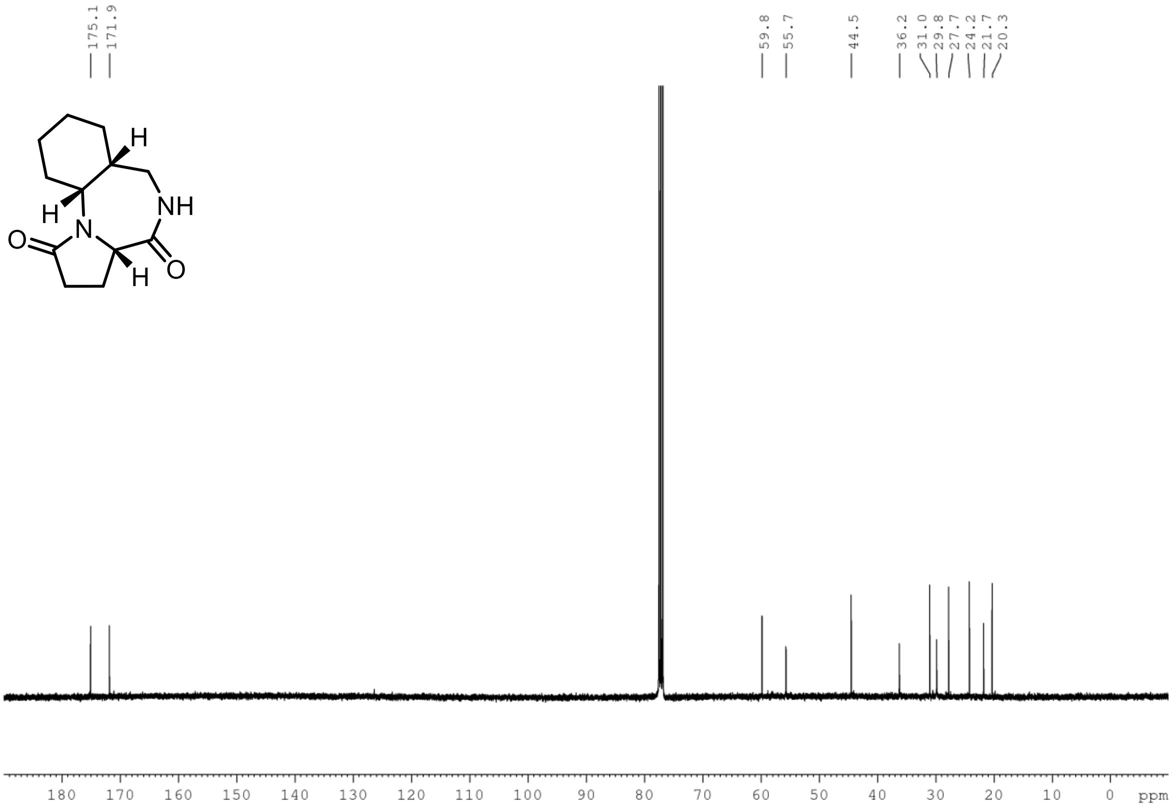

Methyl (*S*)-1-((1*R*,2*R*,3*R*)-2-nitro-1,2,3,6-tetrahydro-[1,1'-biphenyl]-3-yl)-5-oxopyrrolidine-2-carboxylate (S2)

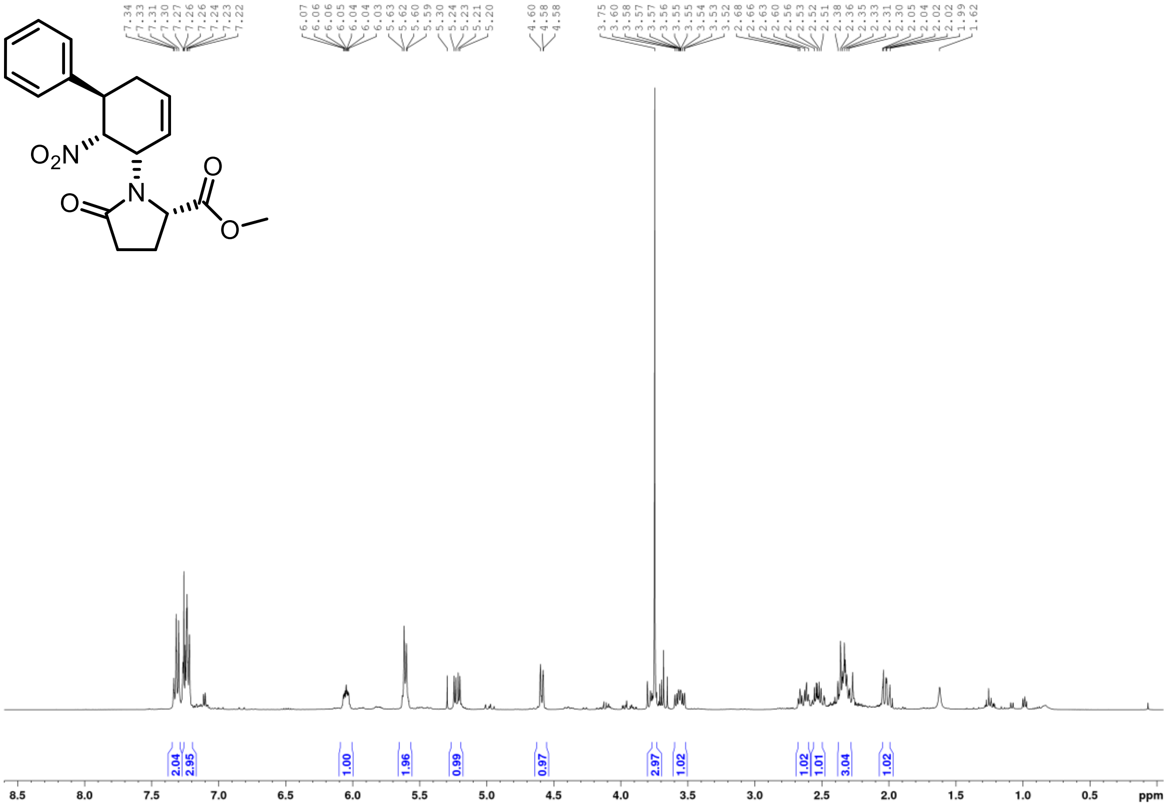

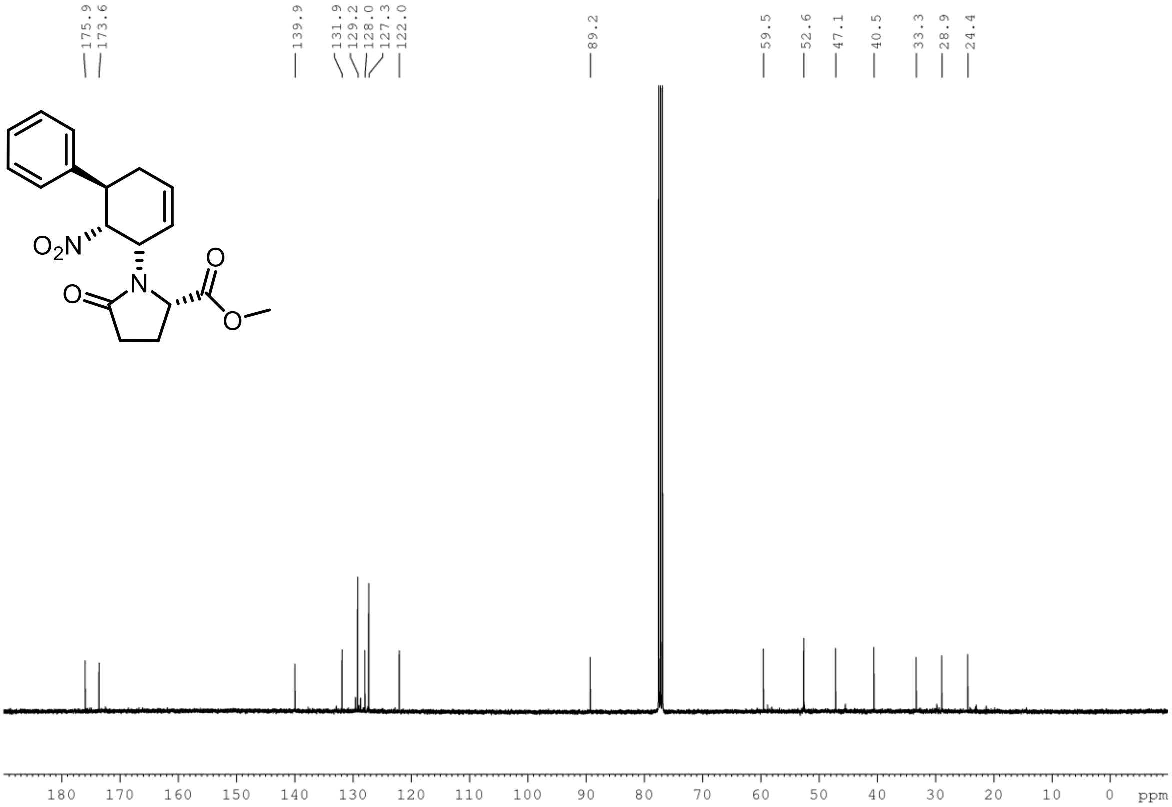

Methyl (*S*)-1-((1*R*,2*R*,3*R*)-2-amino-1,2,3,6-tetrahydro-[1,1'-biphenyl]-3-yl)-5-oxopyrrolidine-2-carboxylate (S3)

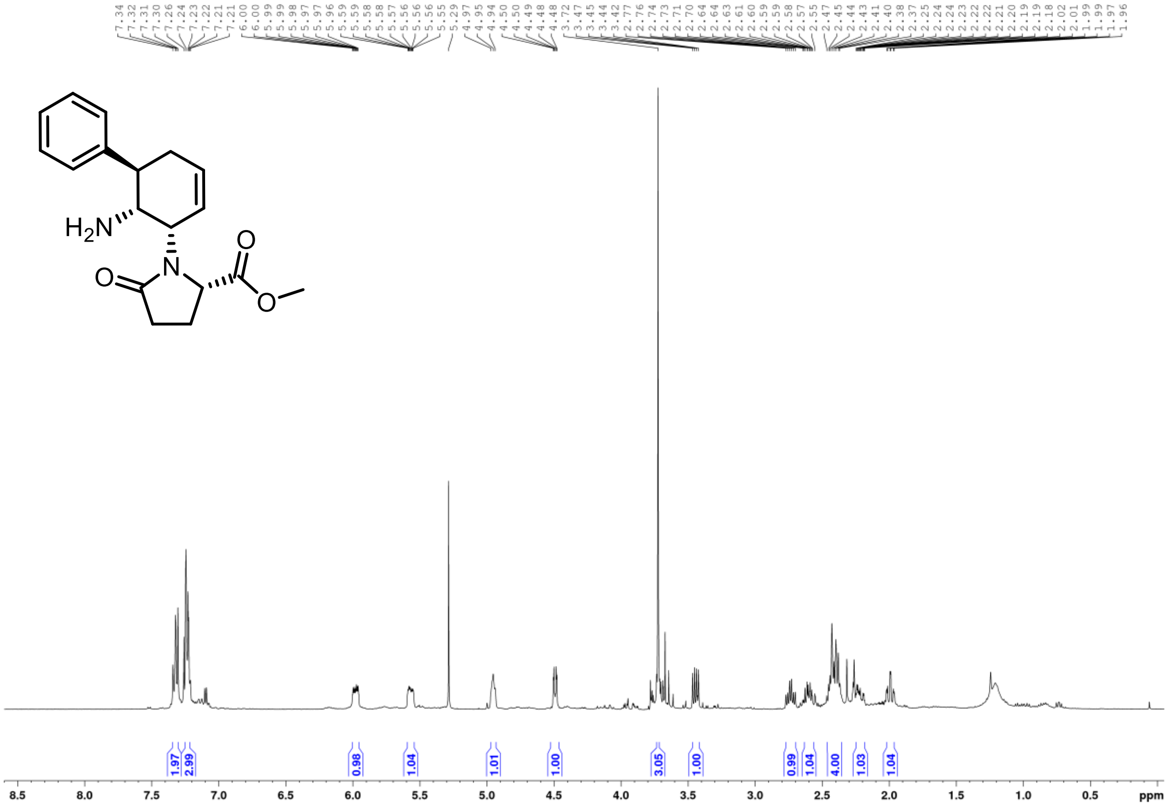

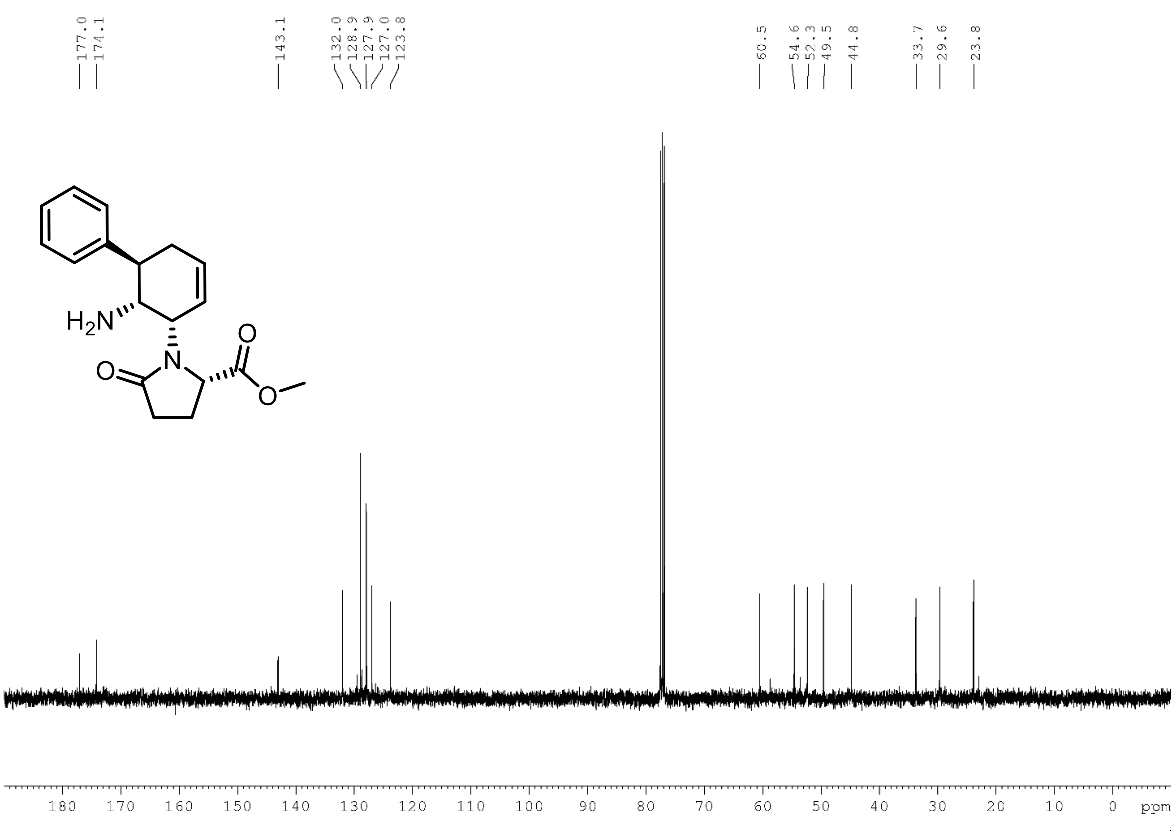

(3a*S*,5a*R*,6*R*,9a*S*)-6-Phenyl-3,3a,5a,6,7,9a-hexahydropyrrolo[1,2-*a*]quinoxaline-1,4(2*H*,5*H*)-dione (7)

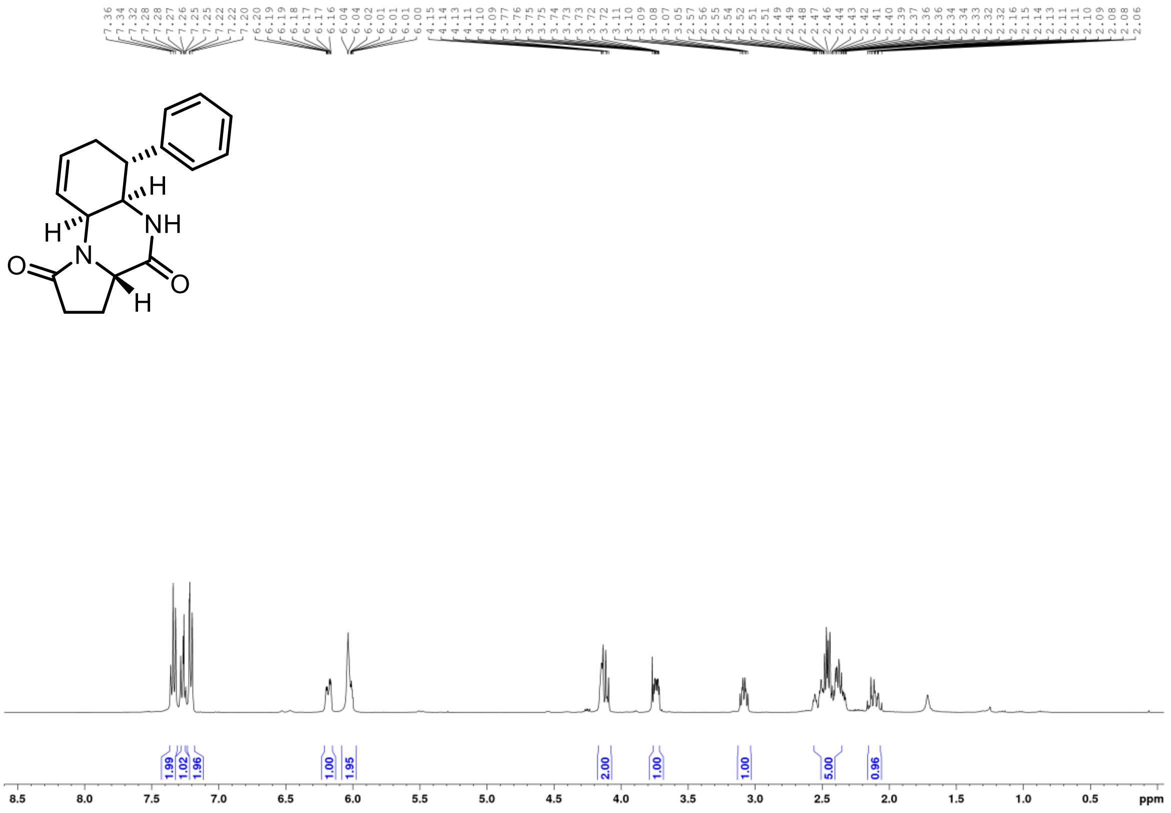

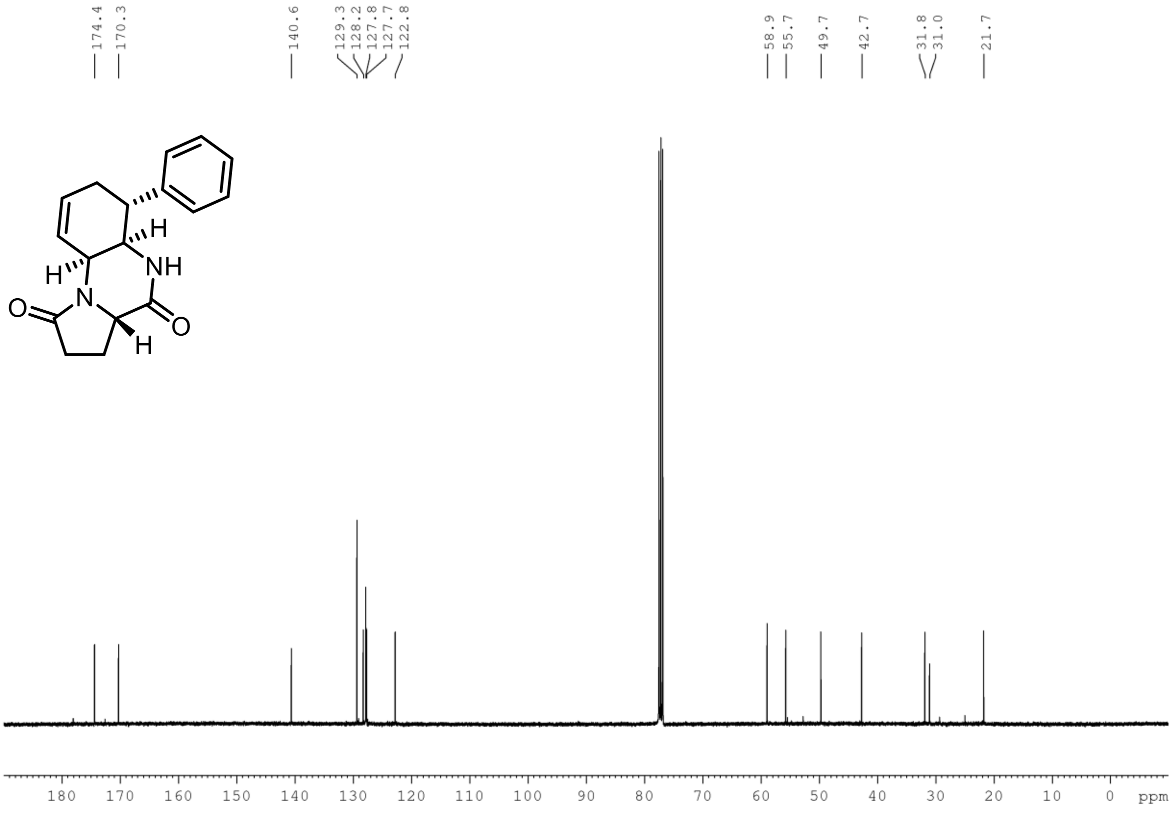

Methyl (*S*,*E*)-5-oxo-1-(penta-2,4-dien-1-yl)pyrrolidine-2-carboxylate (S4)

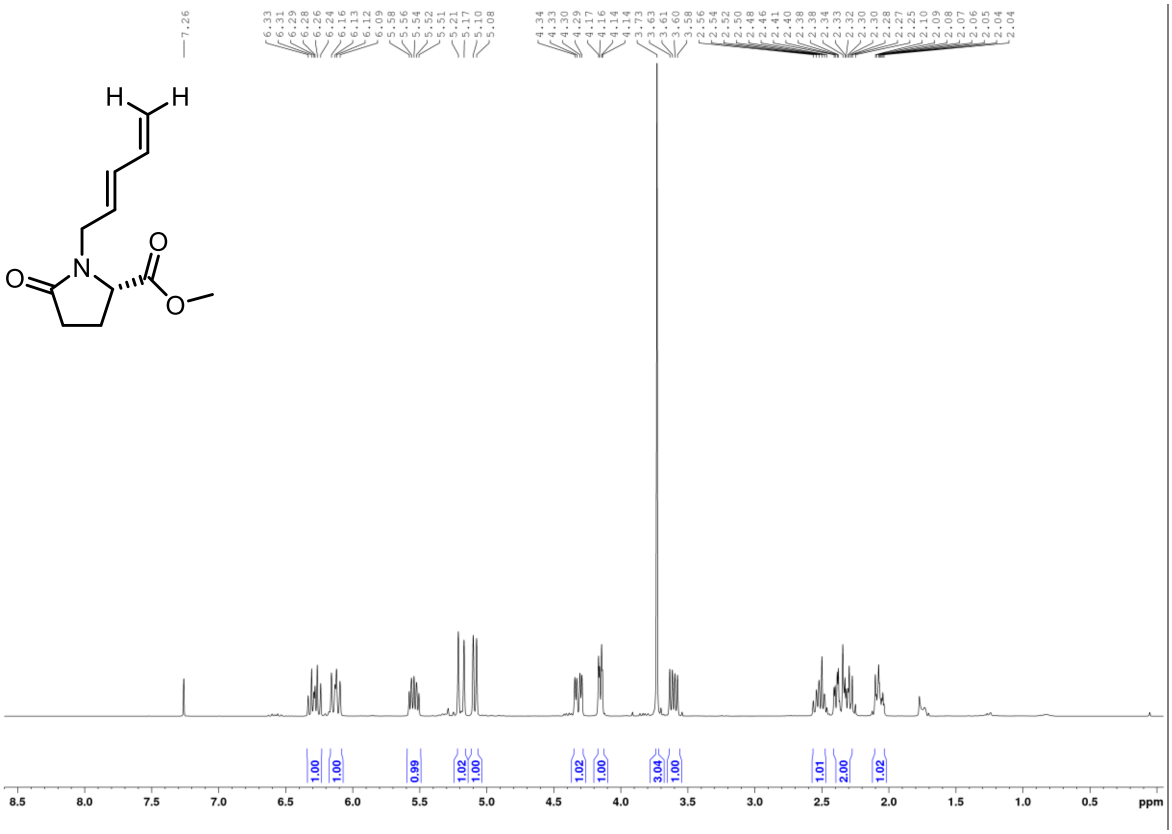

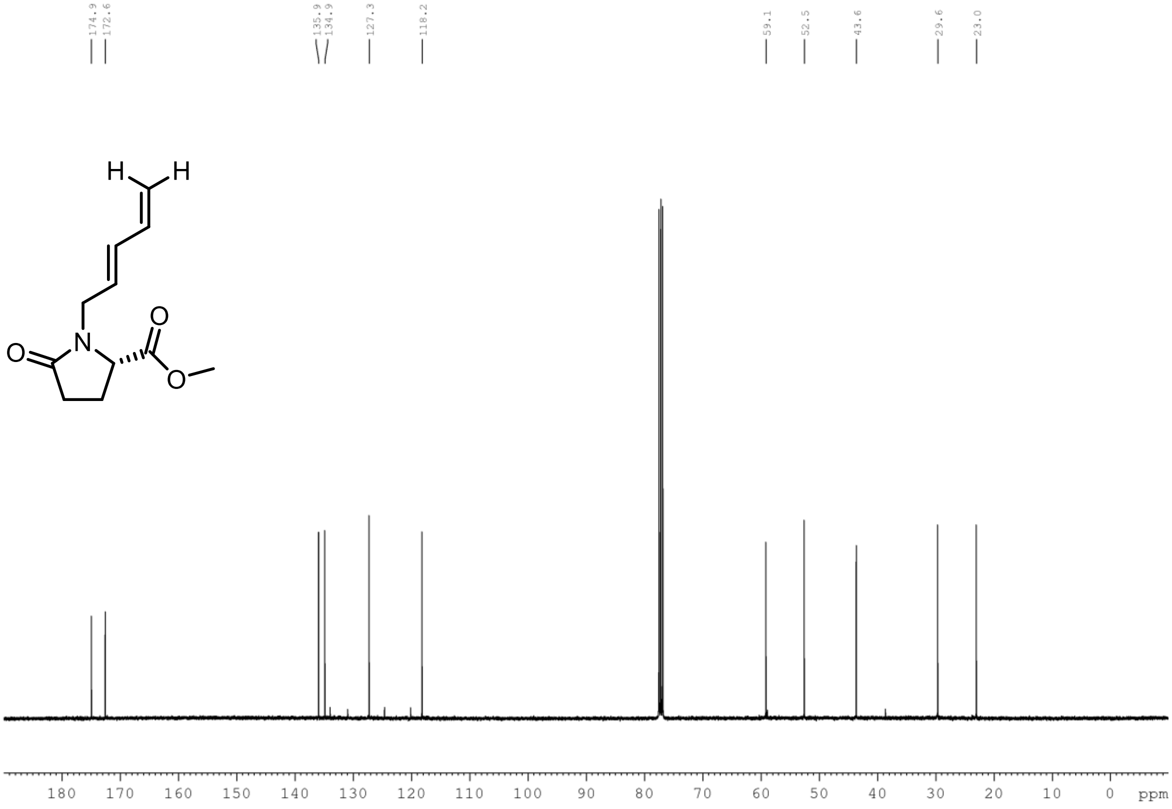

(*S*,*E*)-*N*-Hydroxy-5-oxo-1-(penta-2,4-dien-1-yl)pyrrolidine-2-carboxamide (S5)

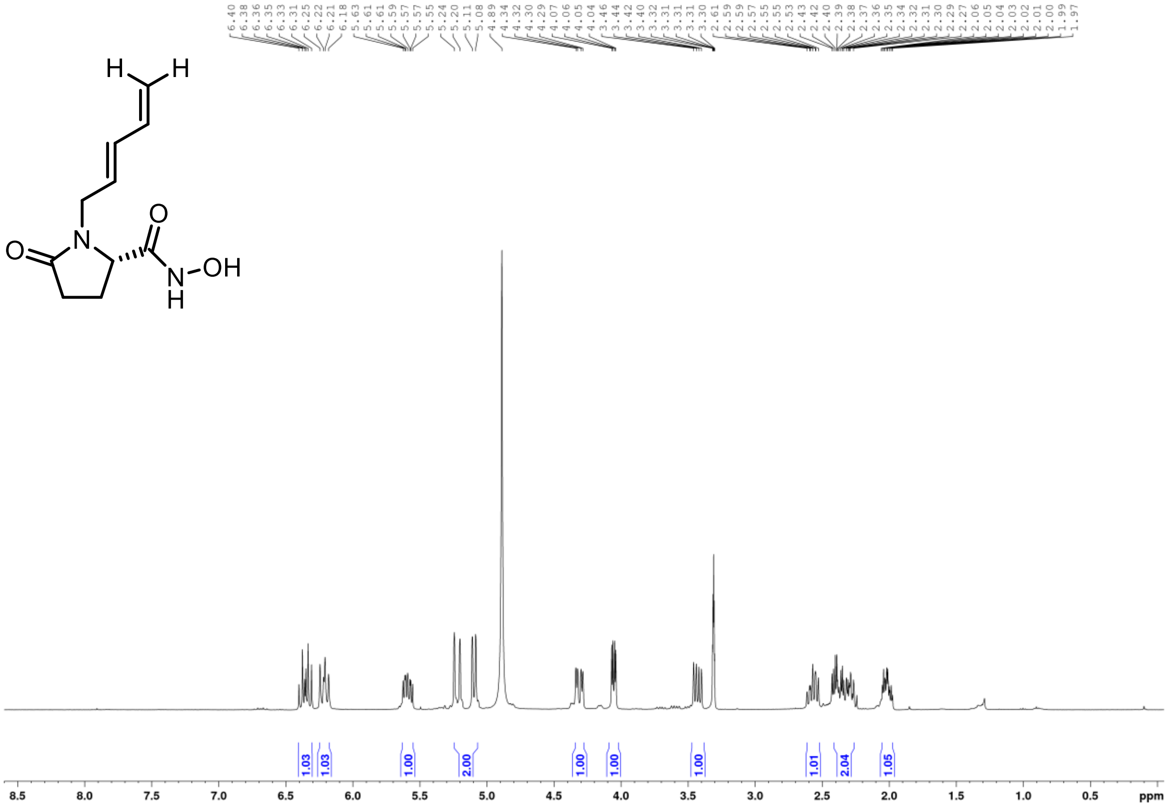

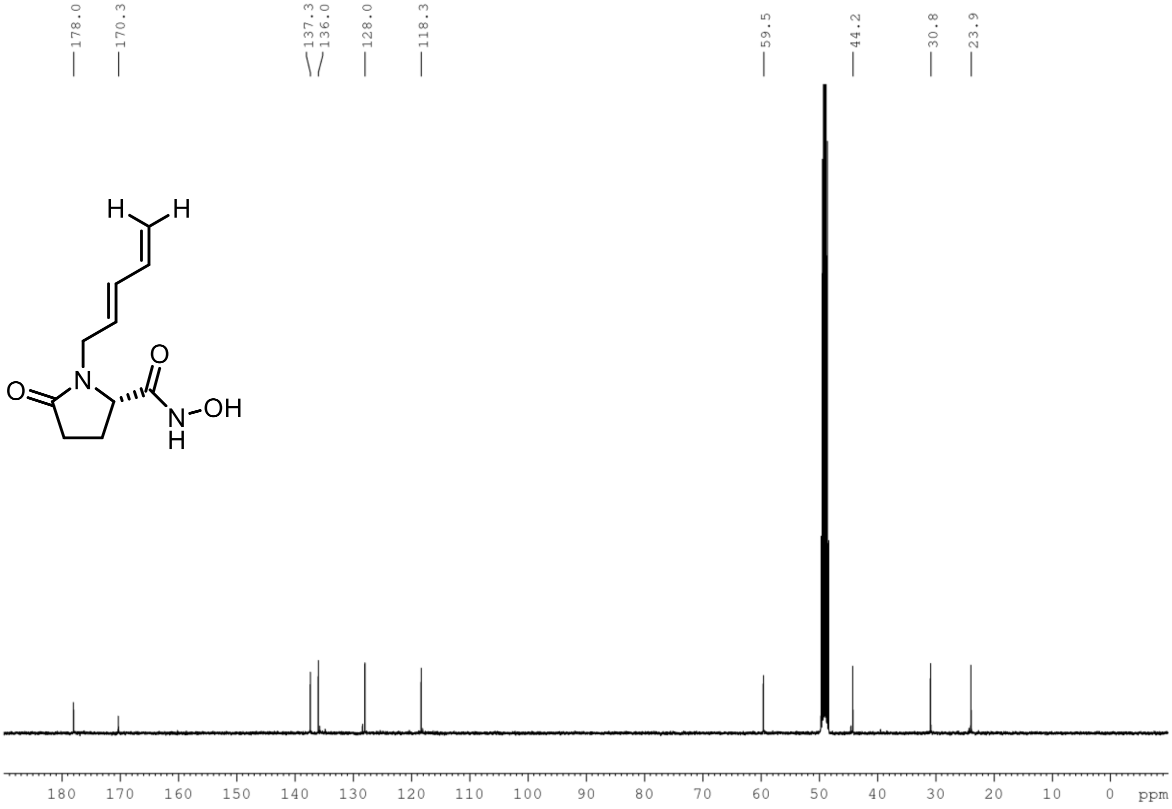

(9a*S*)-4a,5,9,9a-Tetrahydro-2*H*,7*H*-pyrrolo[1',2':4,5]pyrazino[1,2-*b*][1,2]oxazine-7,10(8*H*)-dione (8)

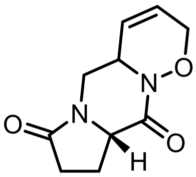

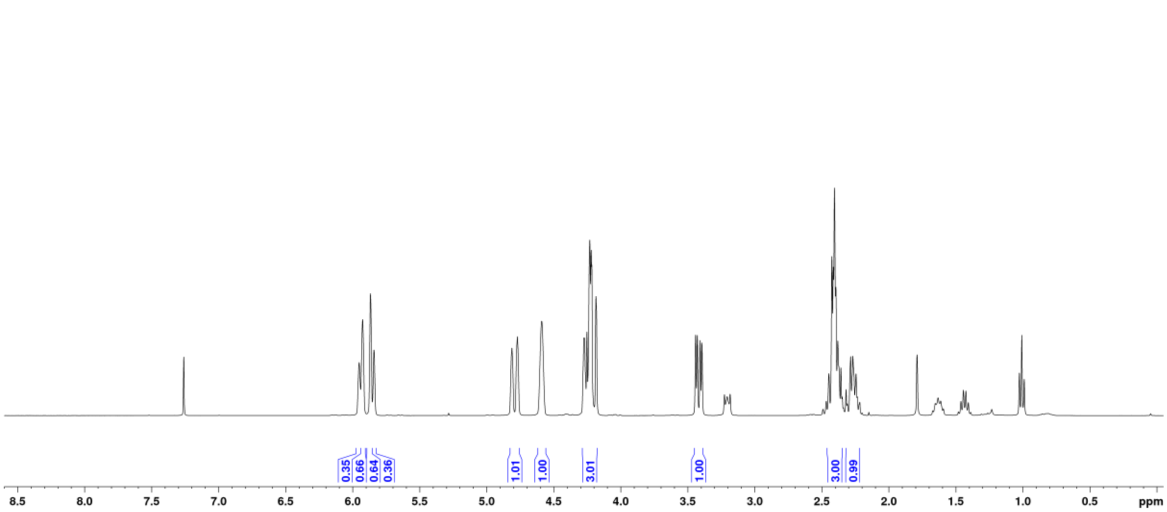

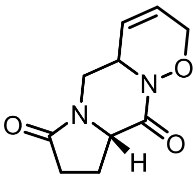

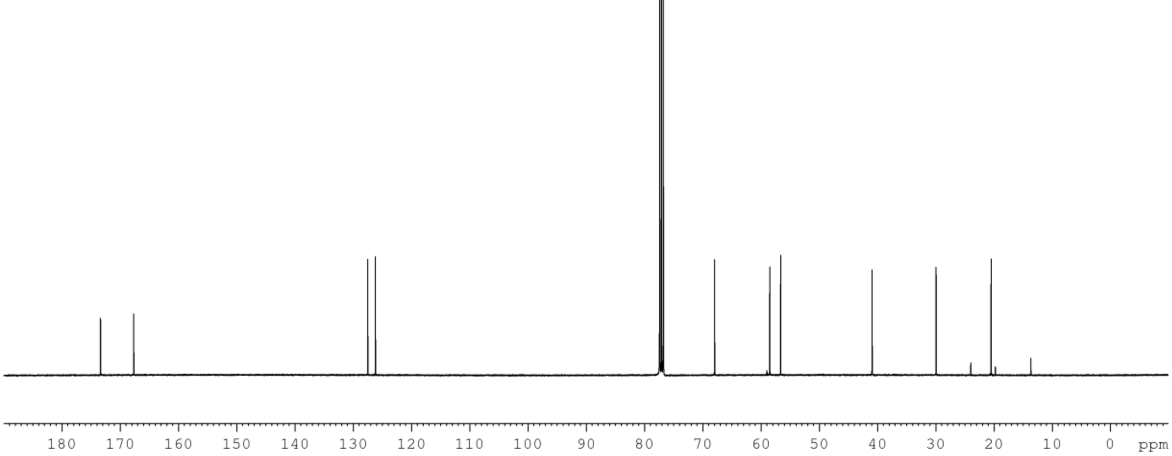

Methyl (*S*)-5-oxo-1-(prop-2-yn-1-yl)pyrrolidine-2-carboxylate (S6)

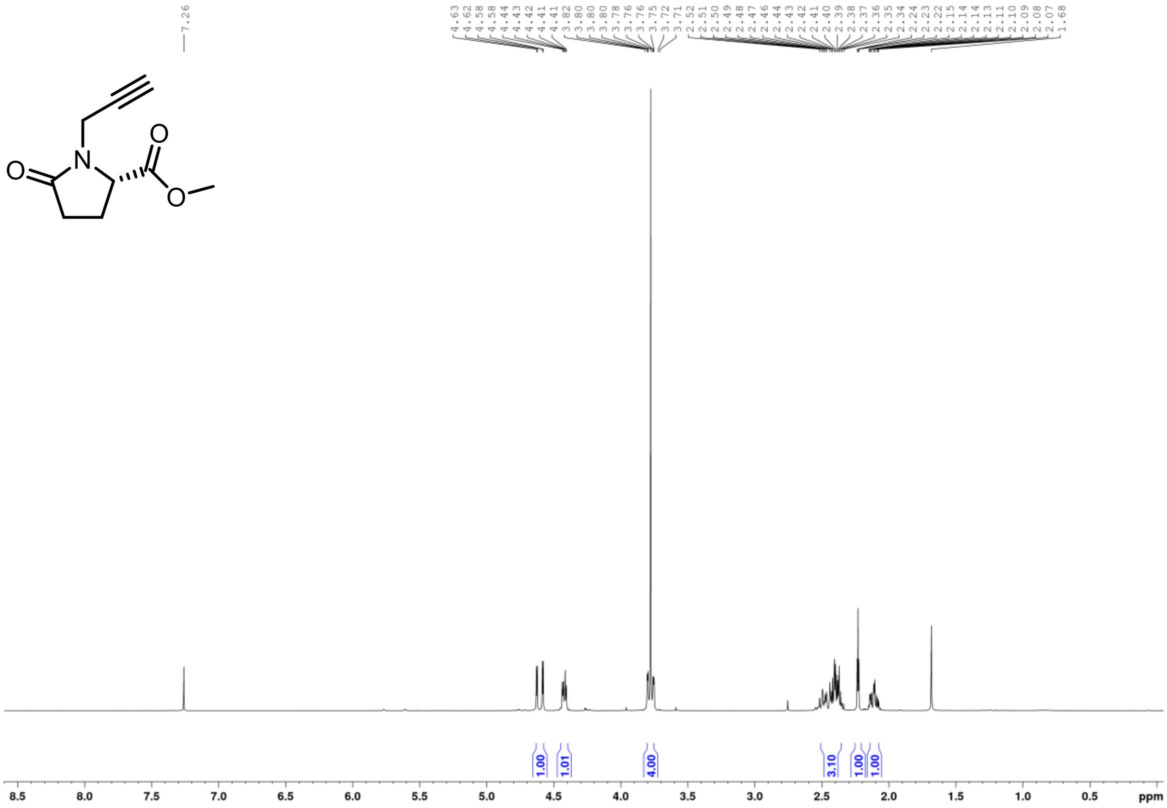

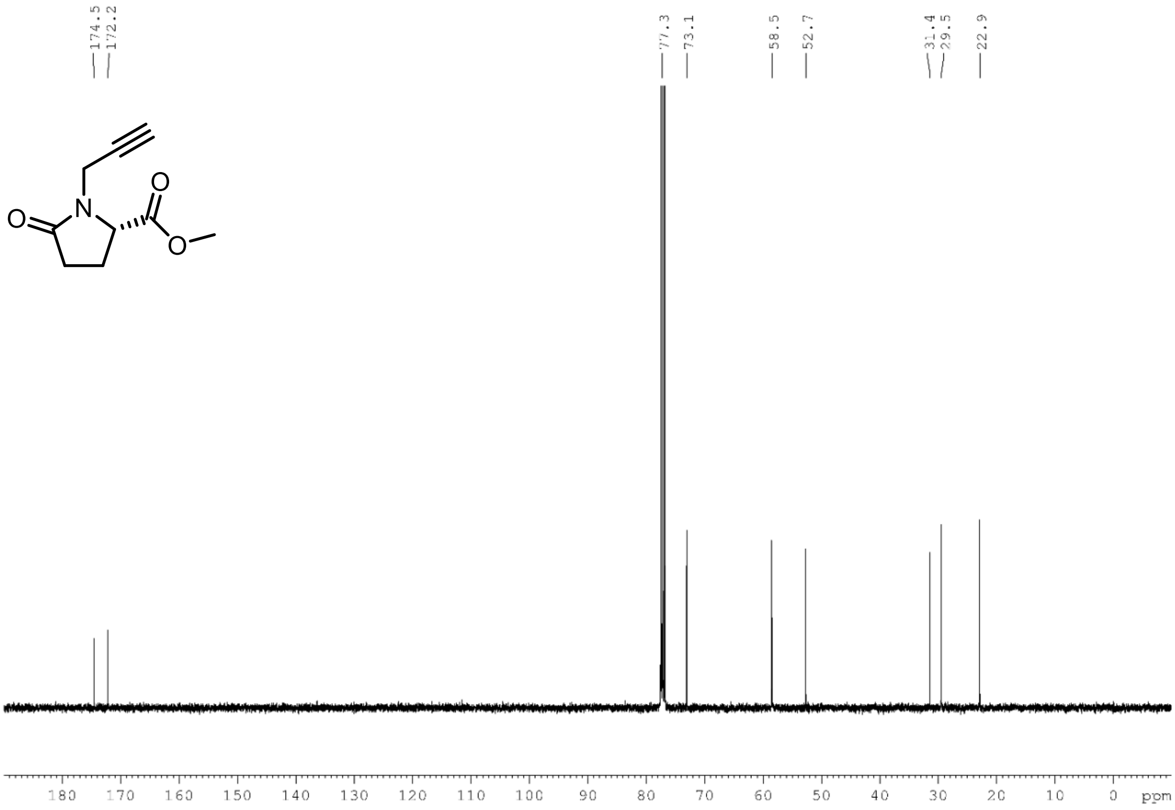

(*S*)-*N*-Methyl-5-oxo-1-(prop-2-yn-1-yl)pyrrolidine-2-carboxamide (S7)

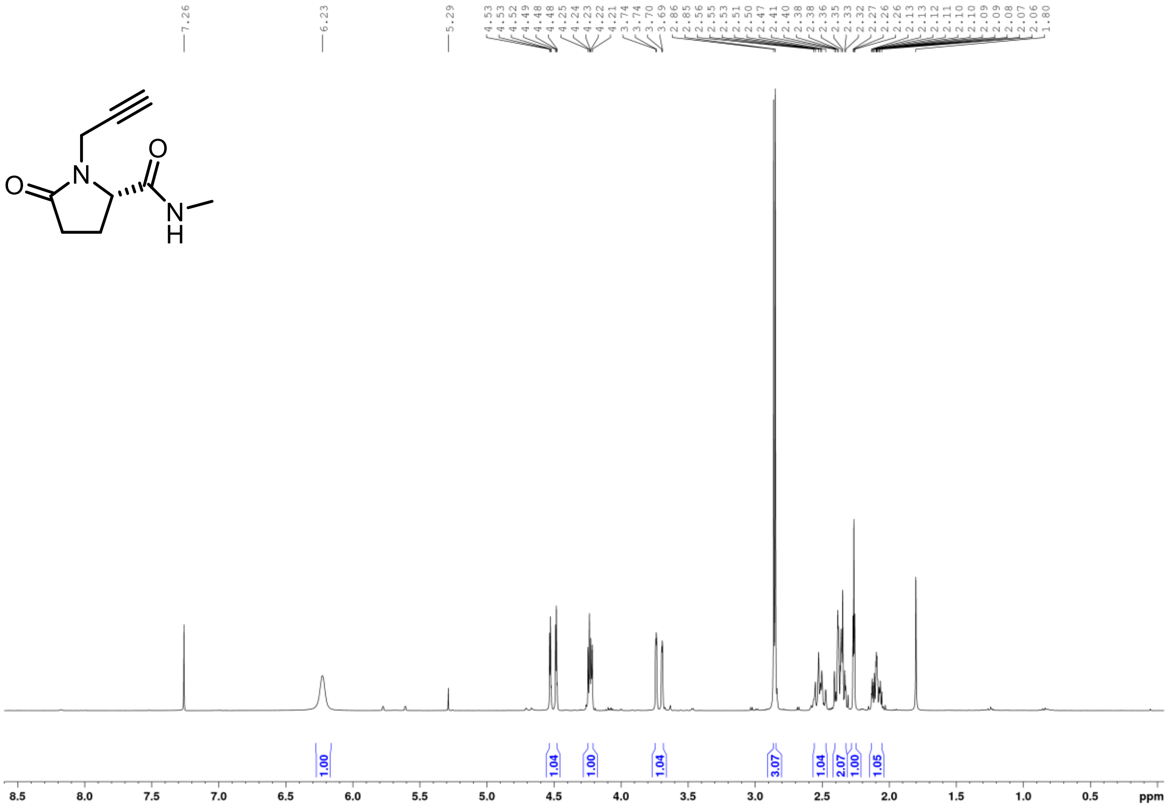

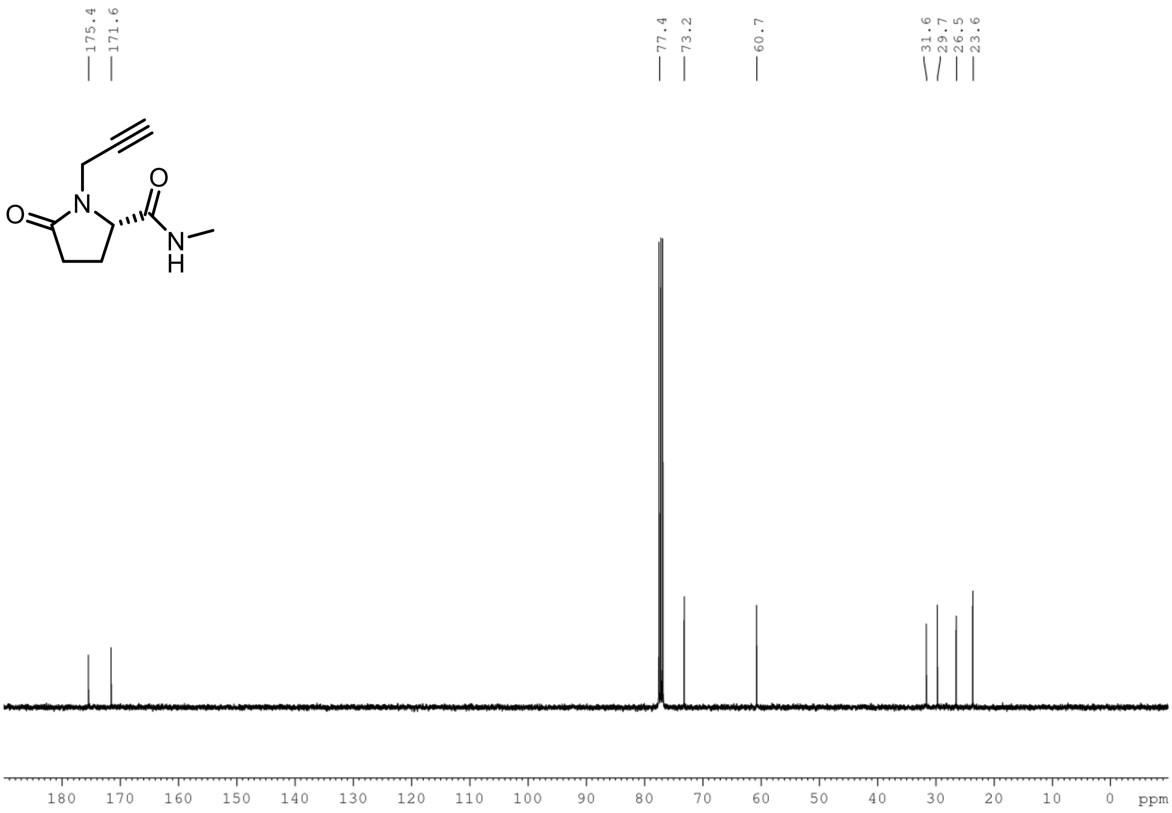

(*S*)-2,3-Dimethyl-8,8a-dihydropyrrolo[1,2-*a*]pyrazine-1,6(2*H*,7*H*)-dione (9)

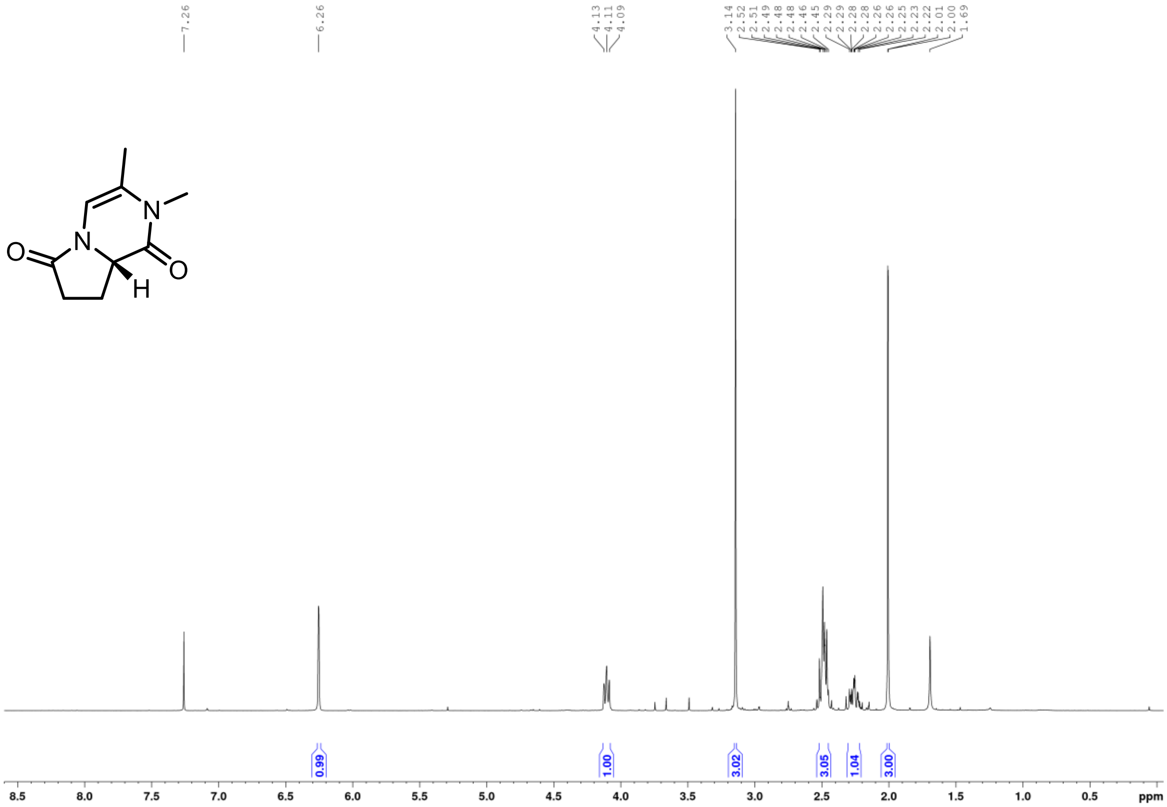

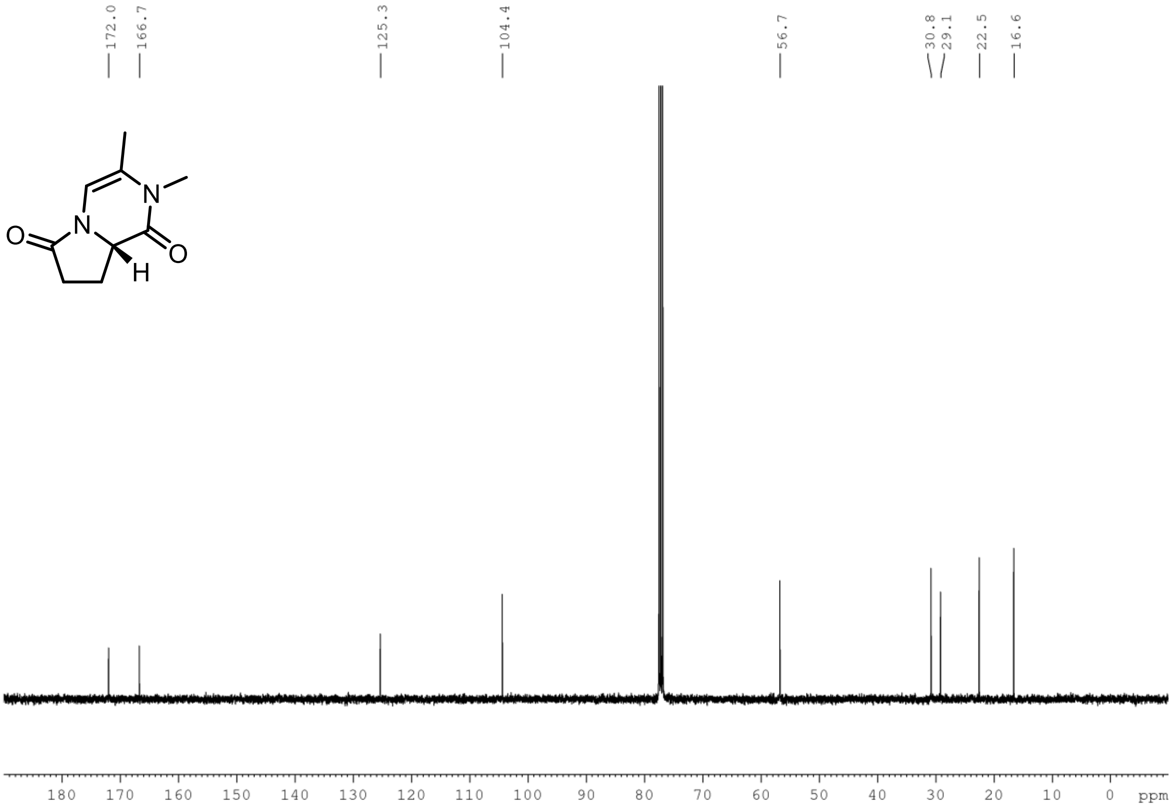

Methyl (*S*)-1-((*S*)-2-nitro-1-phenylethyl)-5-oxopyrrolidine-2-carboxylate (S8-*anti*)

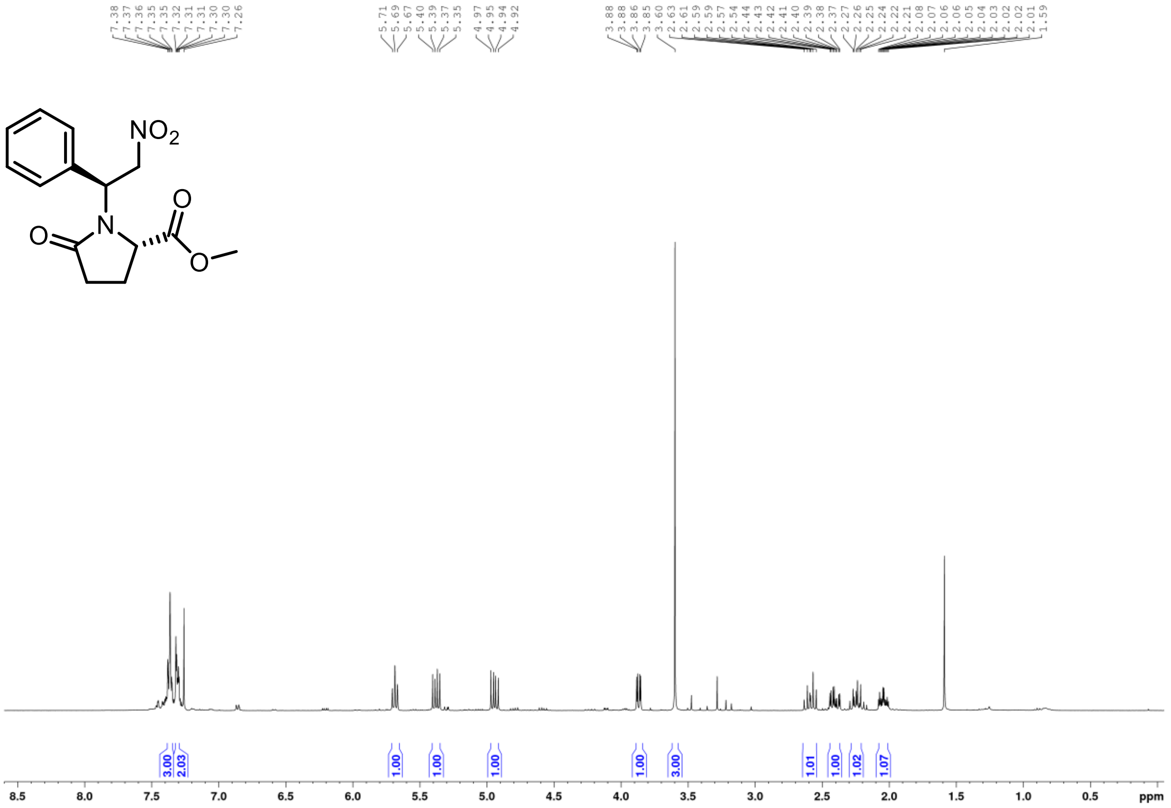

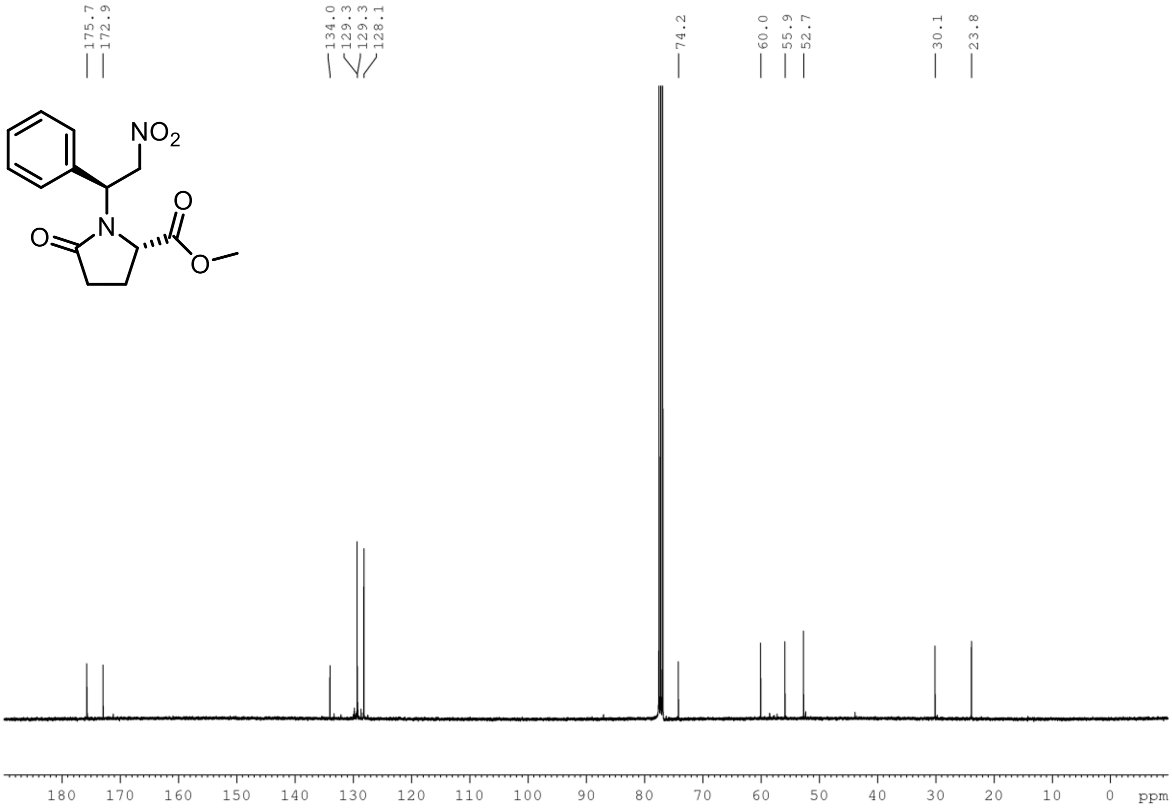

Methyl (*S*)-1-((*R*)-2-nitro-1-phenylethyl)-5-oxopyrrolidine-2-carboxylate (S8-*syn*)

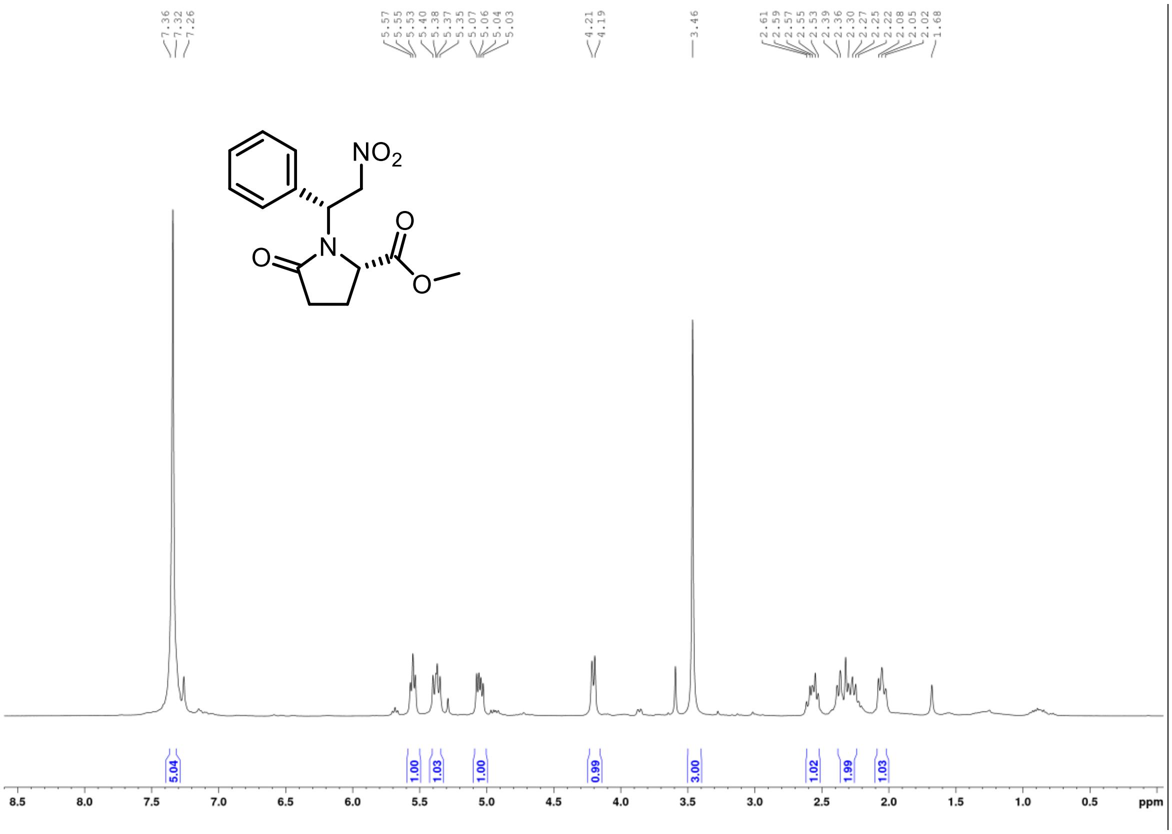

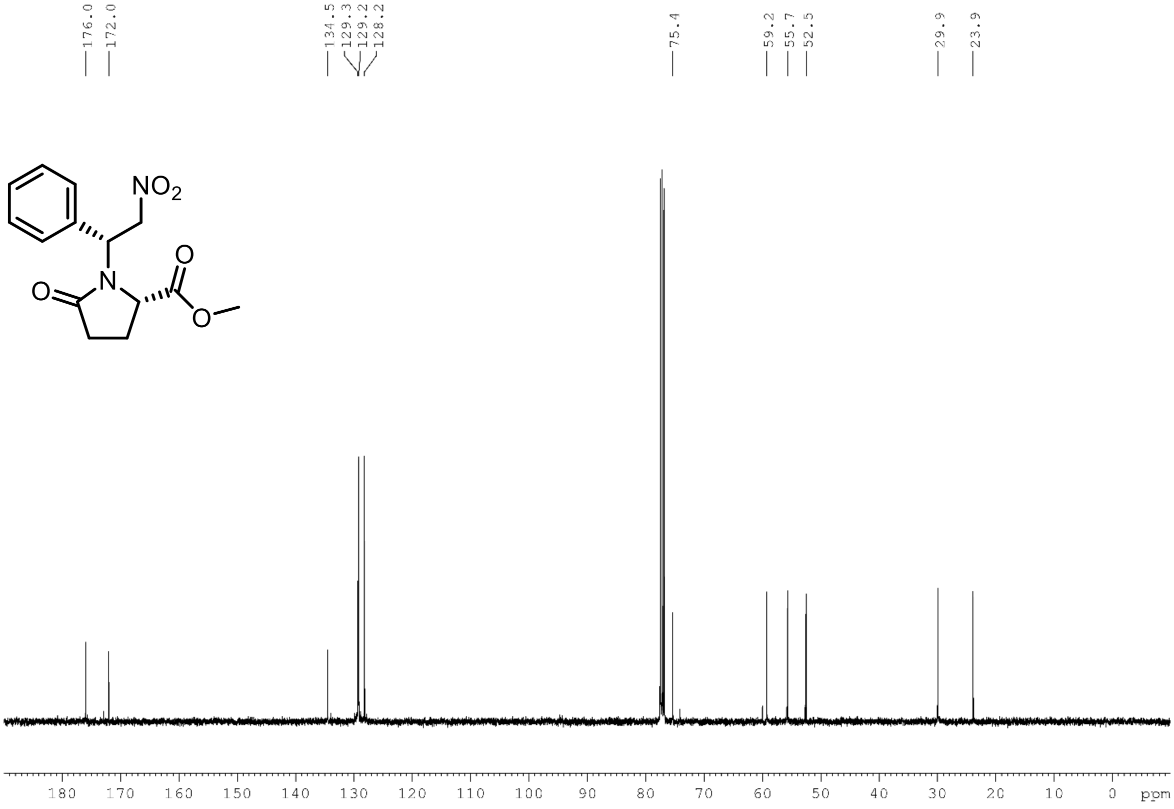

(4*S*,8a*S*)-4-Phenyltetrahydropyrrolo[1,2-*a*]pyrazine-1,6(2*H*,7*H*)-dione (10)

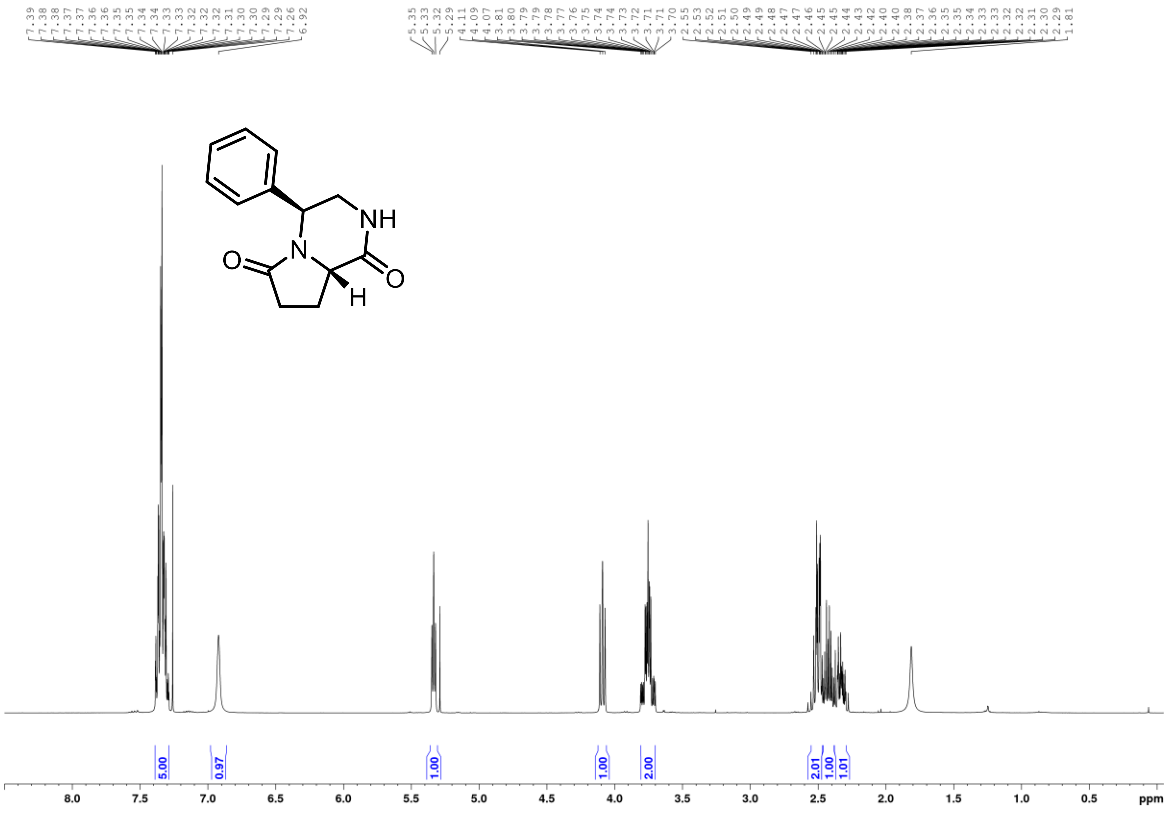

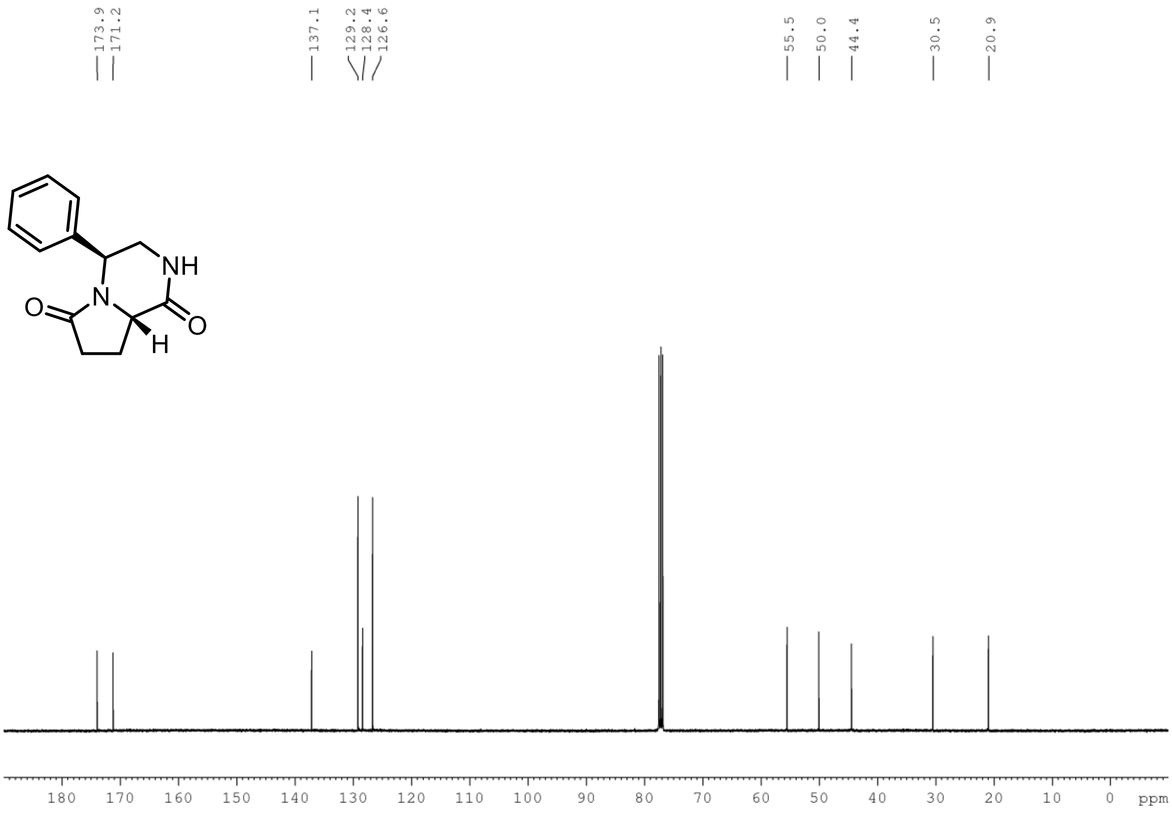

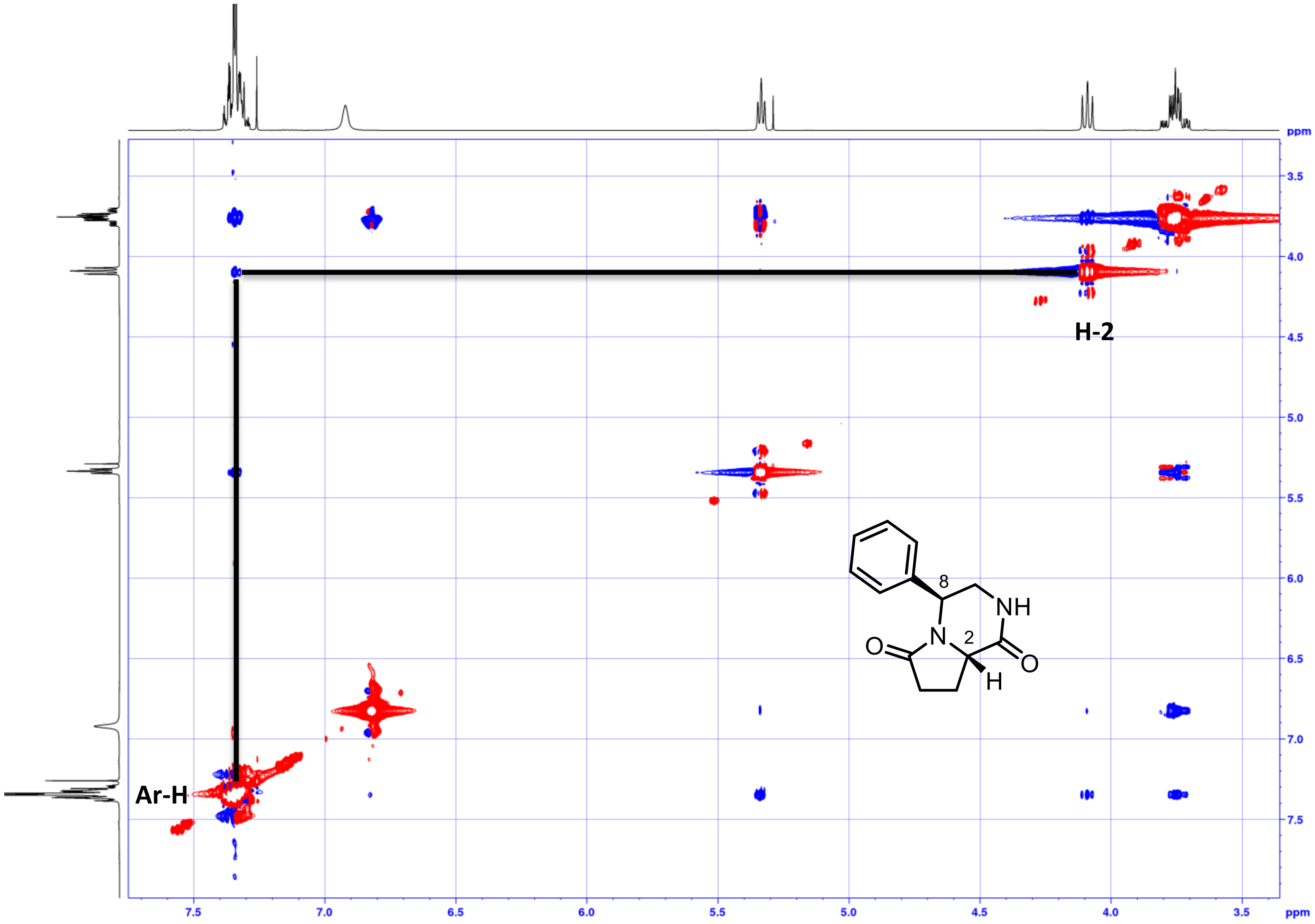
H-2
Ar-H
ppm
NH
N
O
H
8
2

Methyl (*S*)-1-(2-cyano-4-nitrophenyl)-5-oxopyrrolidine-2-carboxylate (11)

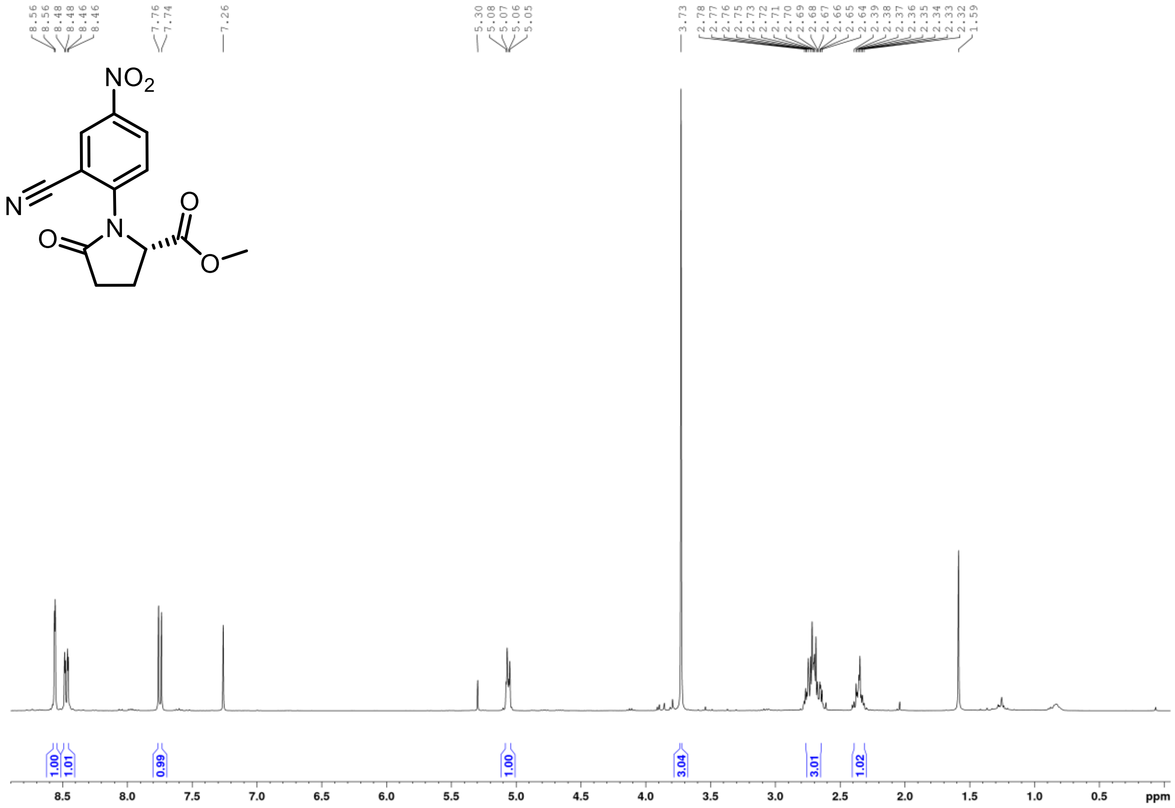

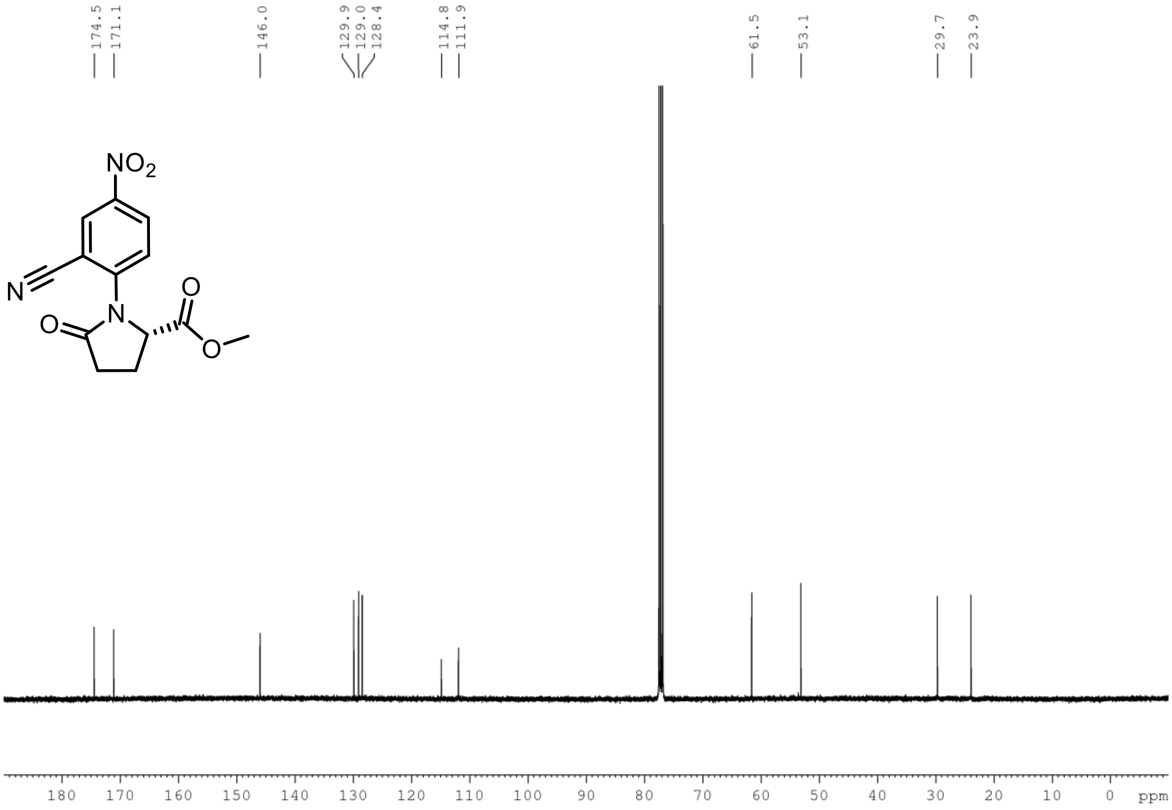

Methyl (3a*R*,6*S*,8a*S*,8b*S*)-2-methyl-1,3-dioxo-8a-phenyloctahydro-1*H*-dipyrrolo[1,2-*b*:3',4'-*d*]isoxazole-6-carboxylate (12)

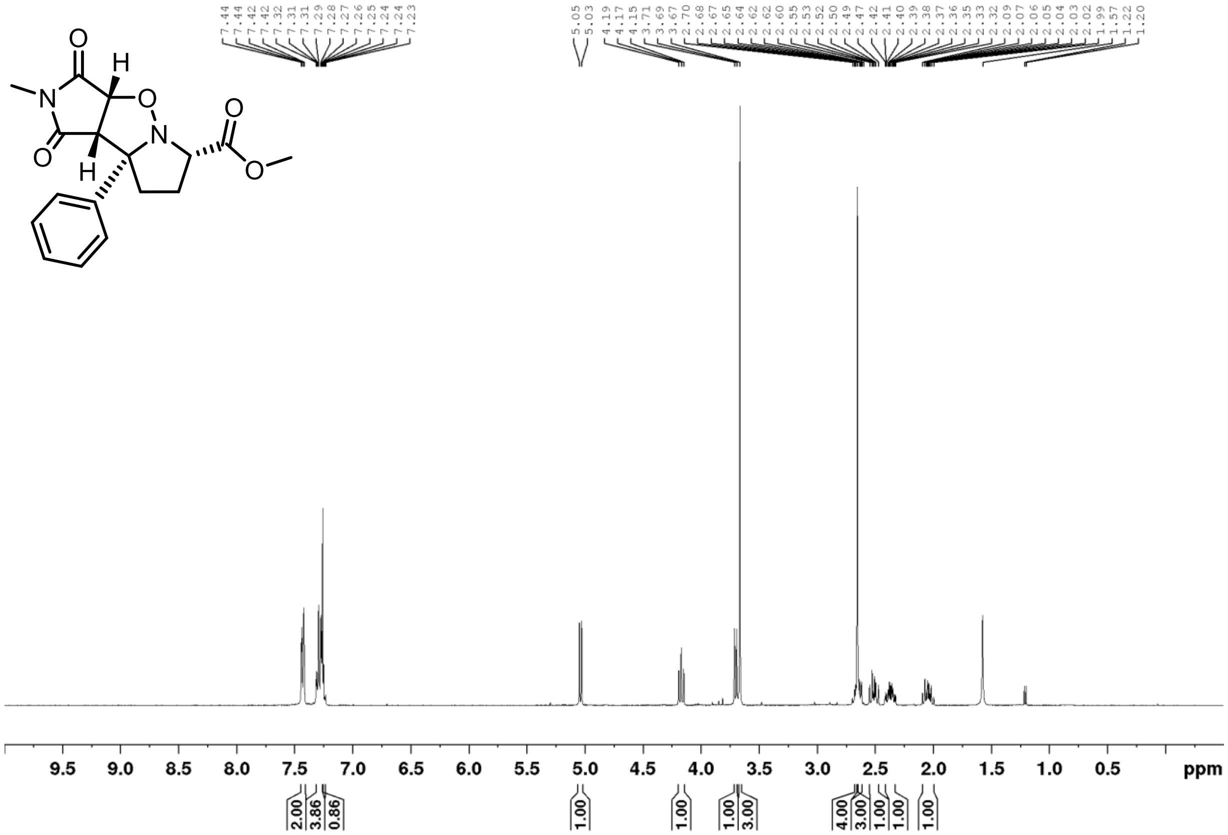

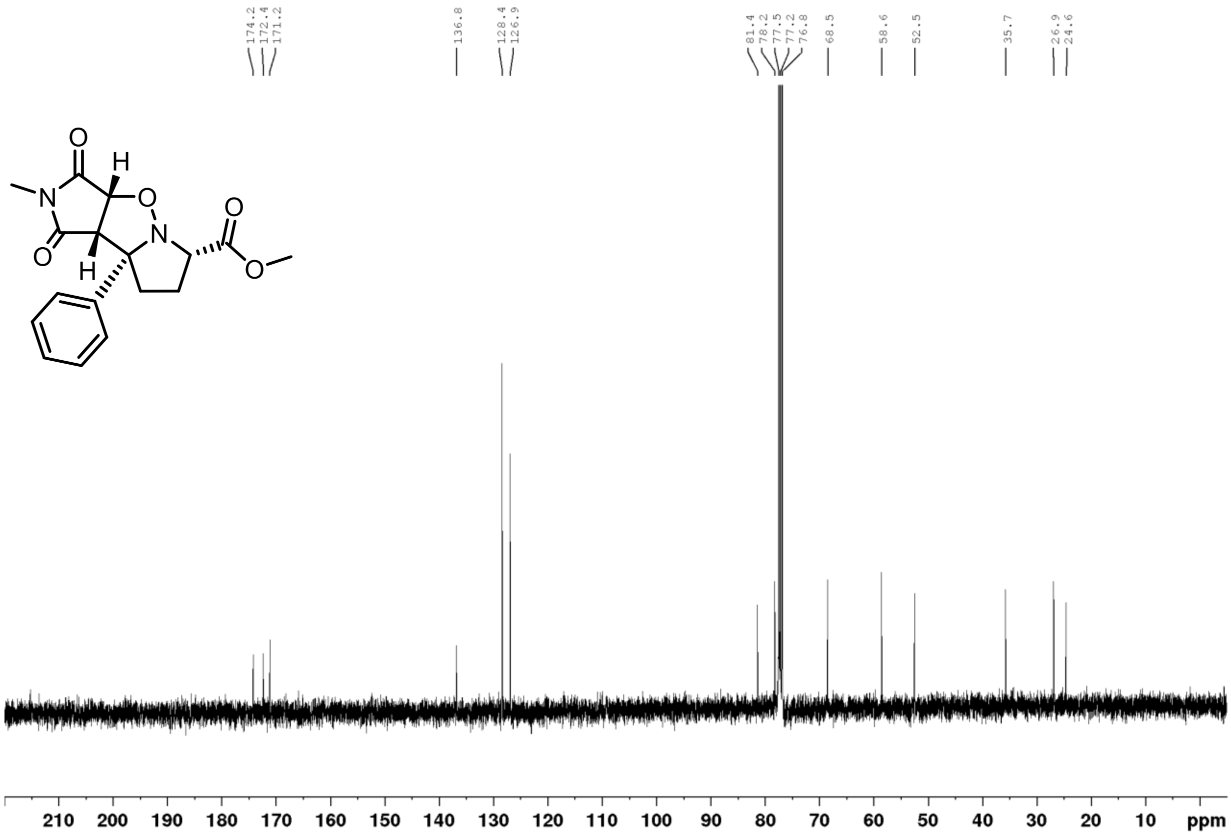

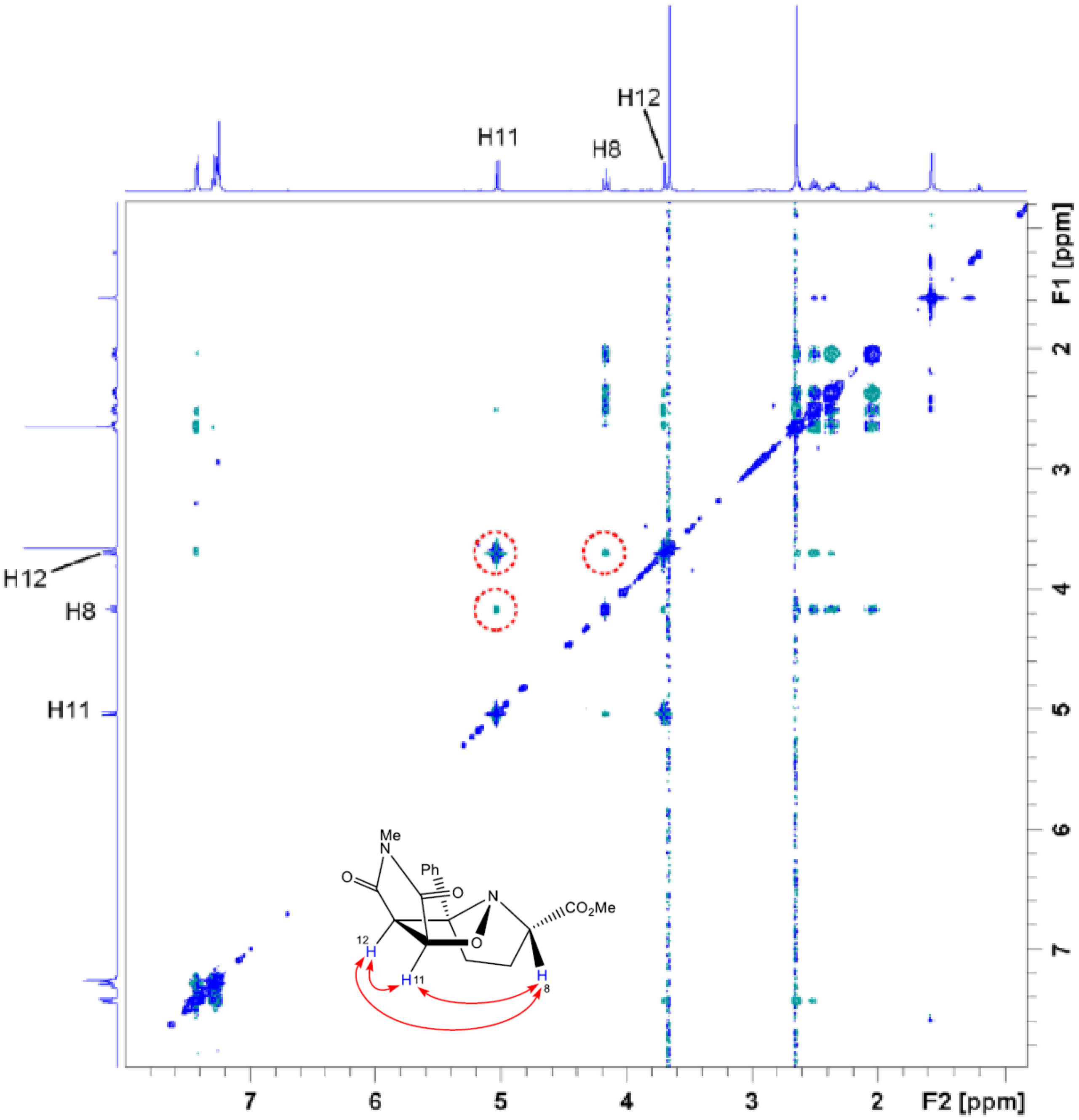
H12
H11
H8
H12
H8
H11
F1 [ppm]
F2 [ppm]
Me
N
O
Ph
O
N
CO_2Me
O
12
H
H11
H
8

Dimethyl (3a*R*,6*S*)-3a-phenyl-3a,4,5,6-tetrahydropyrrolo[1,2-*b*]isoxazole-3,6-dicarboxylate (13)

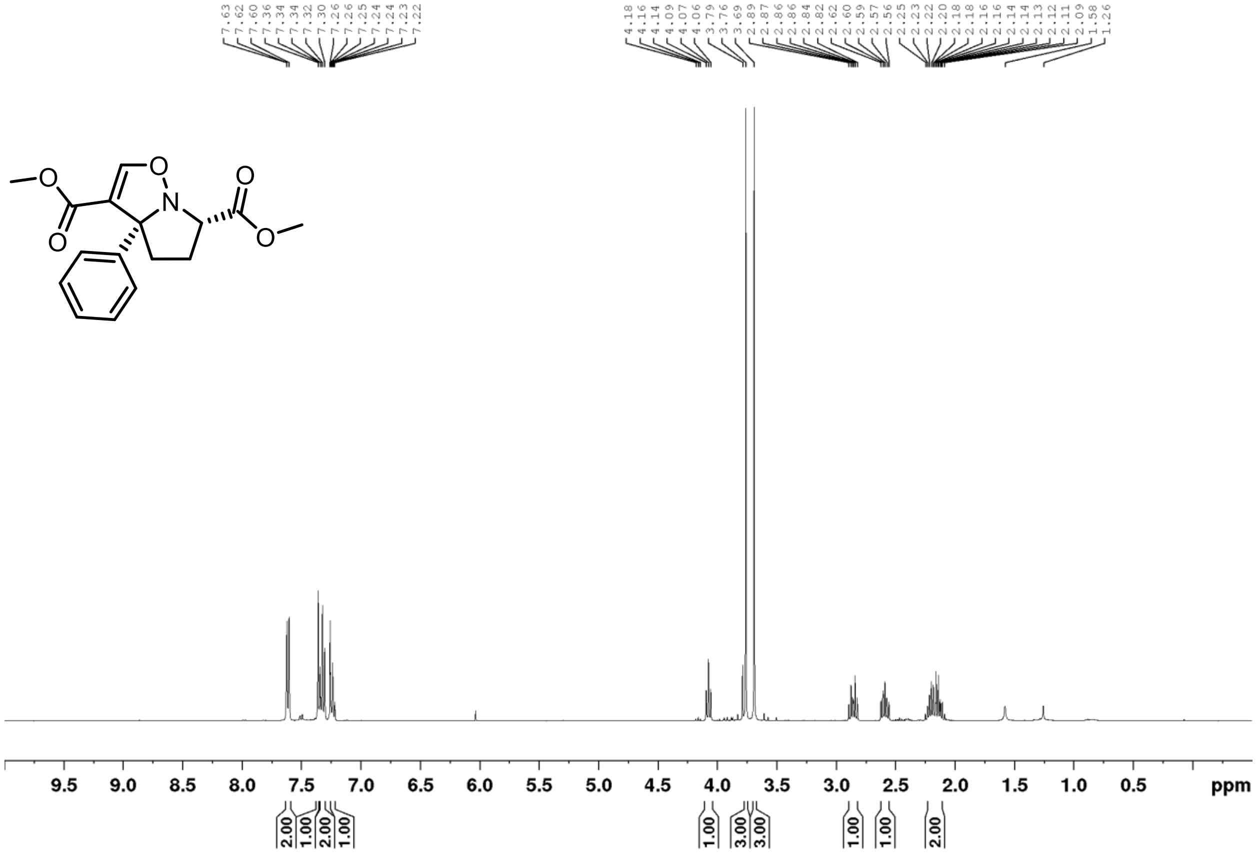

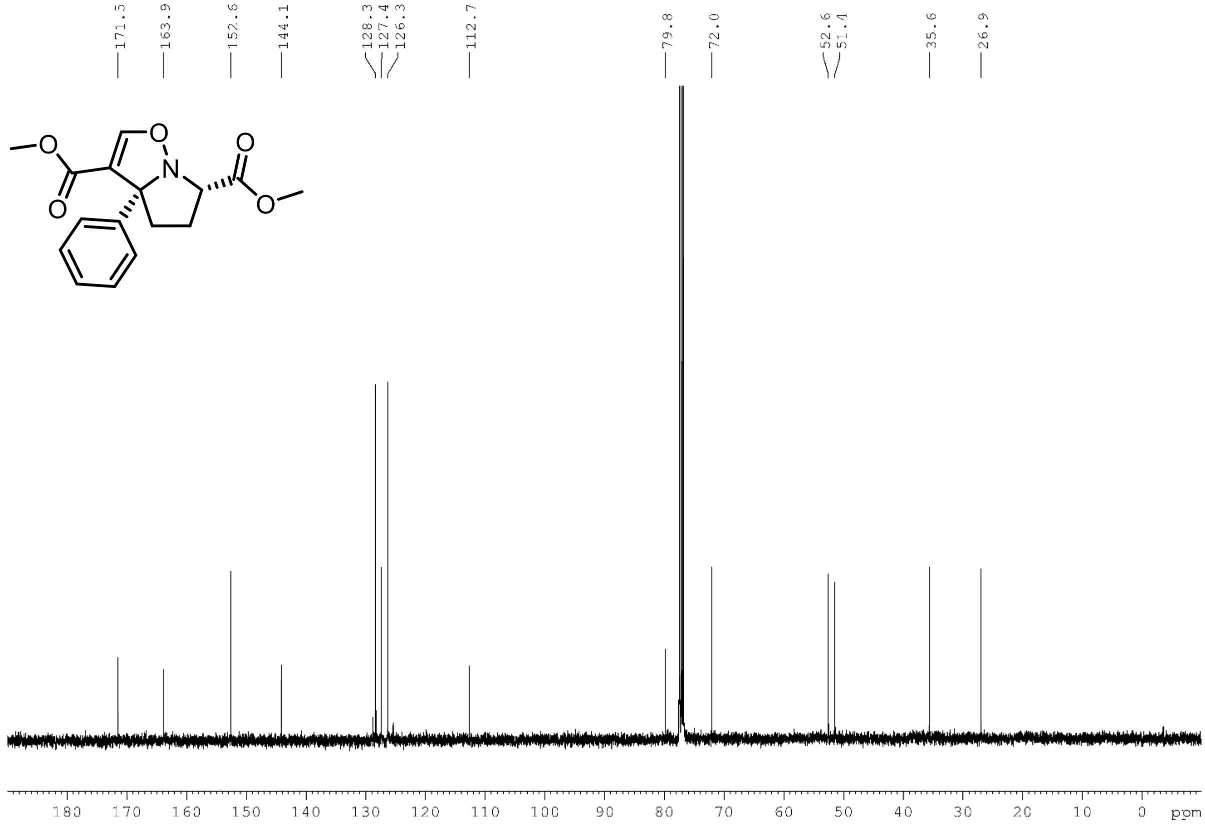

1-(*tert*-butyl) 2-Methyl (2*S*,5*S*)-5-((trimethylsilyl)ethynyl)pyrrolidine-1,2-dicarboxylate (S10)

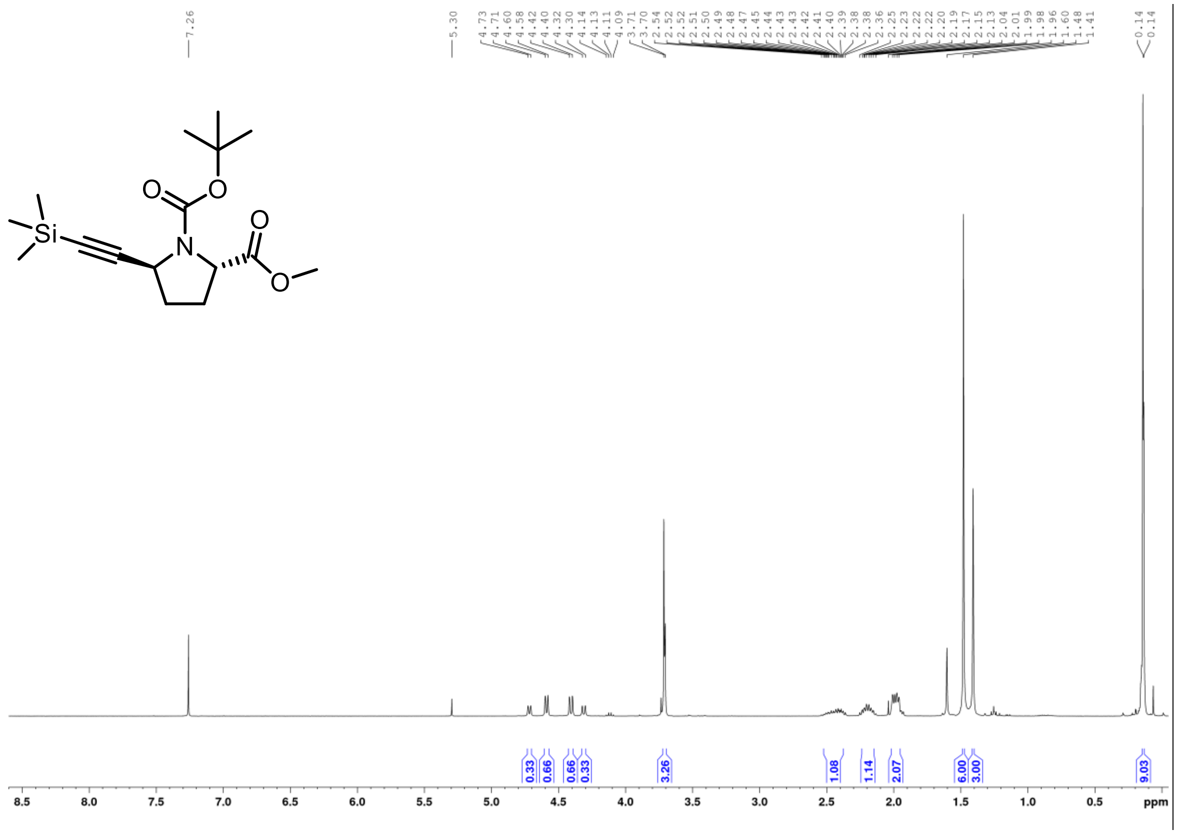

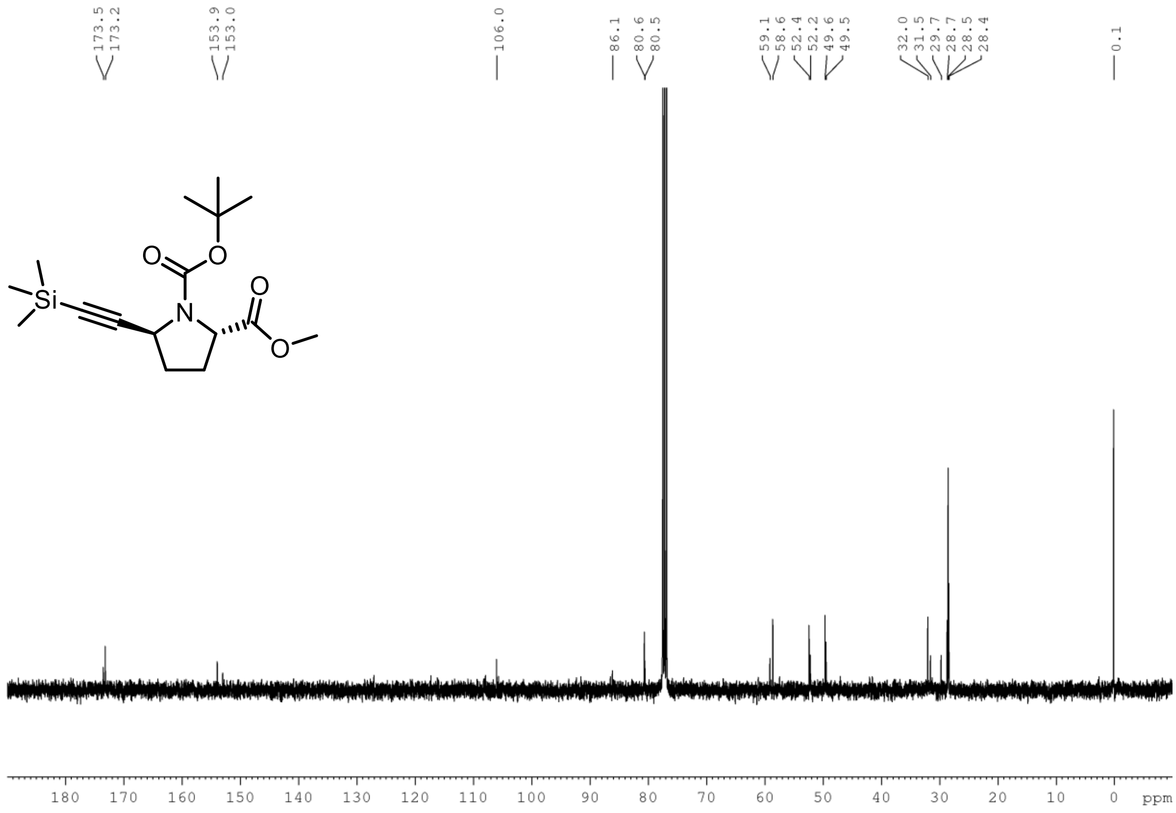

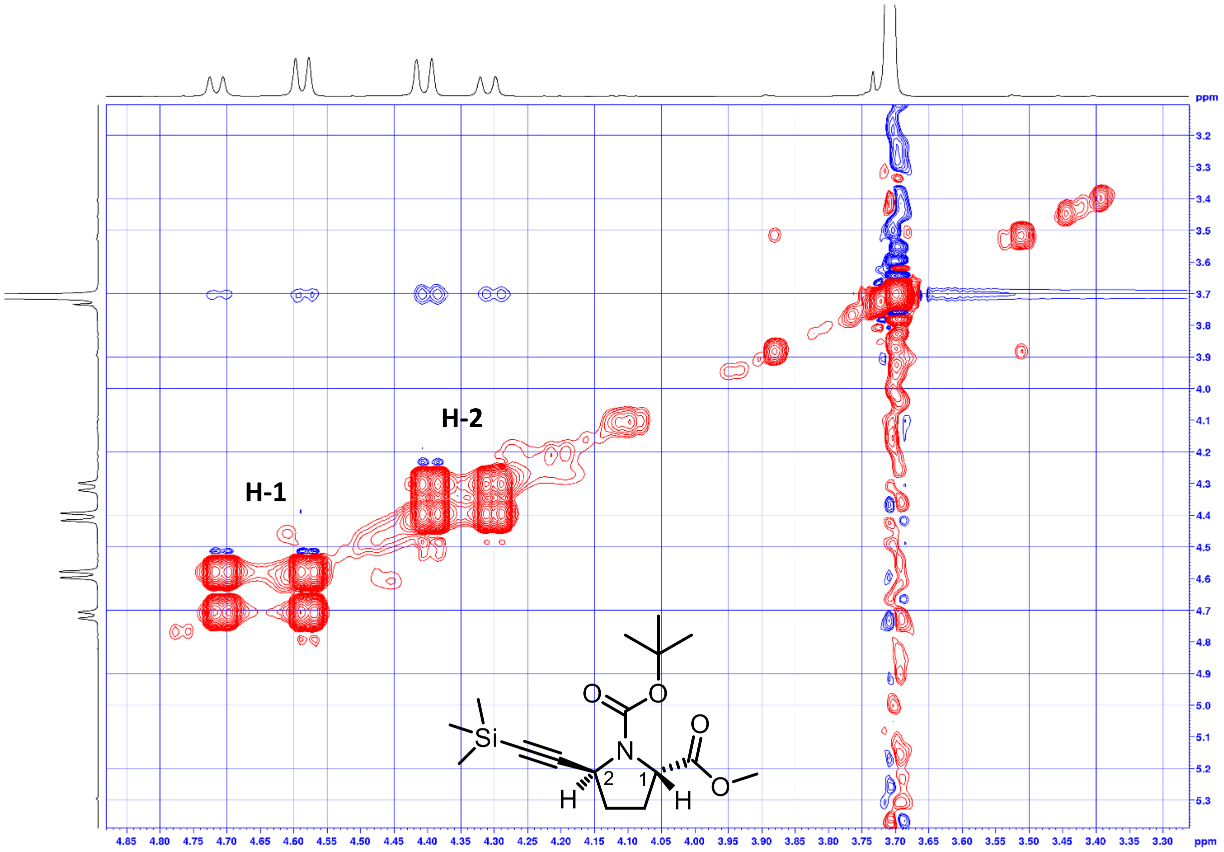

H-1
H-2
ppm
O
O
O
O
N
Si
H
H
2
1

1-(*tert*-butyl) 2-Methyl (2*S*,5*S*)-5-ethynylpyrrolidine-1,2-dicarboxylate (S11)

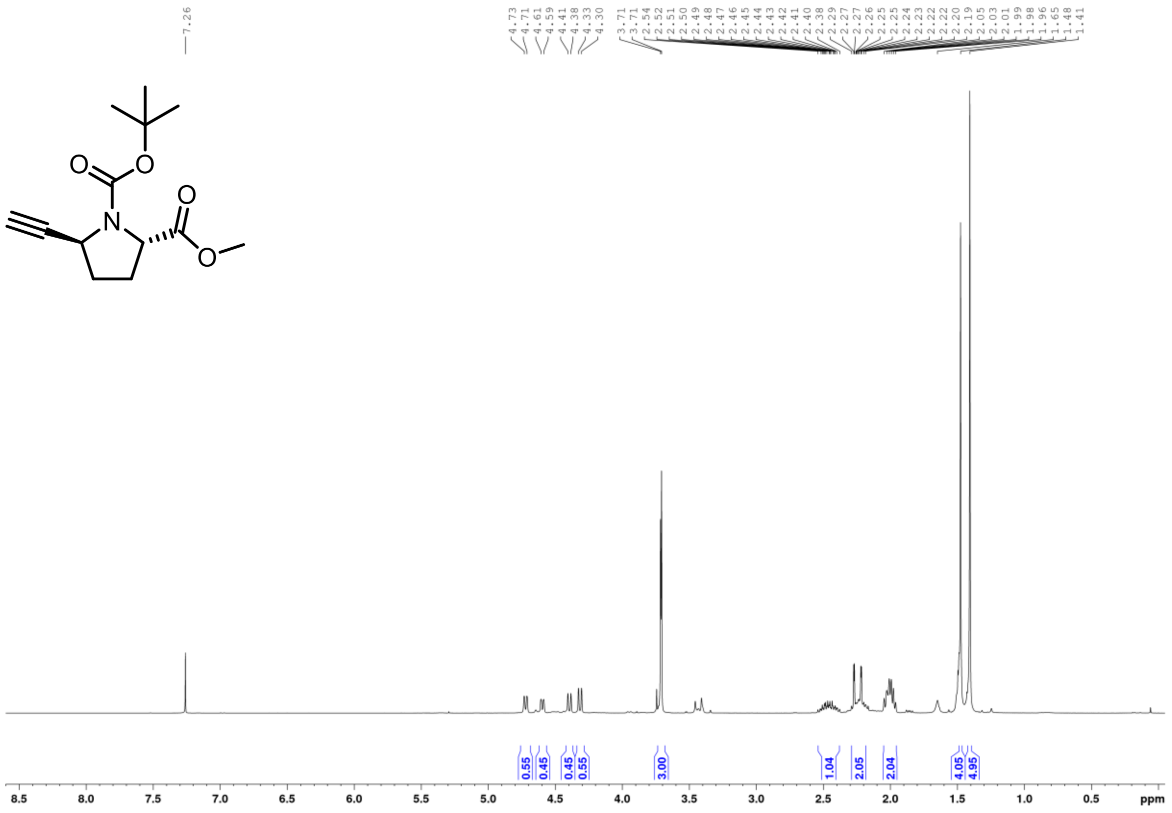

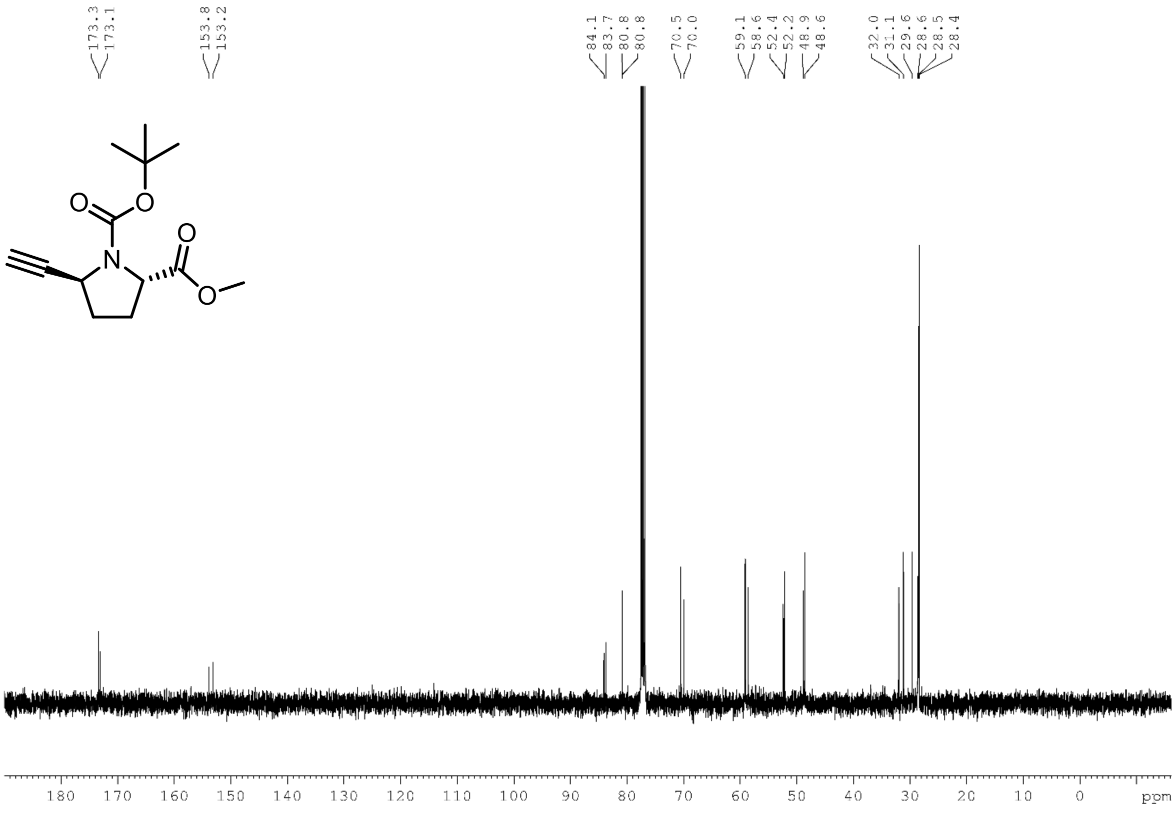

Methyl (2*S*,5*S*)-5-ethynylpyrrolidine-2-carboxylate (S12)

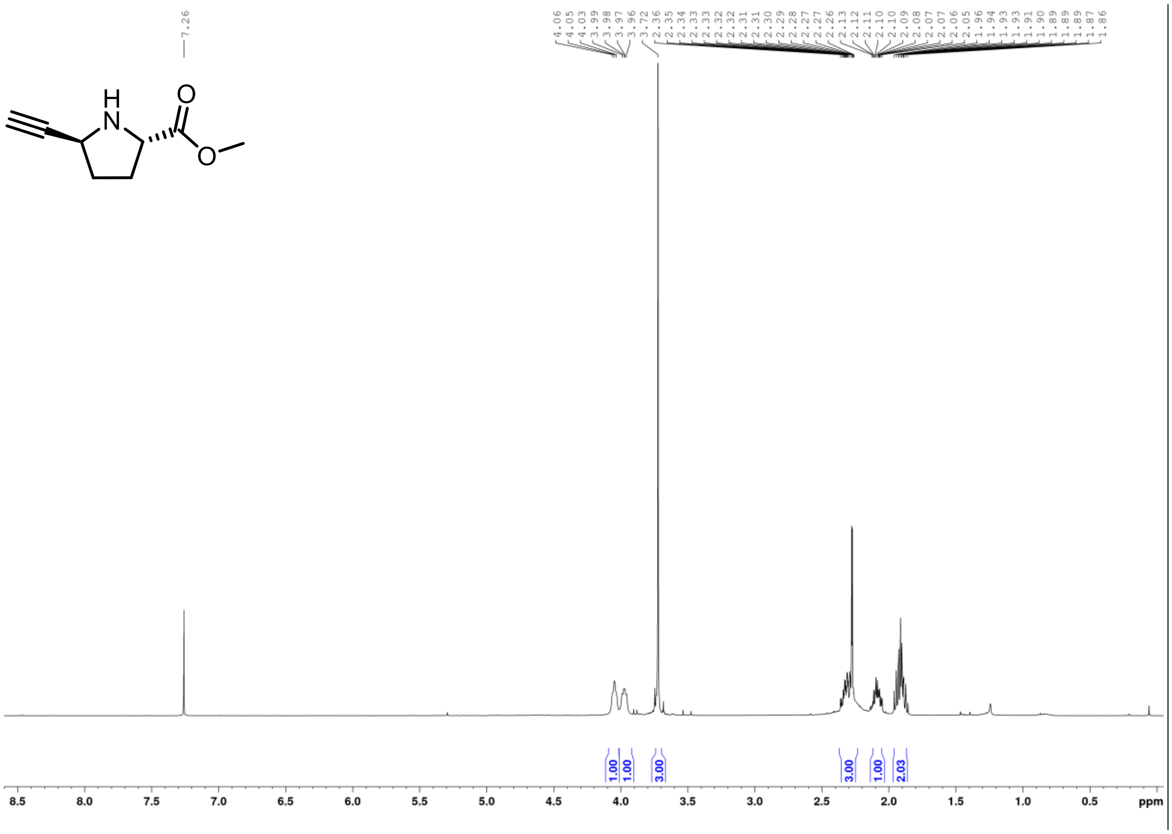

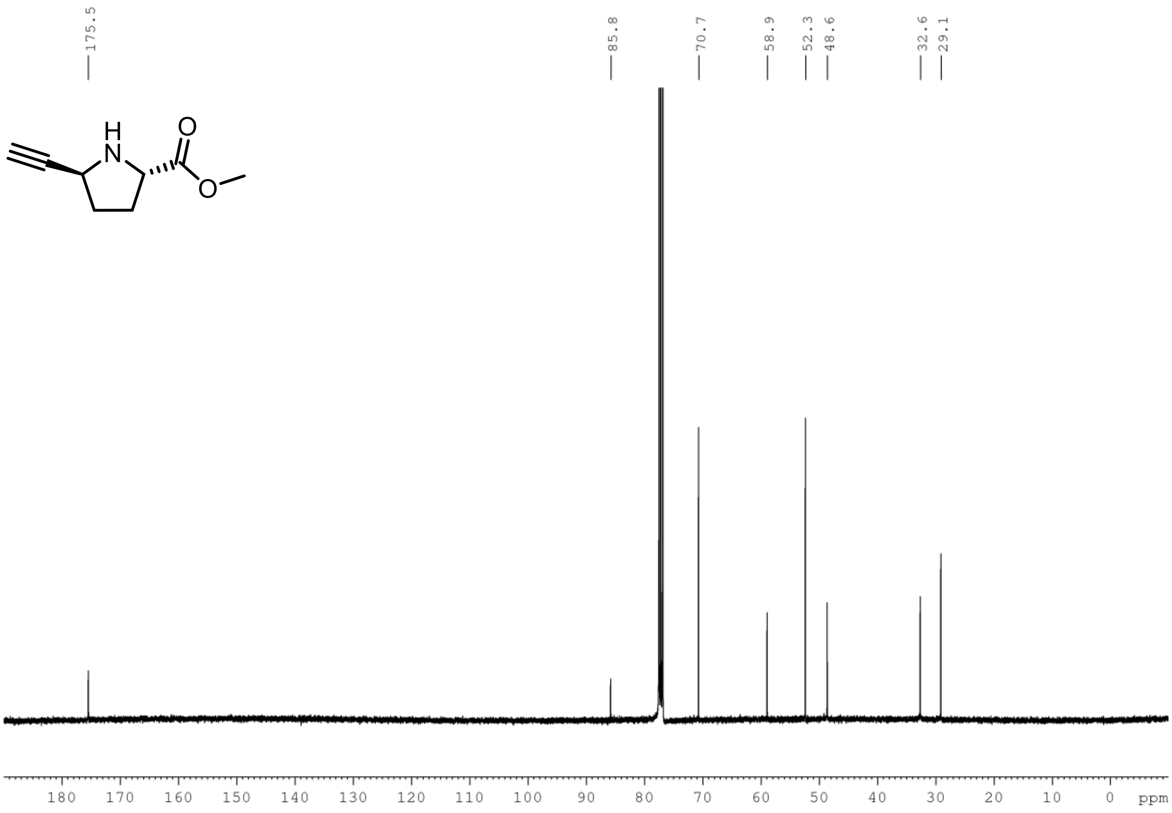

Methyl (2*S*,5*S*)-1-(*N*-(*tert*-butoxycarbonyl)-*N*-methylglycyl)-5-ethynylpyrrolidine-2-carboxylate (S13)

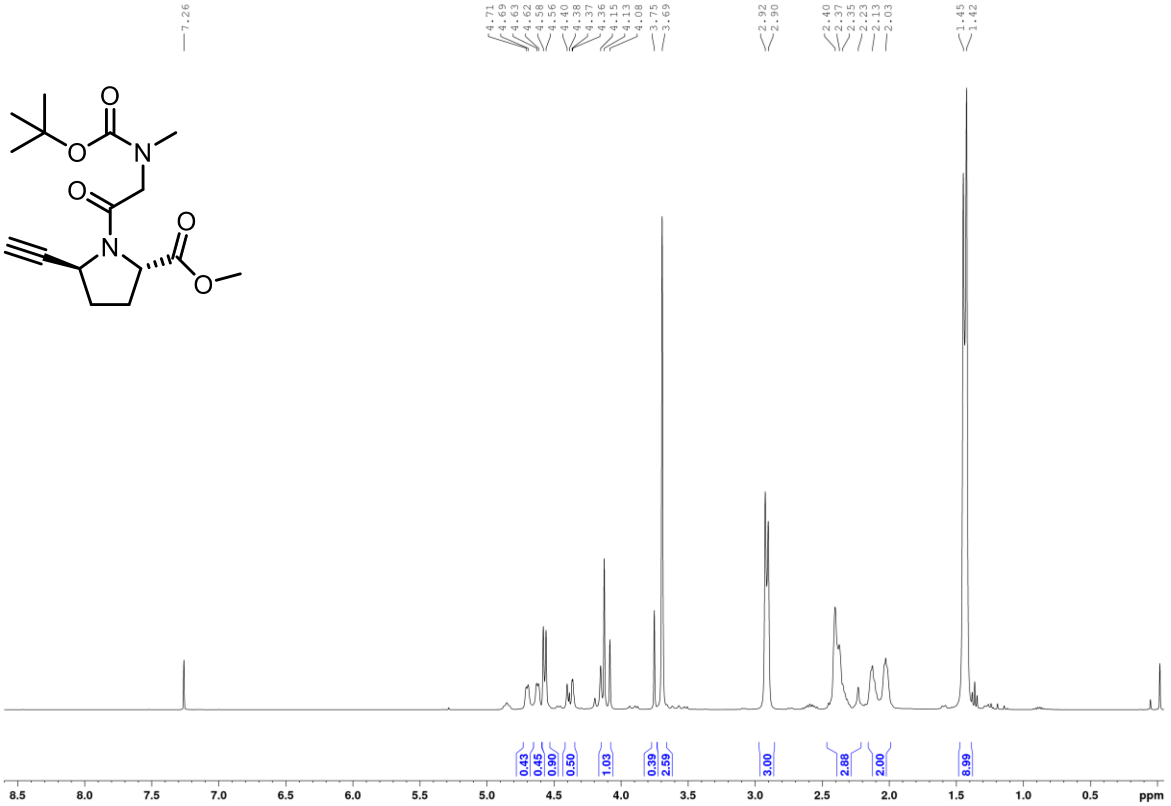

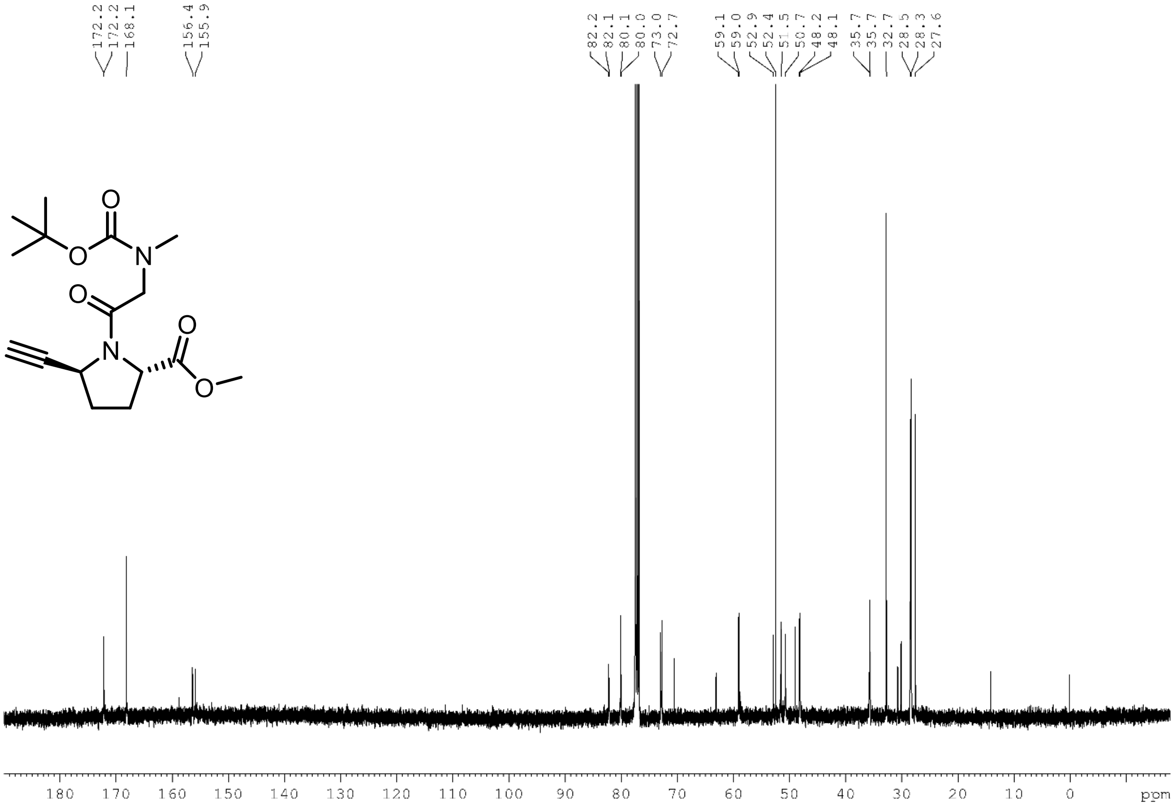

(6*S*,8a*S*)-6-Ethynyl-2-methylhexahydropyrrolo[1,2-*a*]pyrazine-1,4-dione (14)

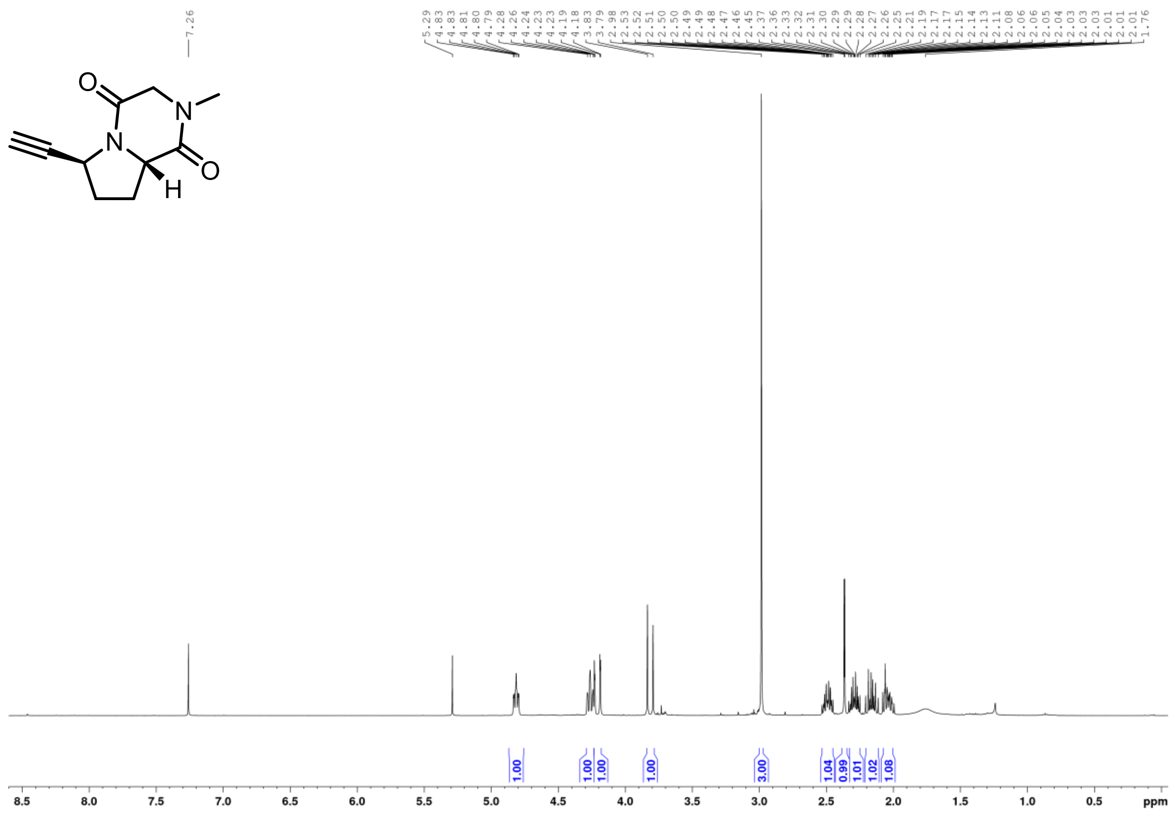

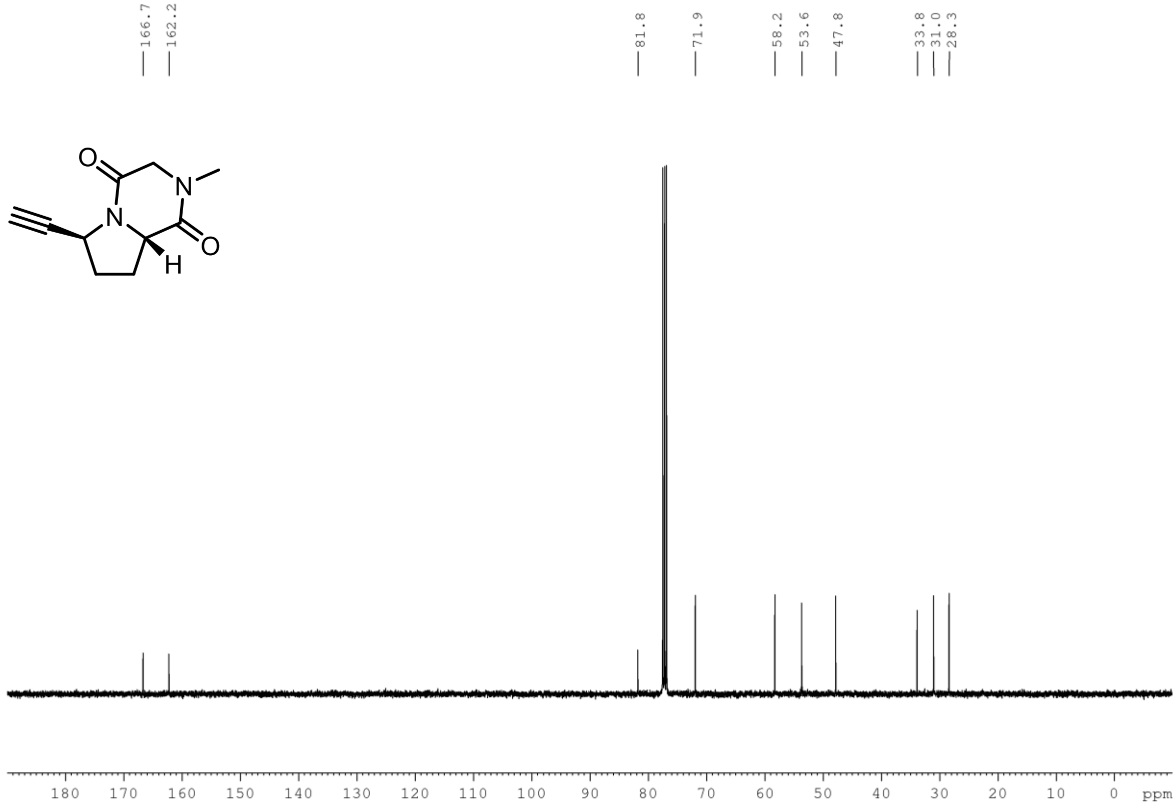

Methyl (2*S*,5*S*)-1-(but-3-enoyl)-5-ethynylpyrrolidine-2-carboxylate (S14)

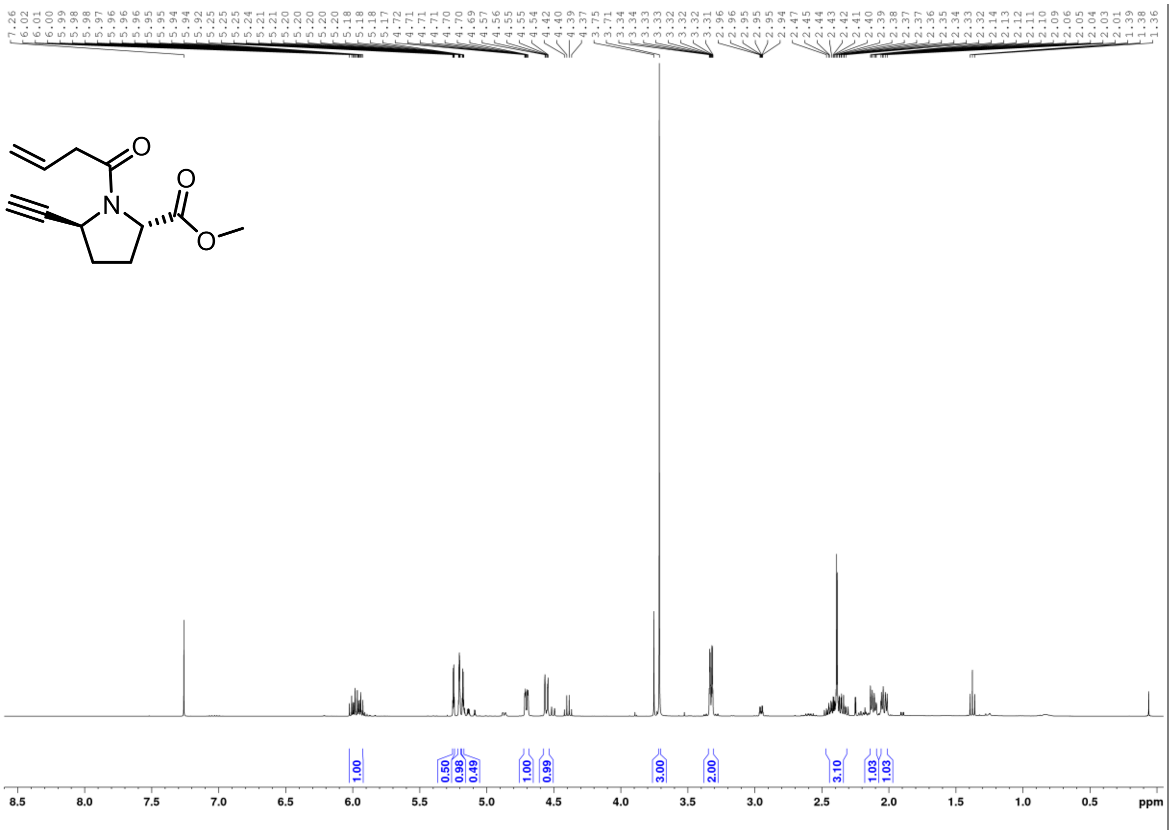

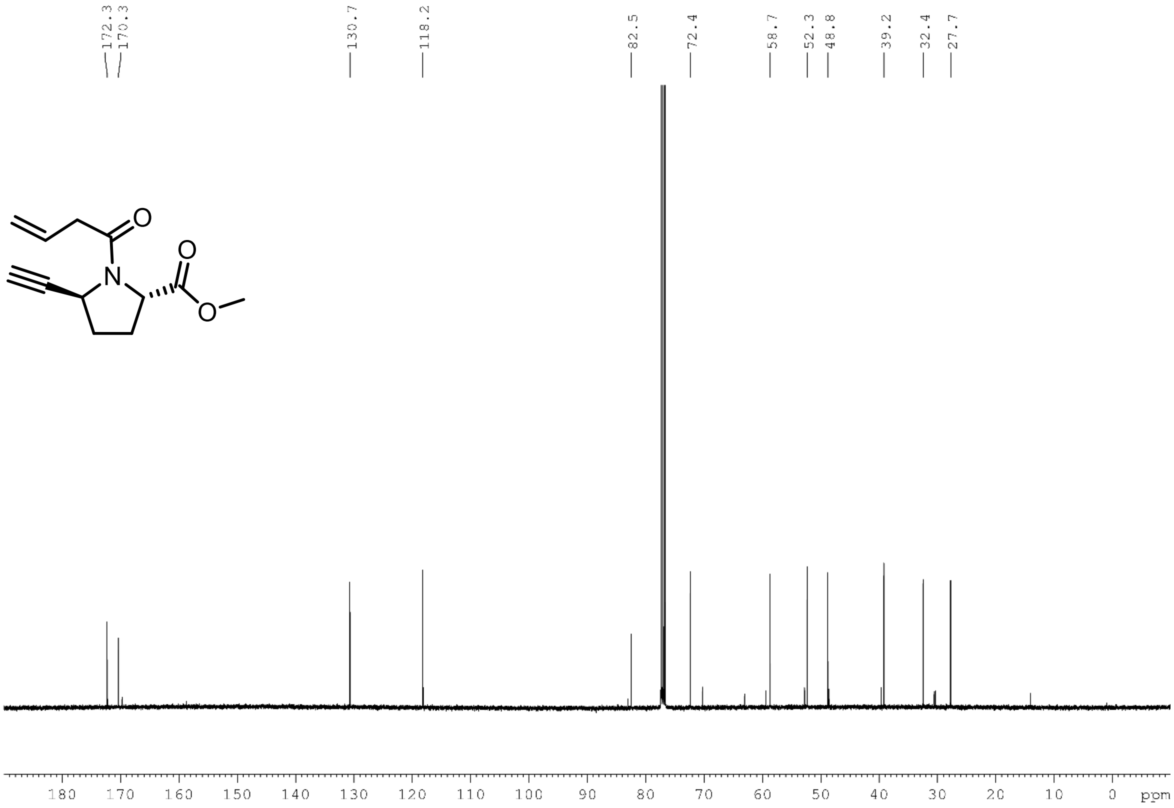

Methyl (3*S*,9b*S*)-5,8-dioxo-2,3,5,6,6a,7,8,9b-octahydro-1H-cyclopenta[*g*]indolizine-3-carboxylate (15)

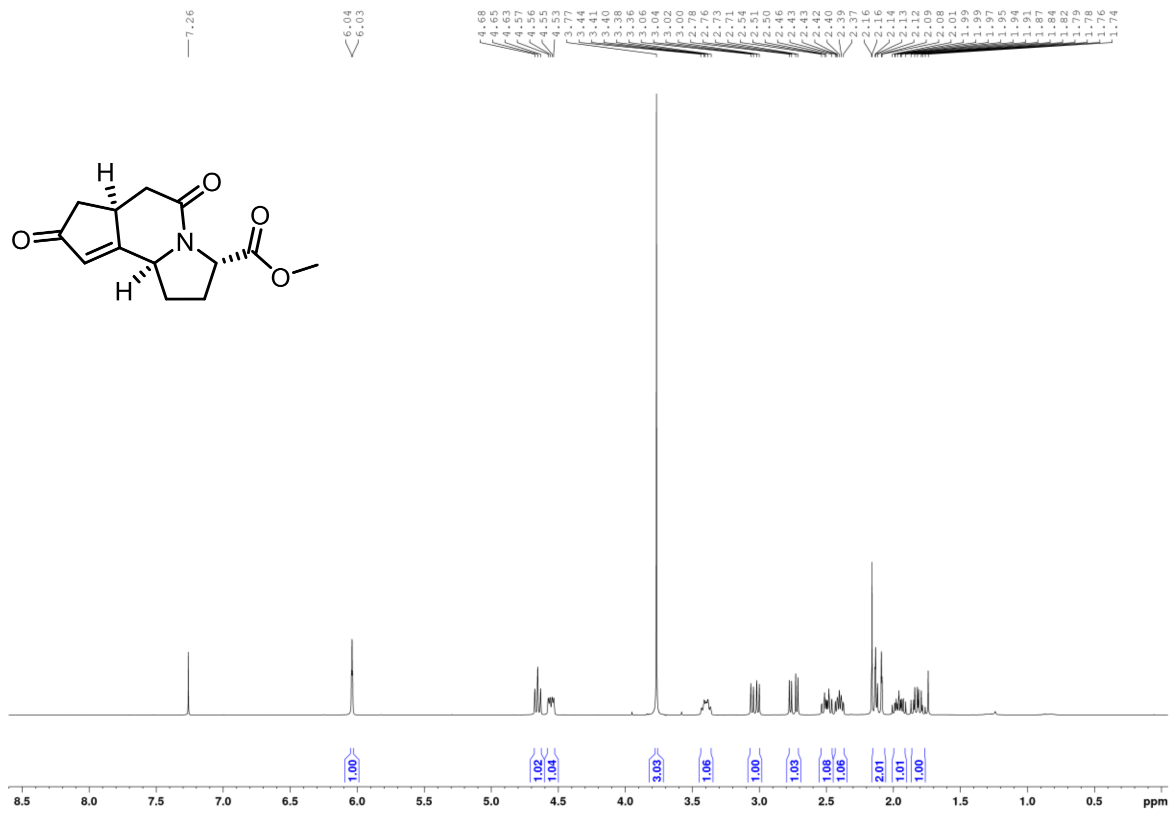

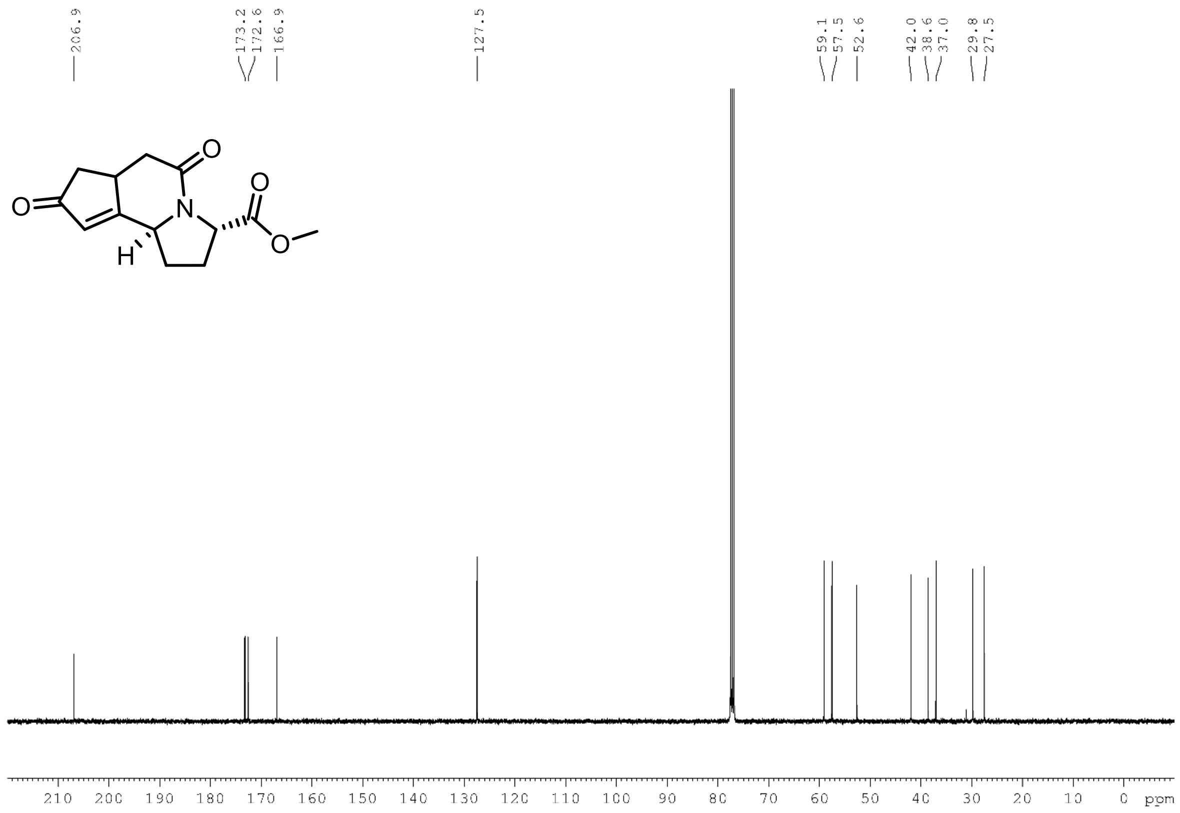

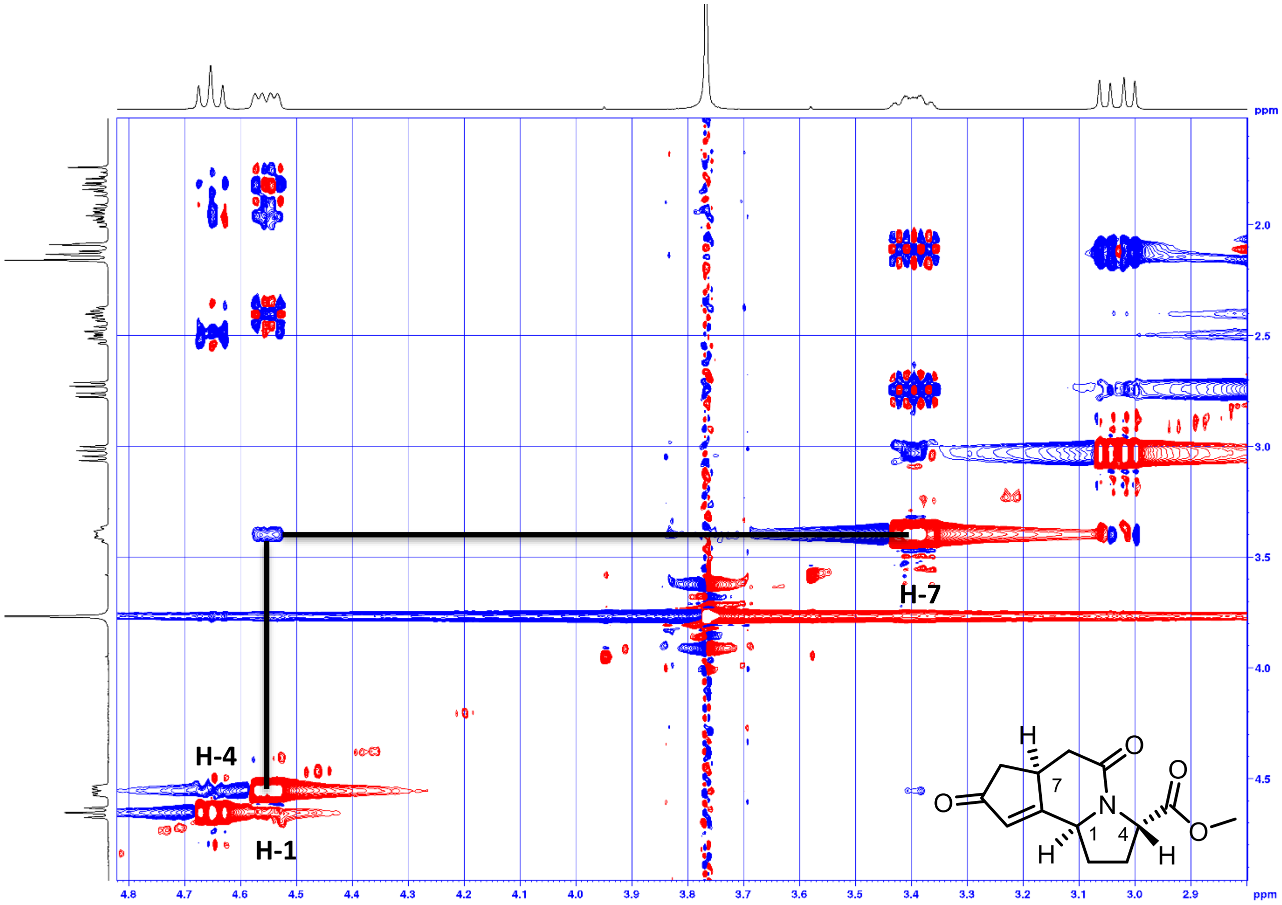

ppm
2.0
2.5
3.0
3.5
4.0
4.5
H-7
H-4
H-1
4.8
4.7
4.6
4.5
4.4
4.3
4.2
4.1
4.0
3.9
3.8
3.7
3.6
3.5
3.4
3.3
3.2
3.1
3.0
2.9
ppm
H
O
7
O
N
O
O
H
1
4
H

Methyl (8*S*,10a*S*)-6-oxo-5,6,8,9,10,10a-hexahydropyrrolo[1,2-*a*][1,2,3]triazolo[5,1-*c*]pyrazine-8-carboxylate (16)

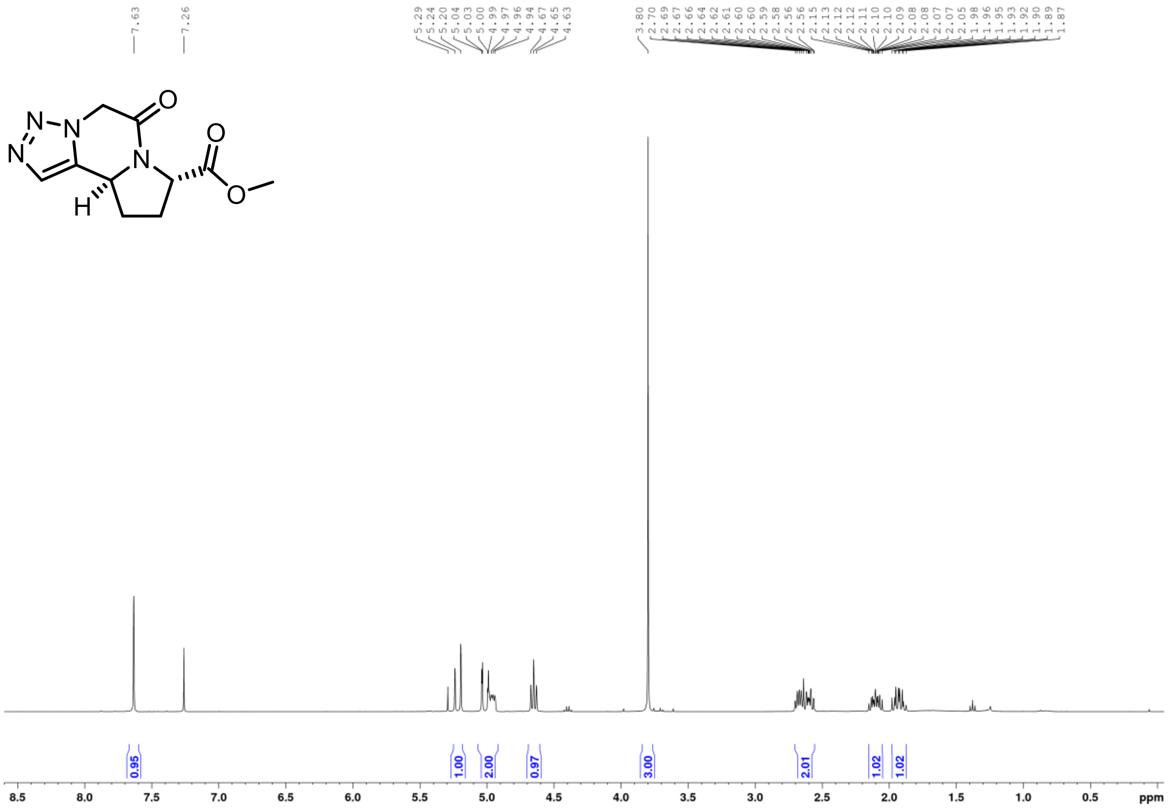

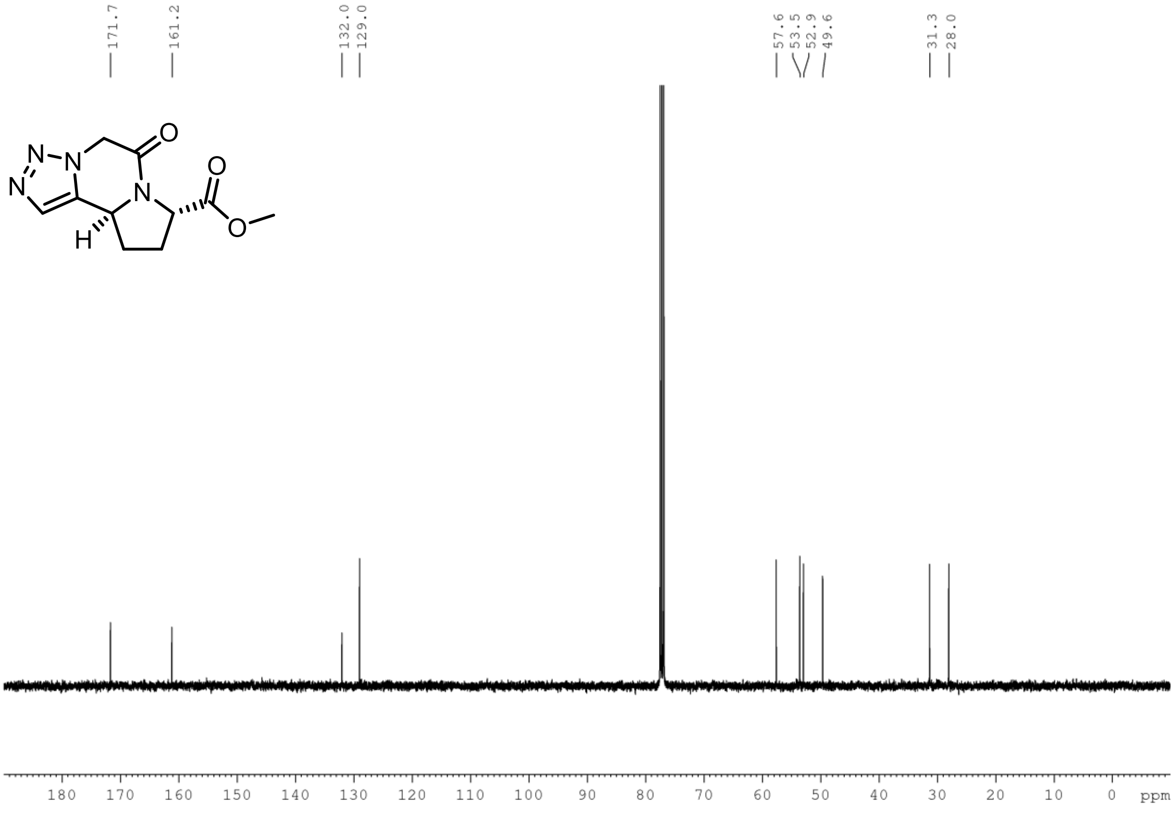

1-(*tert*-butyl) 2-Methyl (2*S*,5*S*)-5-(1-(2-ethoxy-2-oxoethyl)-1*H*-1,2,3-triazol-4-yl)pyrrolidine-1,2-dicarboxylate (17)

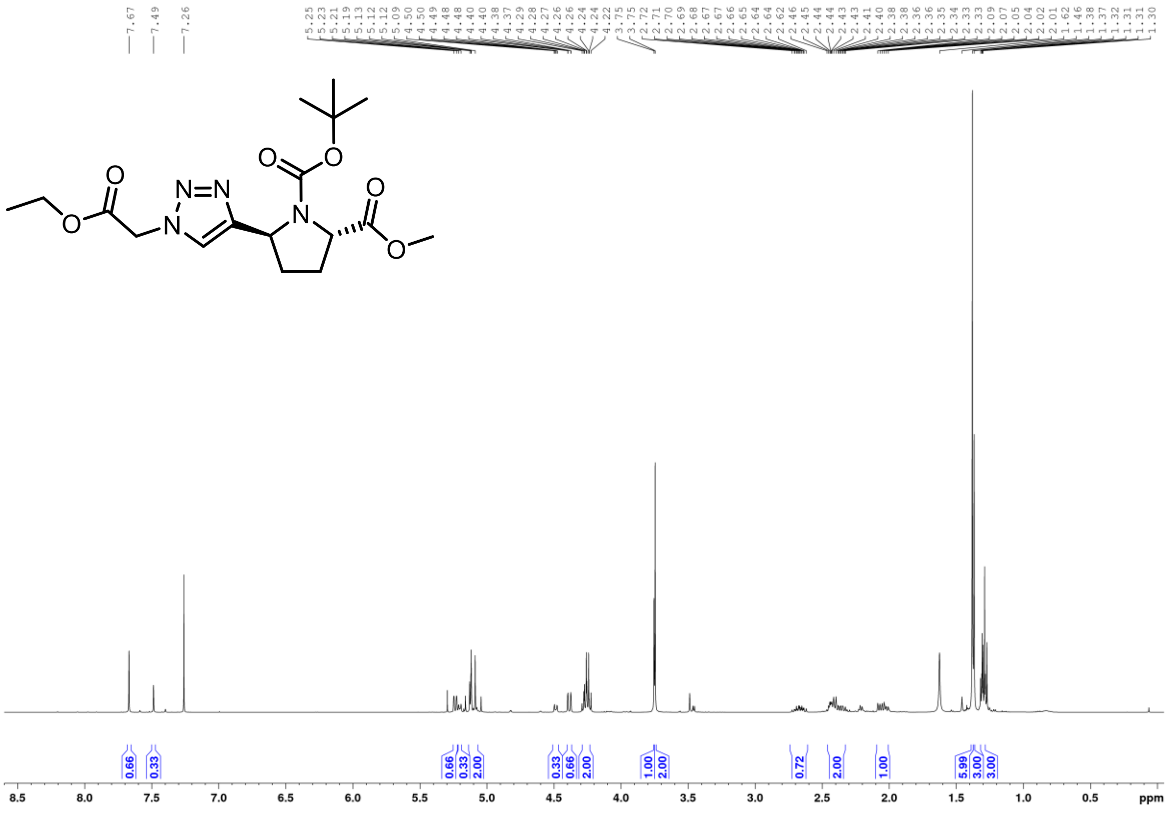

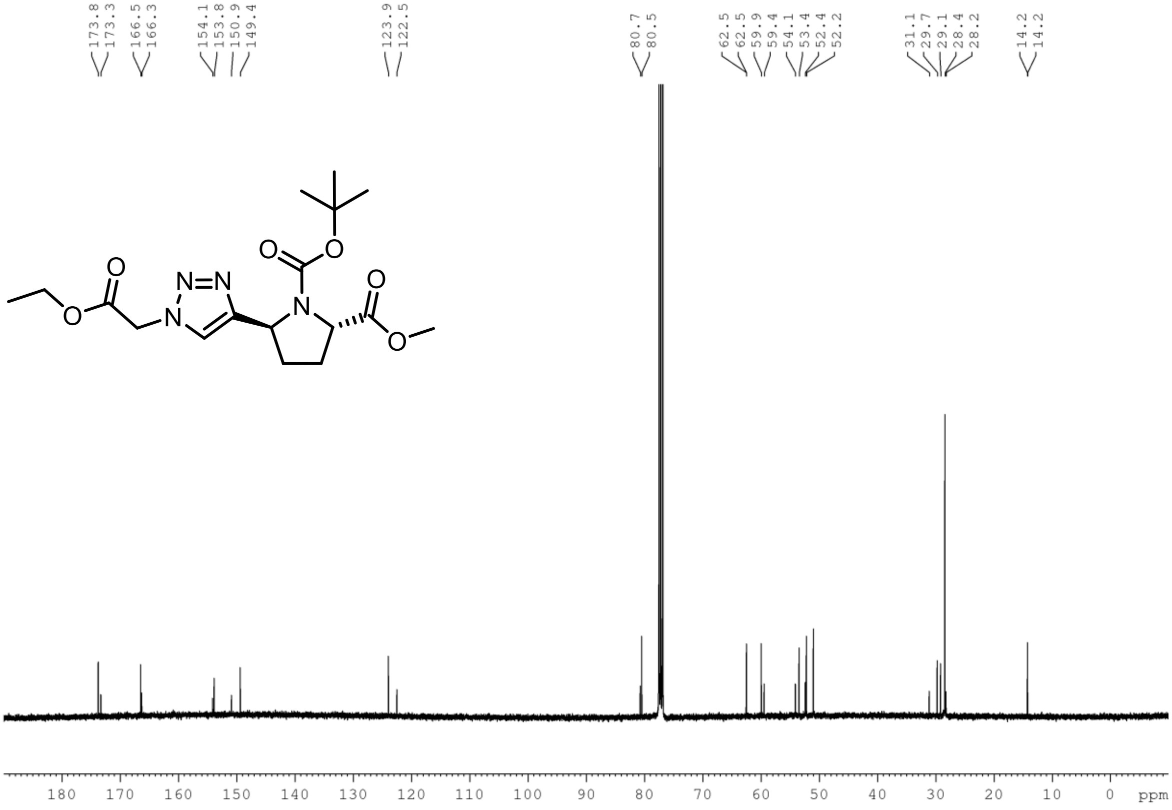

1-(*tert*-butyl) 2-Methyl (2*S*,5*S*)-5-((1-(tert-butoxycarbonyl)-1,2,3,6-tetrahydropyridin-4-yl)ethynyl)pyrrolidine-1,2-dicarboxylate (18)

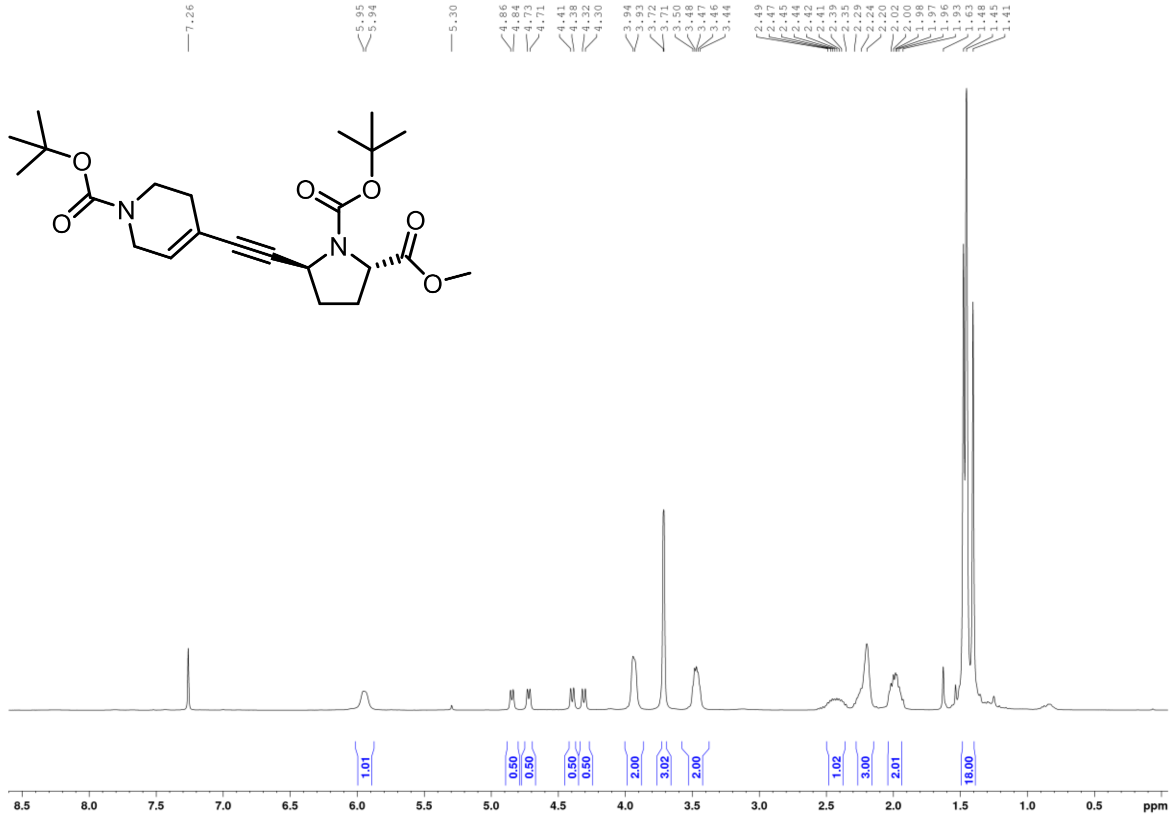

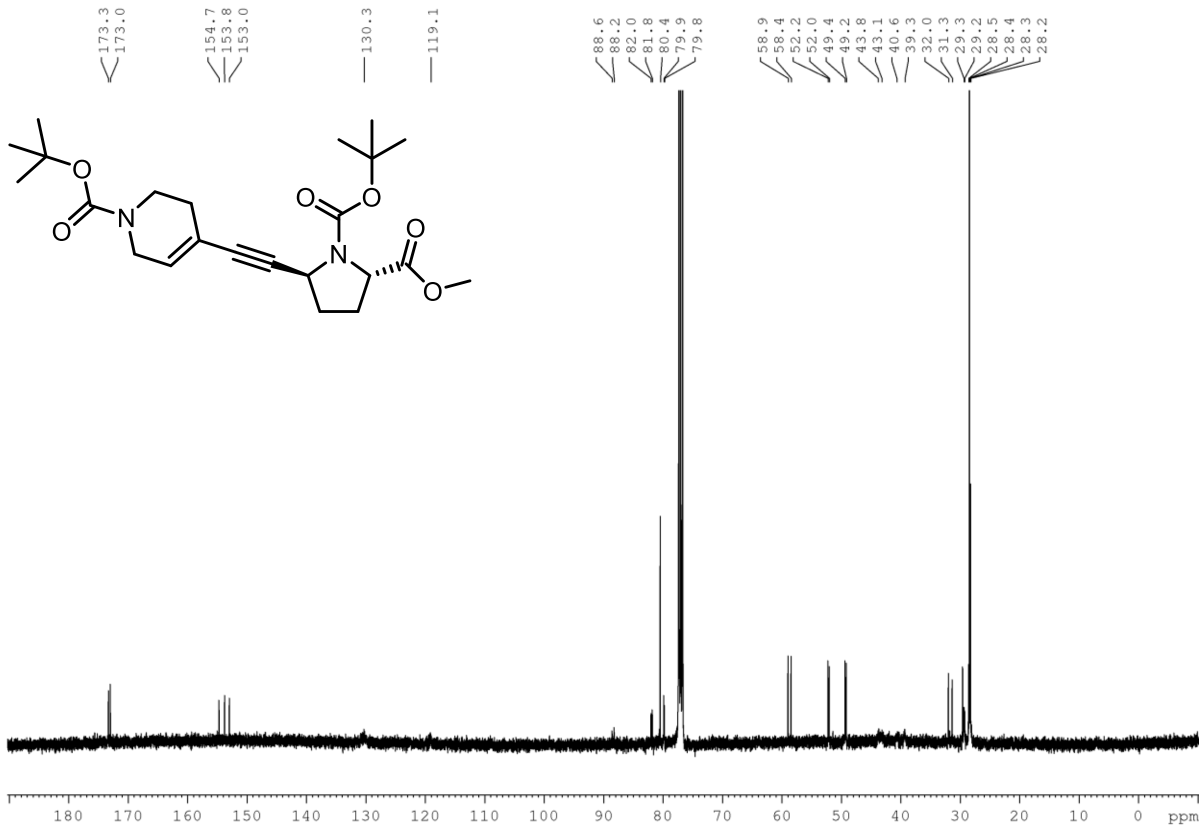

(*S*)-5-(Hydroxymethyl)-1-(4-methoxyphenyl)pyrrolidin-2-one (S16)

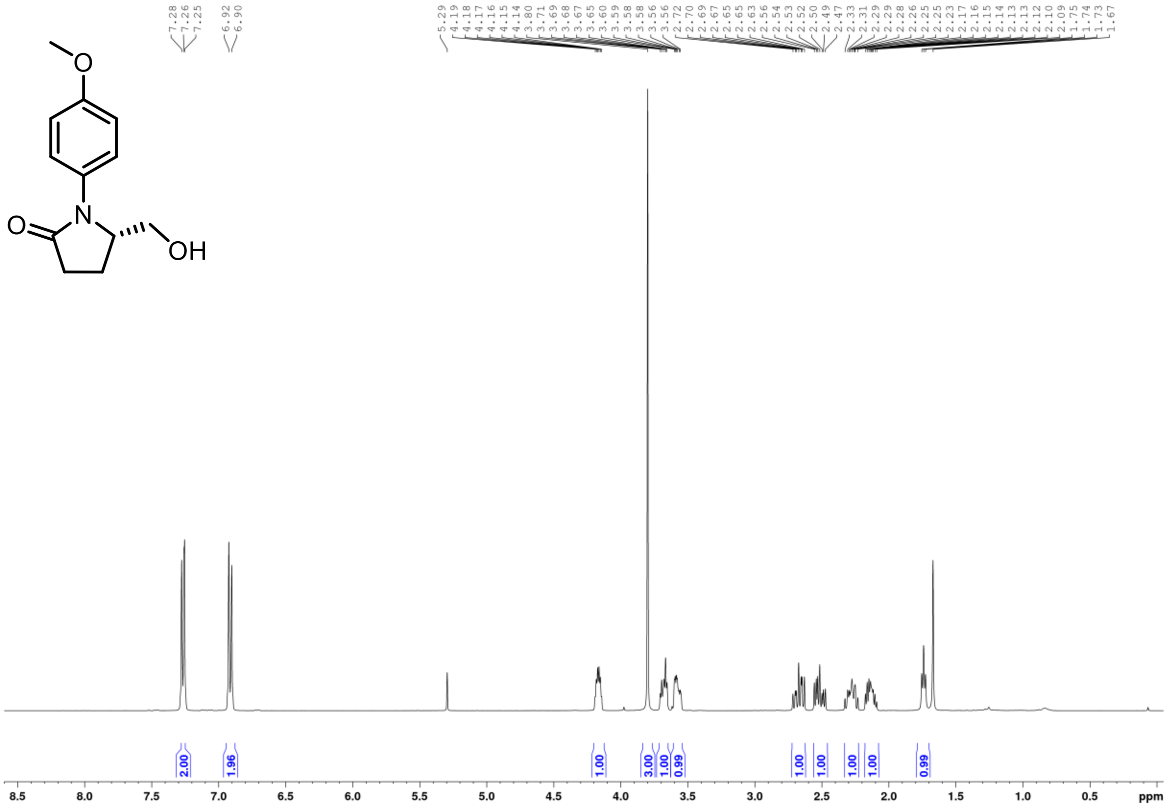

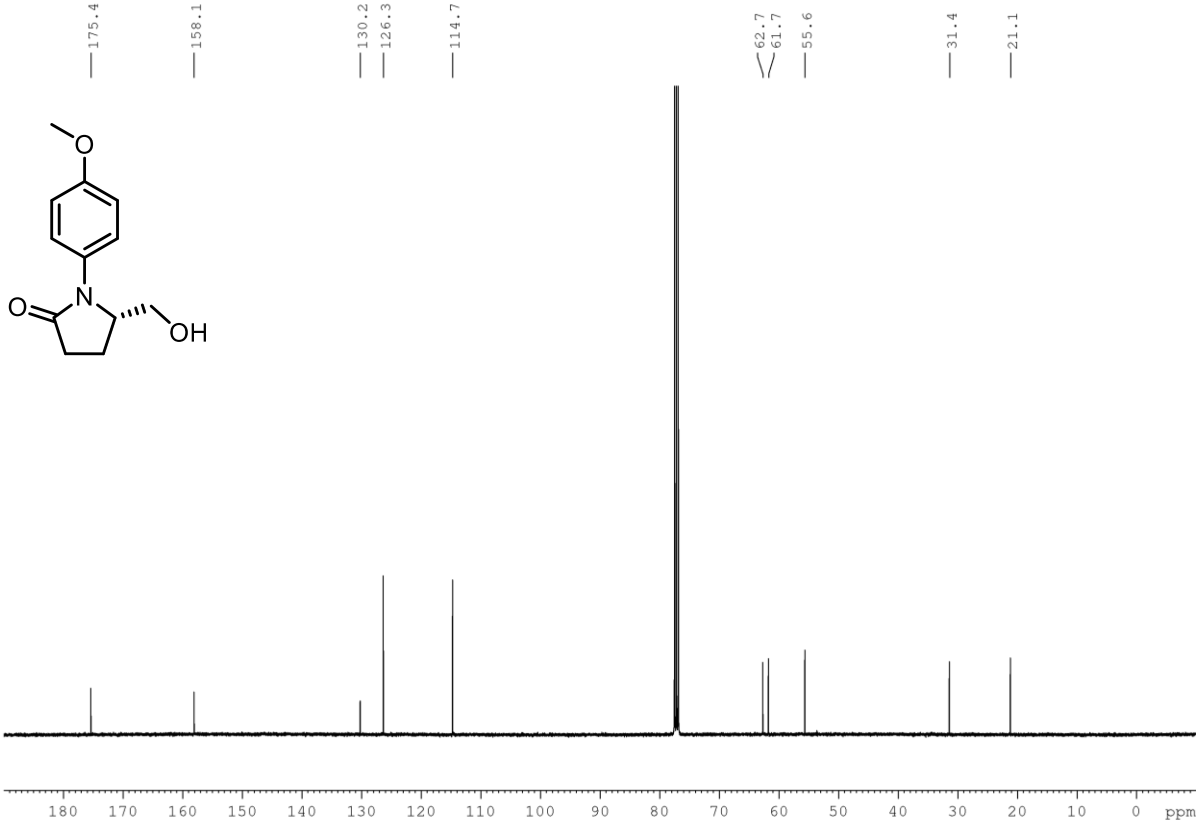

(*S*)-1',6',7',7a'-Tetrahydro-5'*H*-spiro[cyclohexane-1,3'-pyrrolo[1,2-*c*]oxazole]-2,5-diene-4,5'-dione (19)

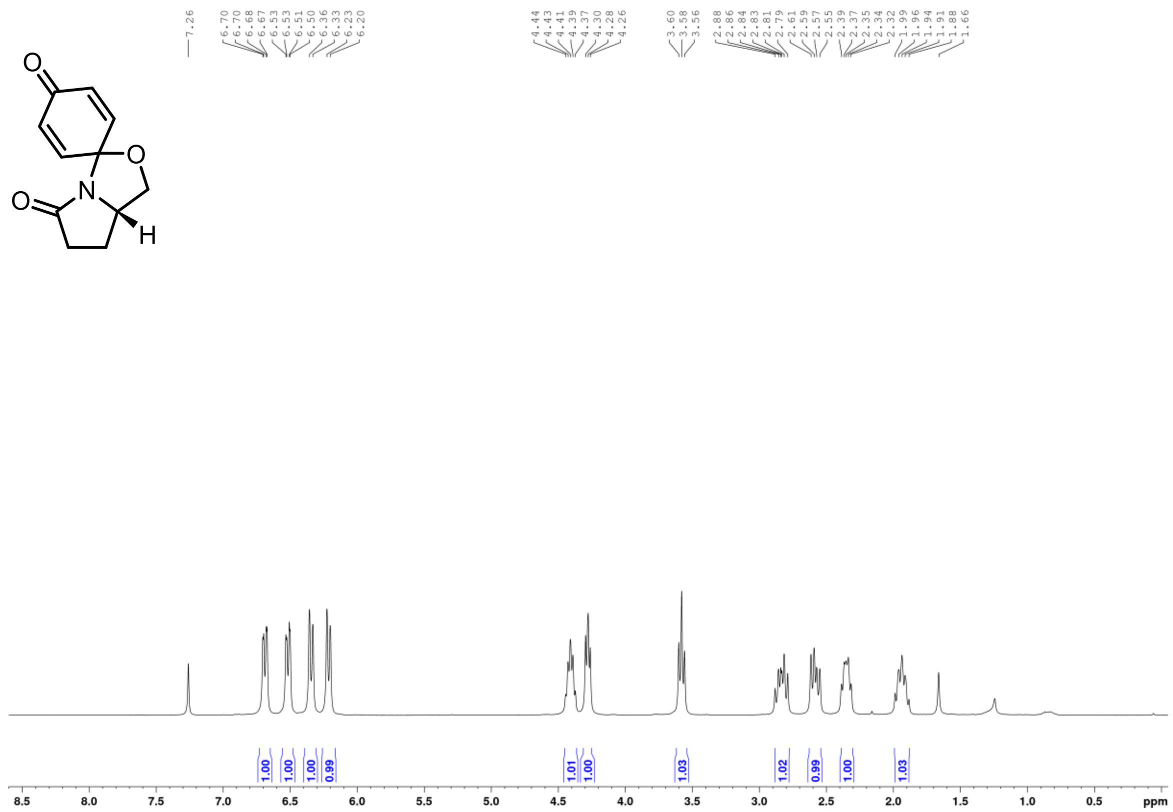

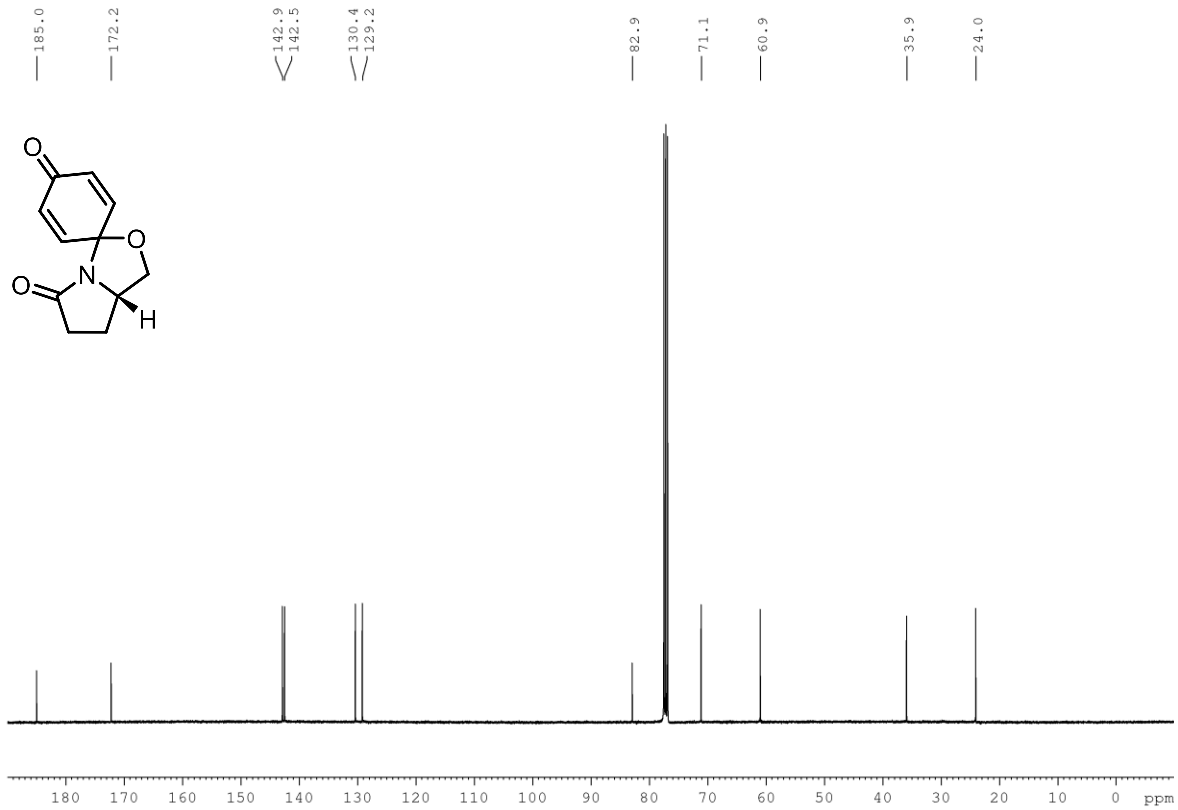

(*S*)-1-Allyl-5-((allyloxy)methyl)pyrrolidin-2-one (S17)

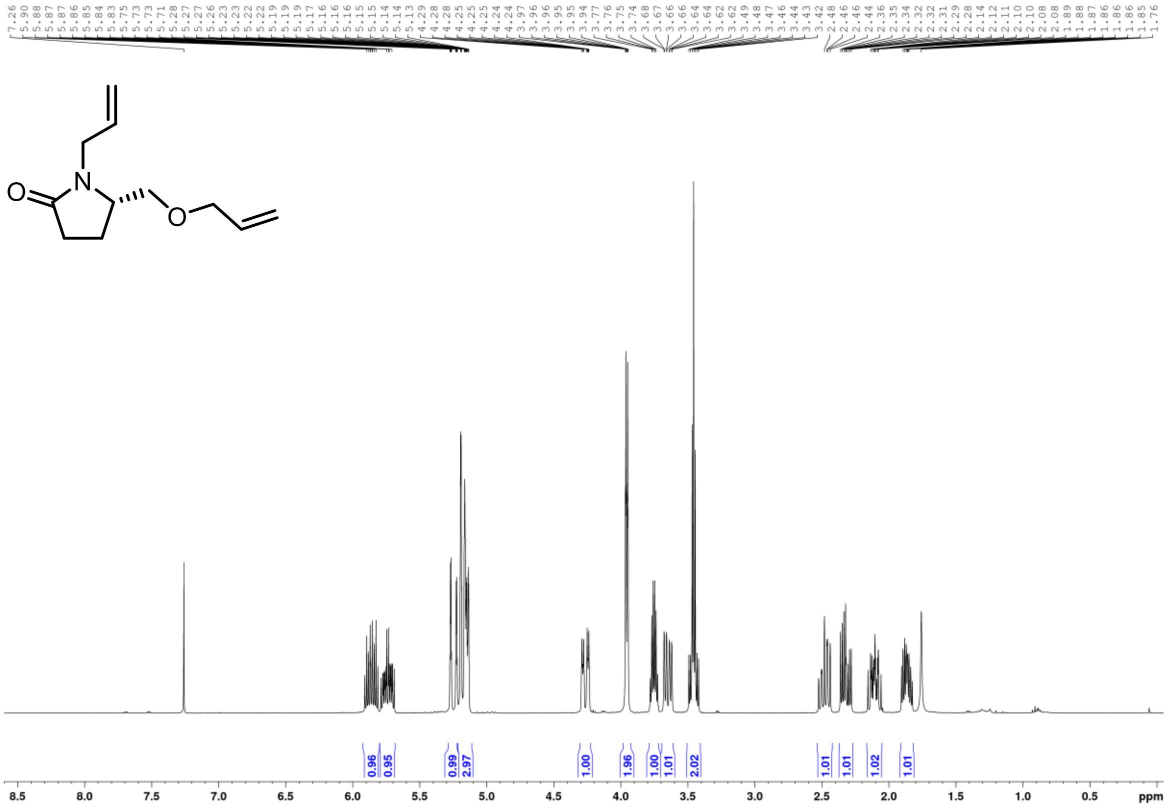

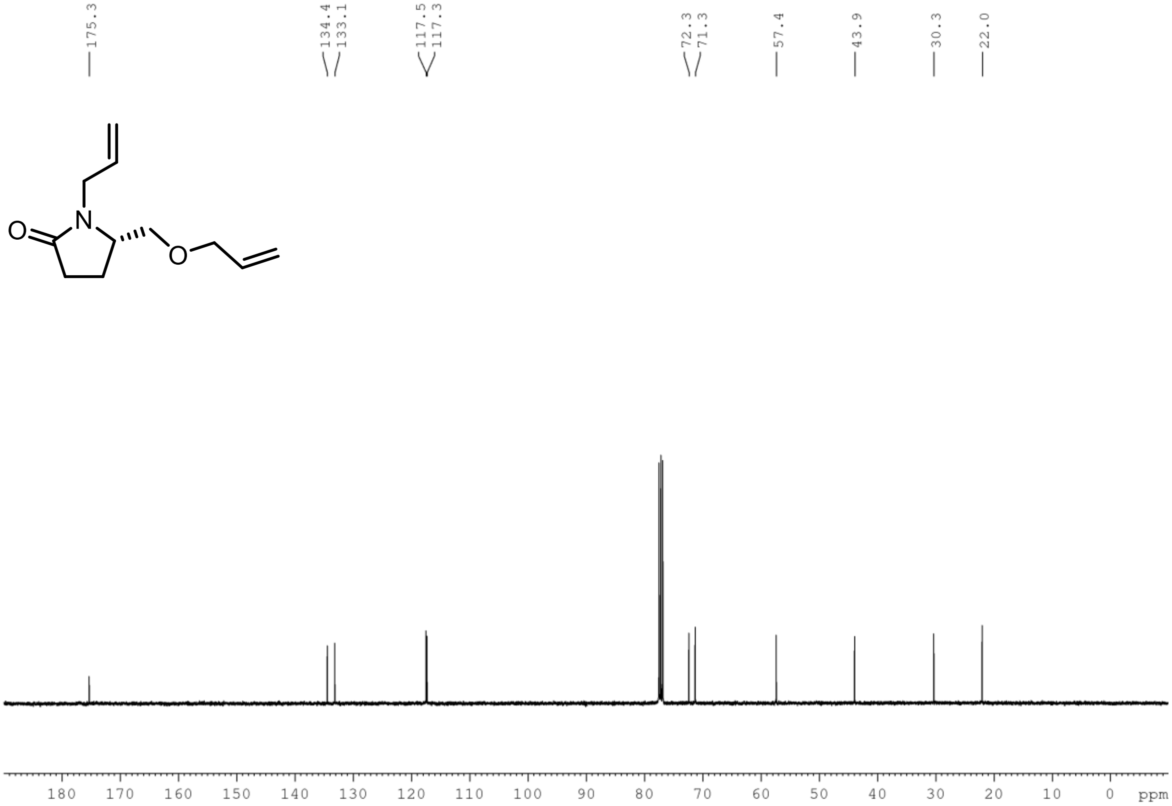

(*S*,*Z*)-1,3,6,9,10,10a-hexahydro-8*H*-pyrrolo[2,1-*c*][1,4]oxazocin-8-one (20)

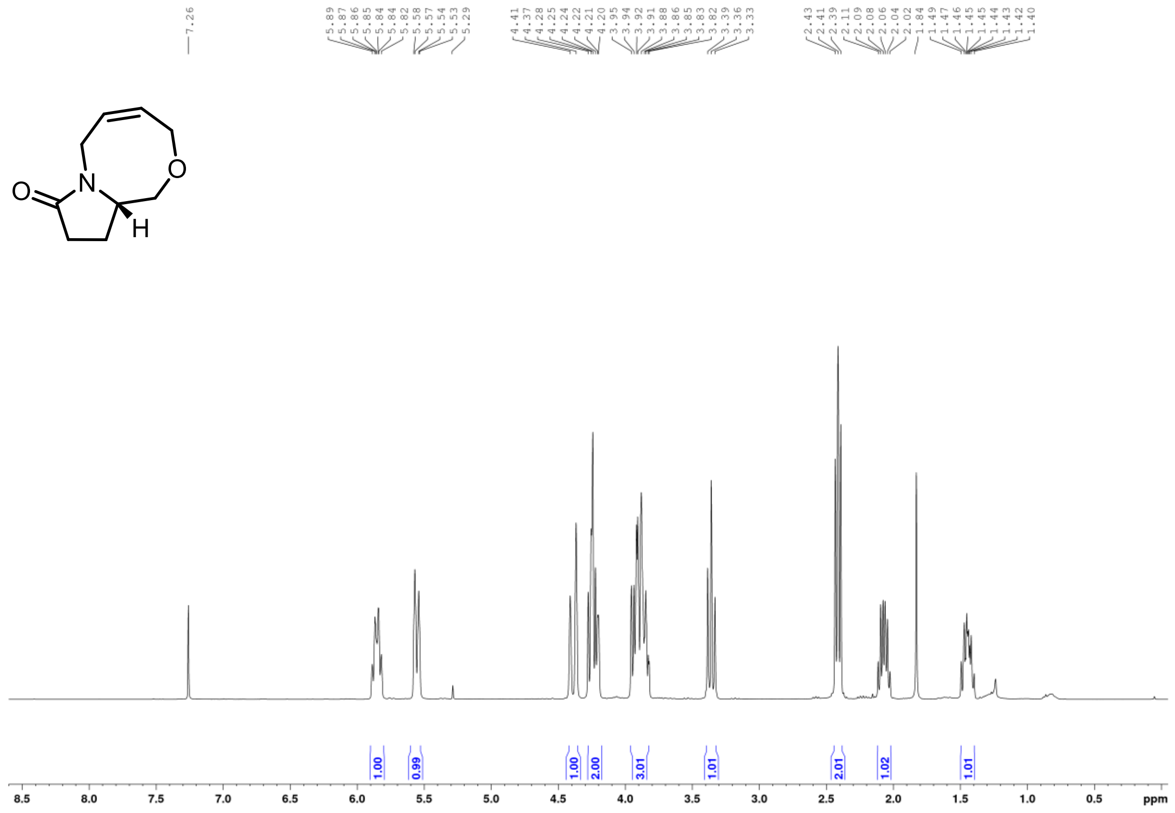

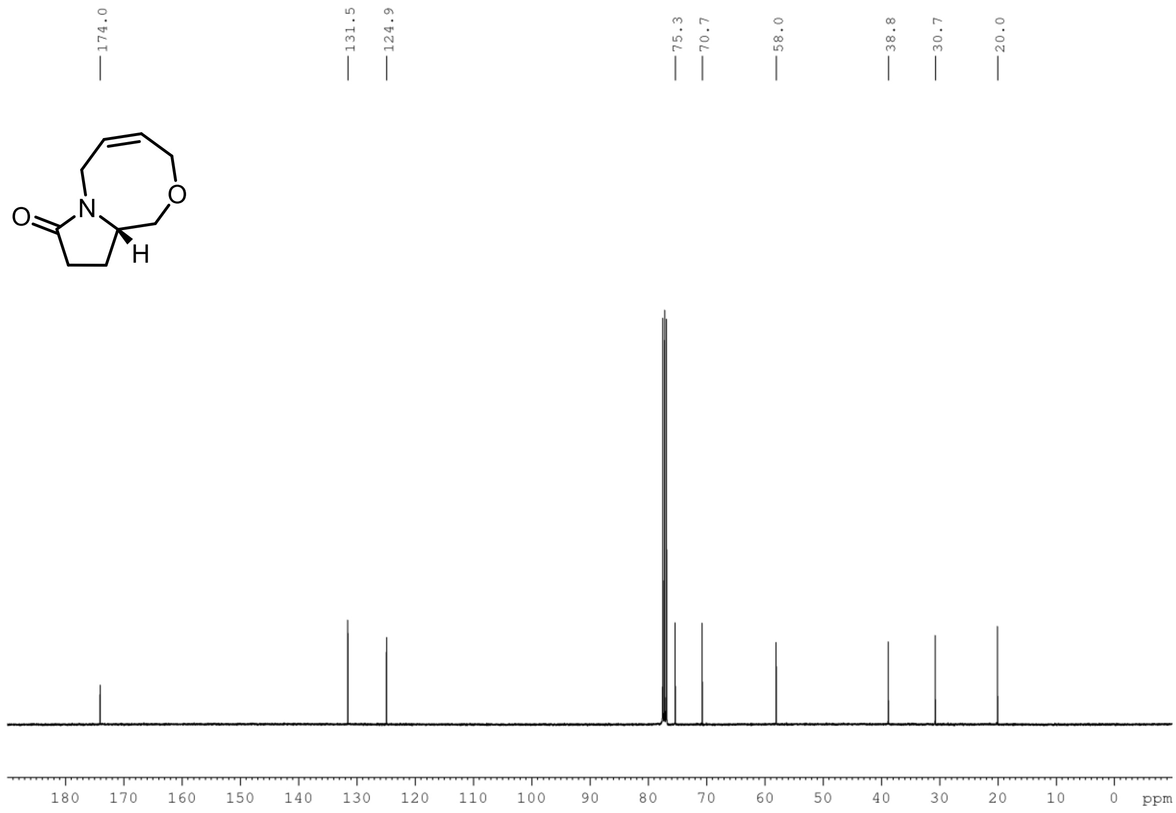

Methyl 2-(2-cyanoethyl)-5-phenyl-3,4-dihydro-2*H*-pyrrole-2-carboxylate (S18)

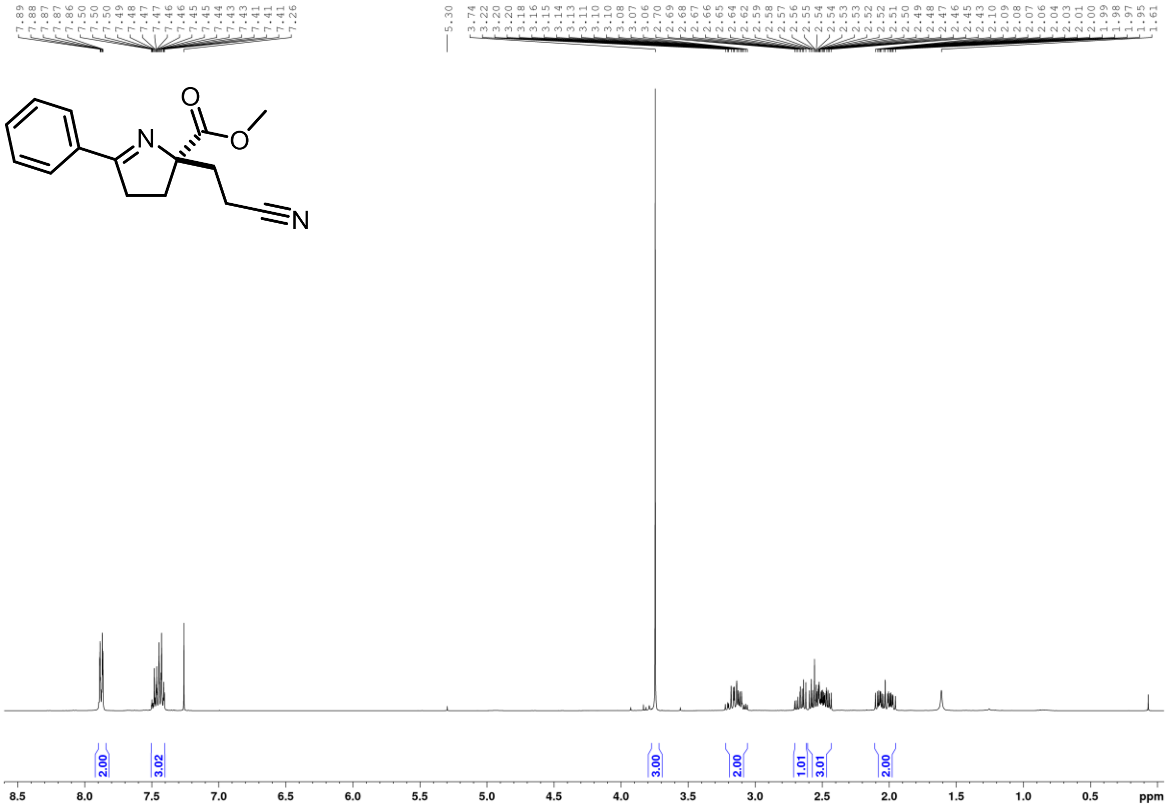

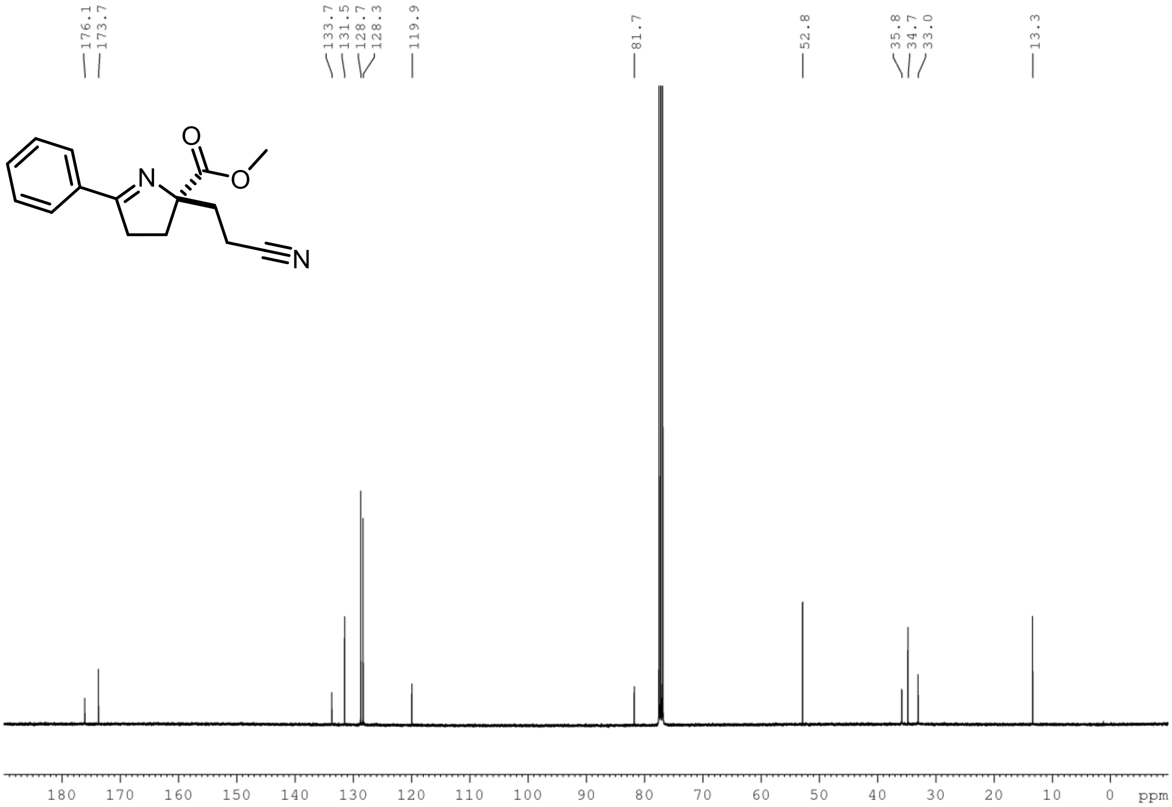

2-Phenyl-1,7-diazaspiro[4.5]dec-1-en-6-one (21)

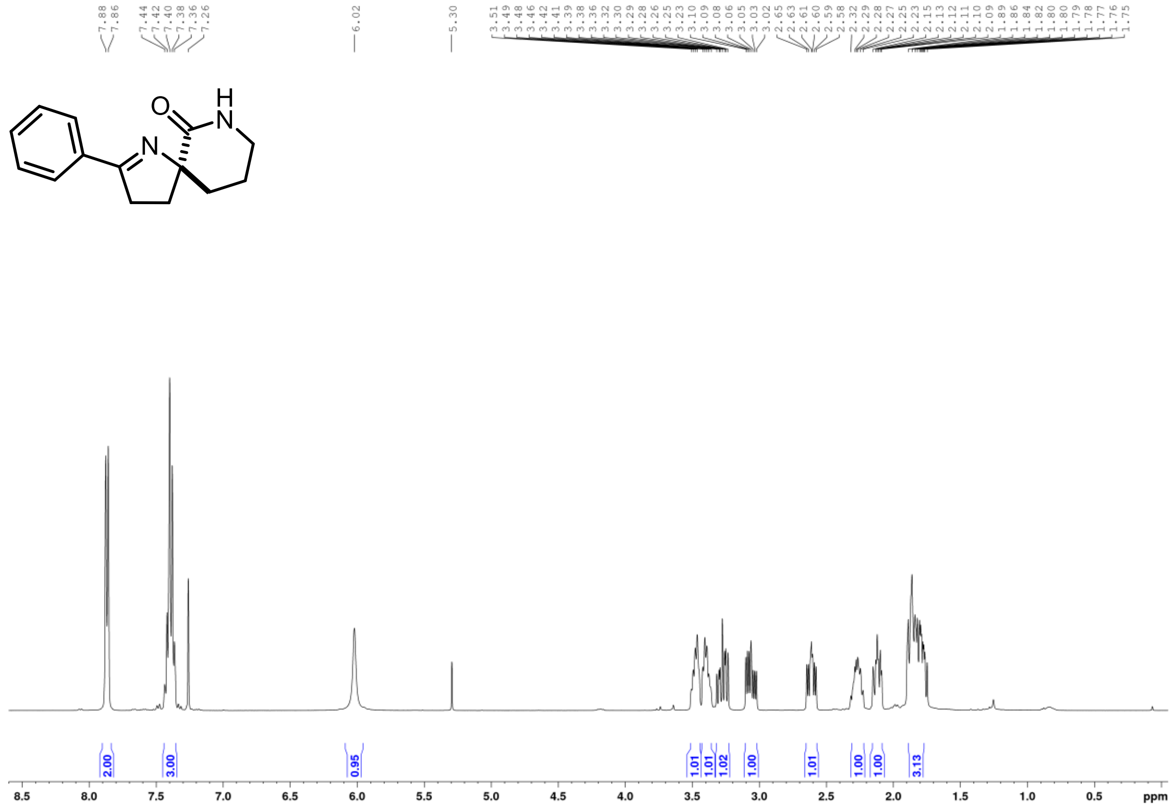

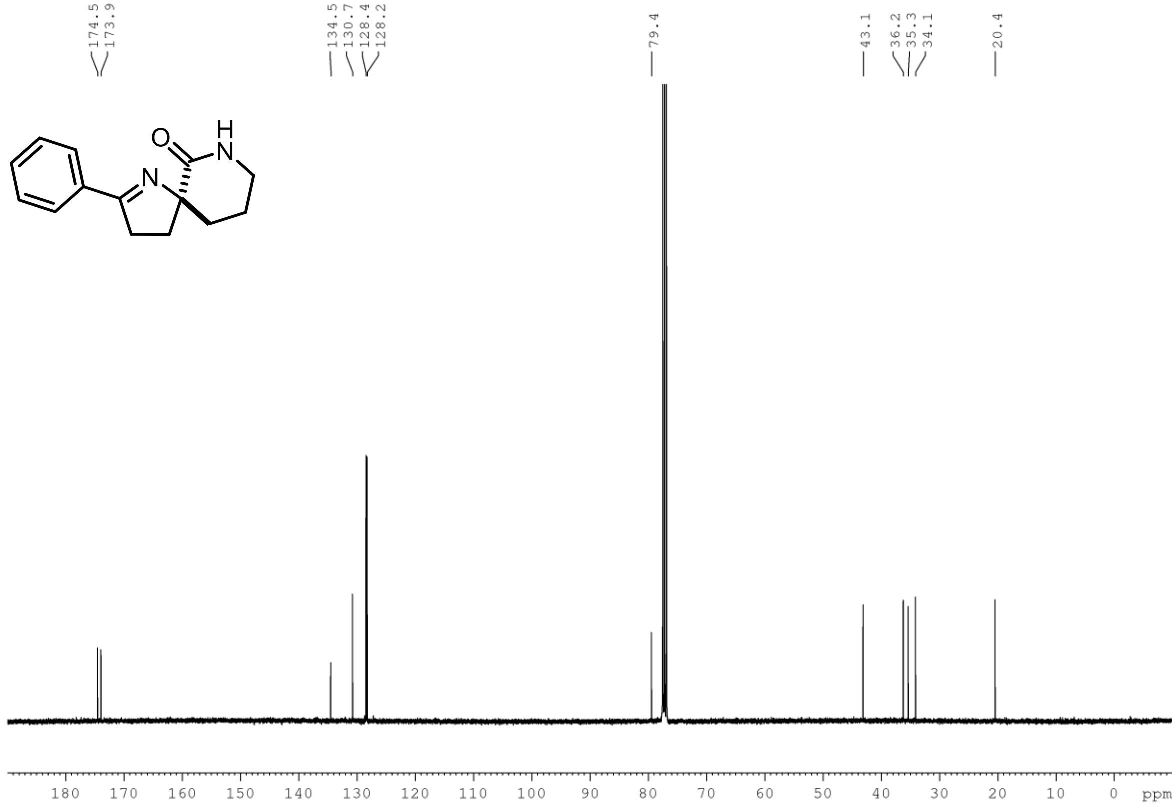

Methyl 2-(2-nitro-1-phenylethyl)-5-phenyl-3,4-dihydro-2*H*-pyrrole-2-carboxylate (S19)

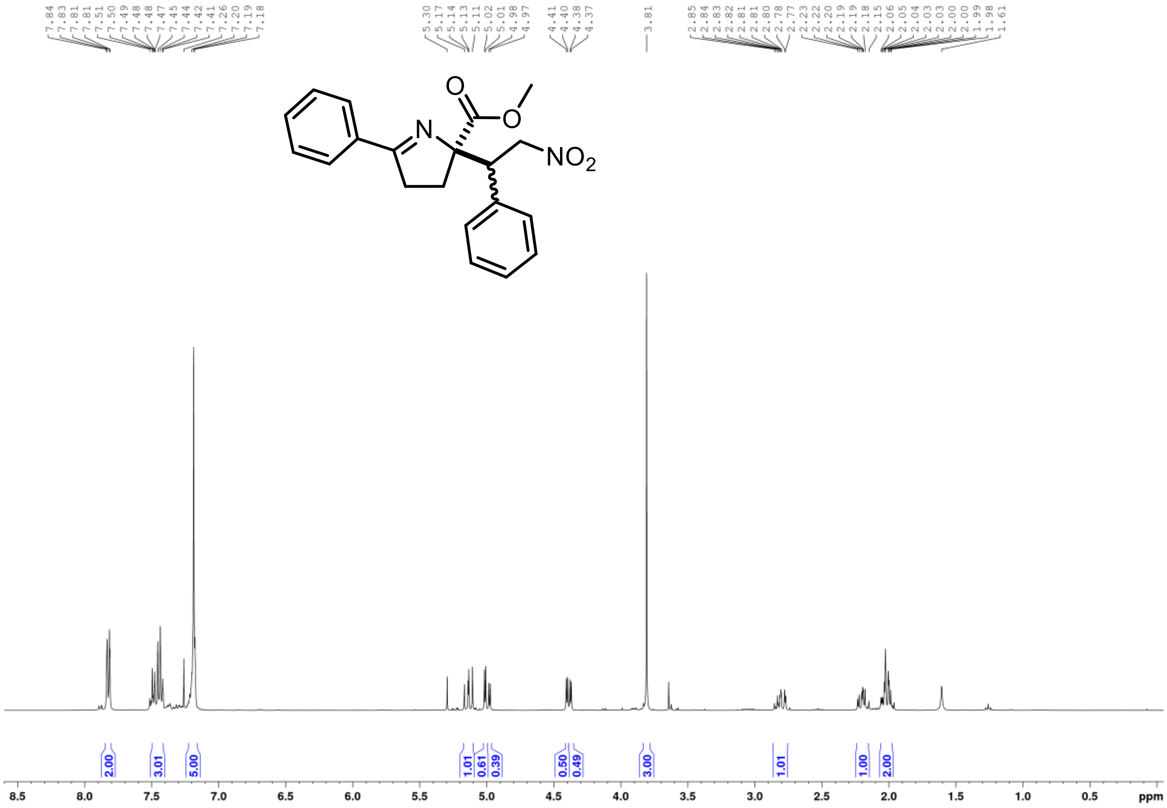

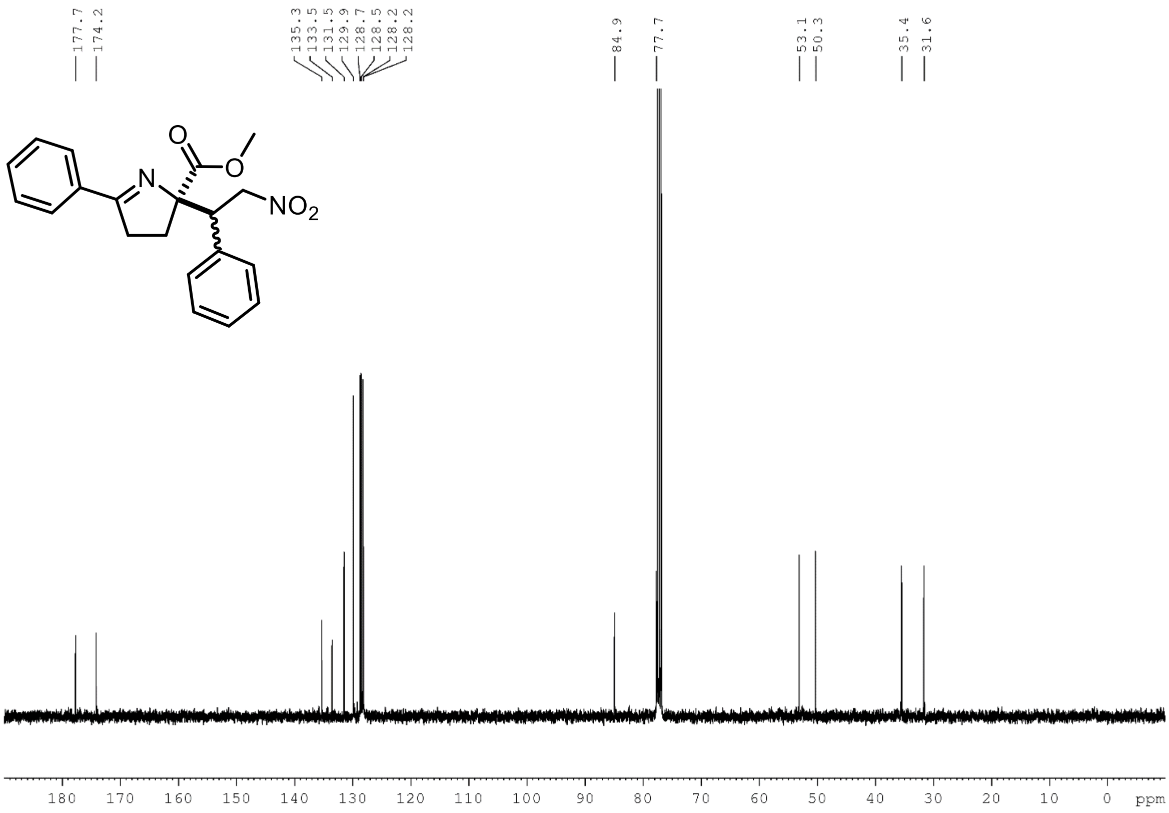

2,9-Diphenyl-1,7-diazaspiro[4.4]non-1-en-6-one (22)

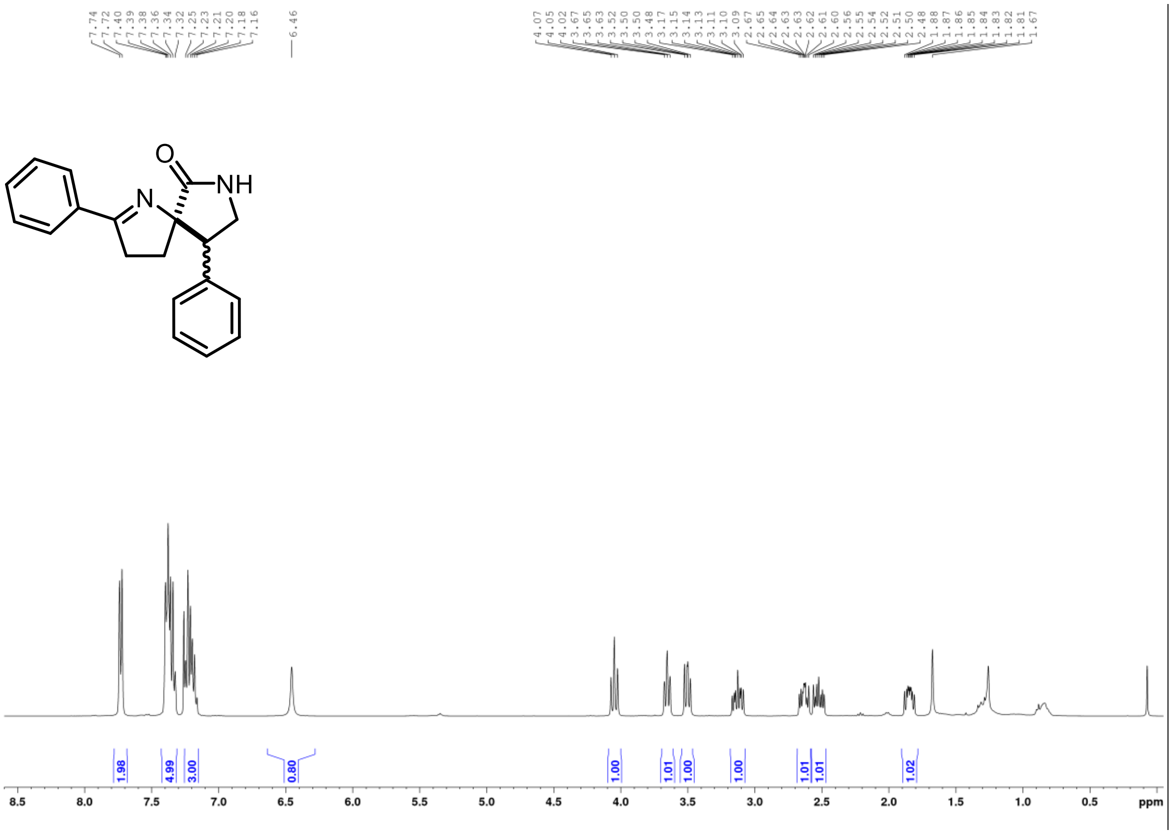

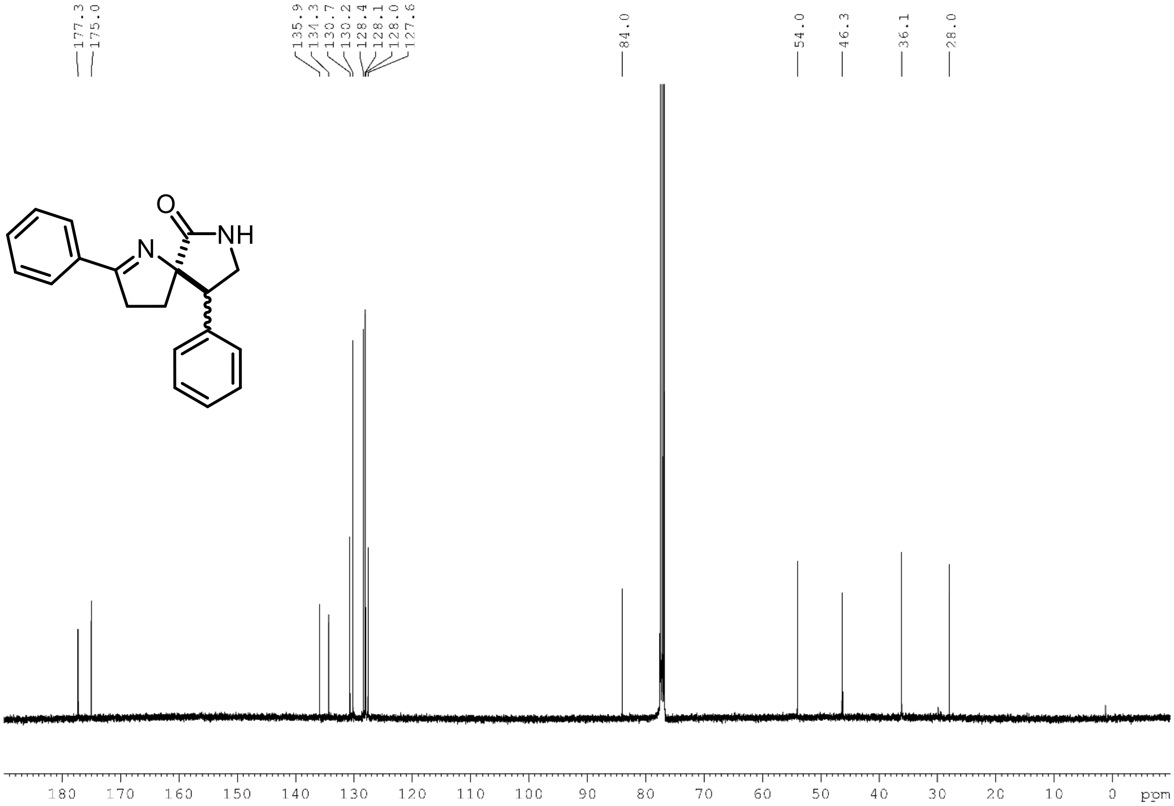

Methyl 2-(3-methoxy-3-oxopropyl)-5-phenyl-3,4-dihydro-2*H*-pyrrole-2-carboxylate (S20)

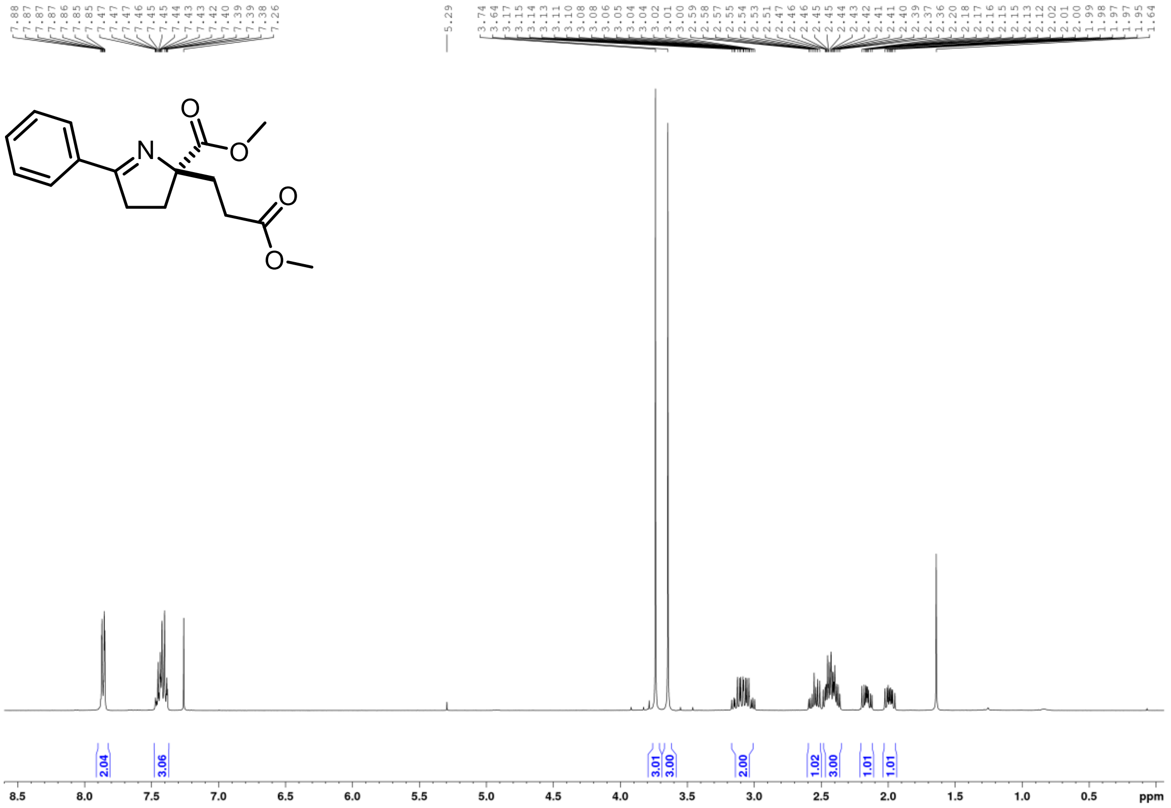

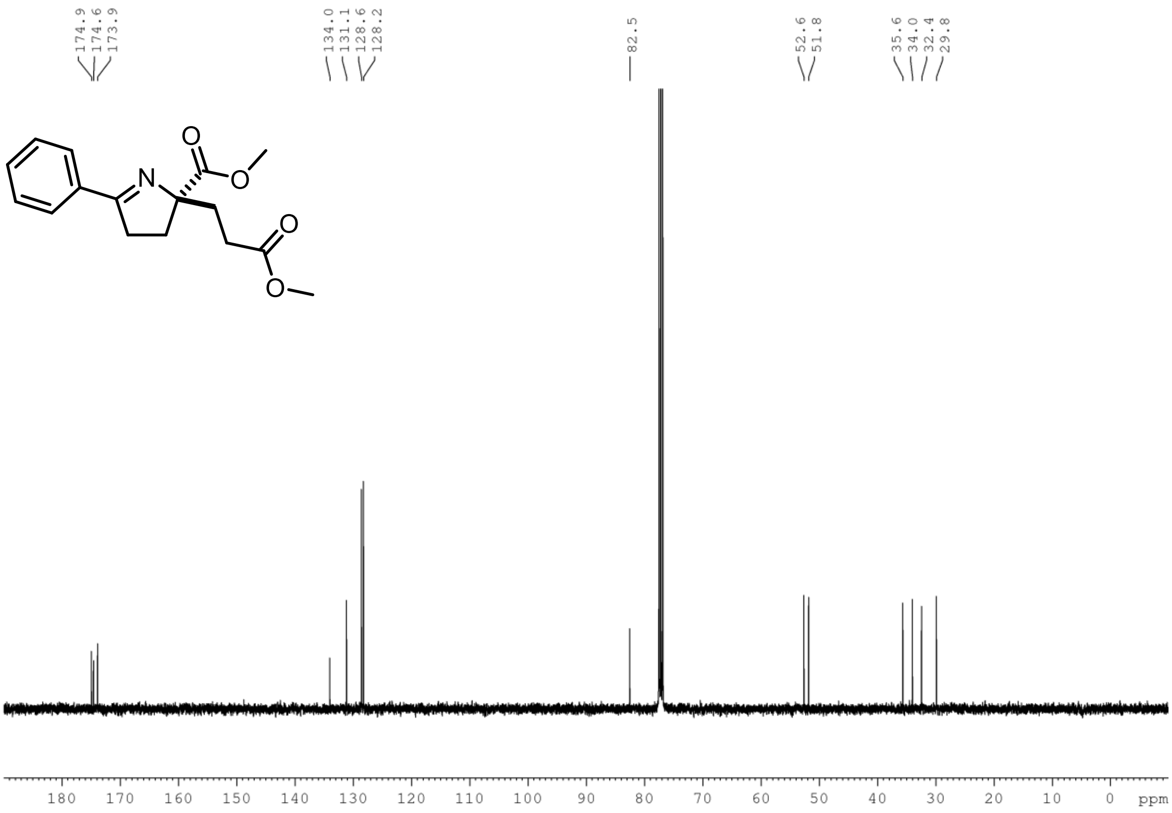

Methyl 3-oxo-5-phenyltetrahydro-1*H*-pyrrolizine-7a(5*H*)-carboxylate (23a)

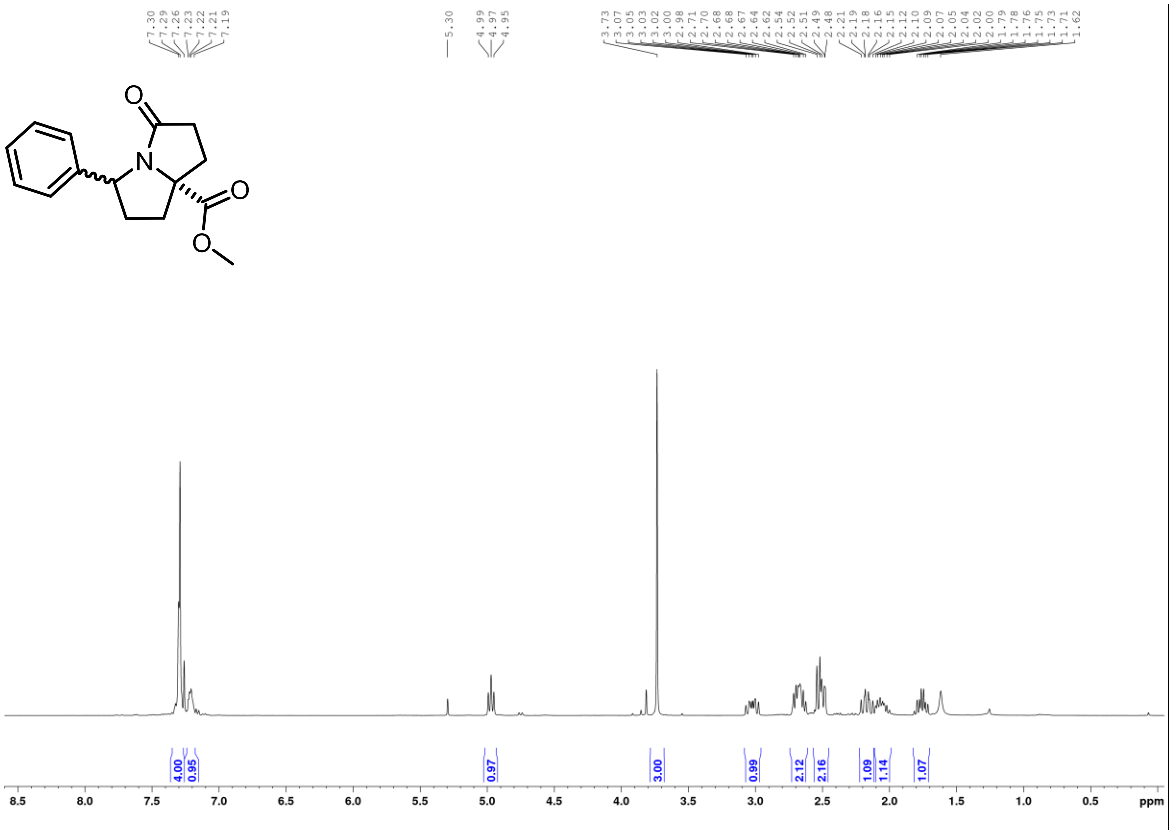

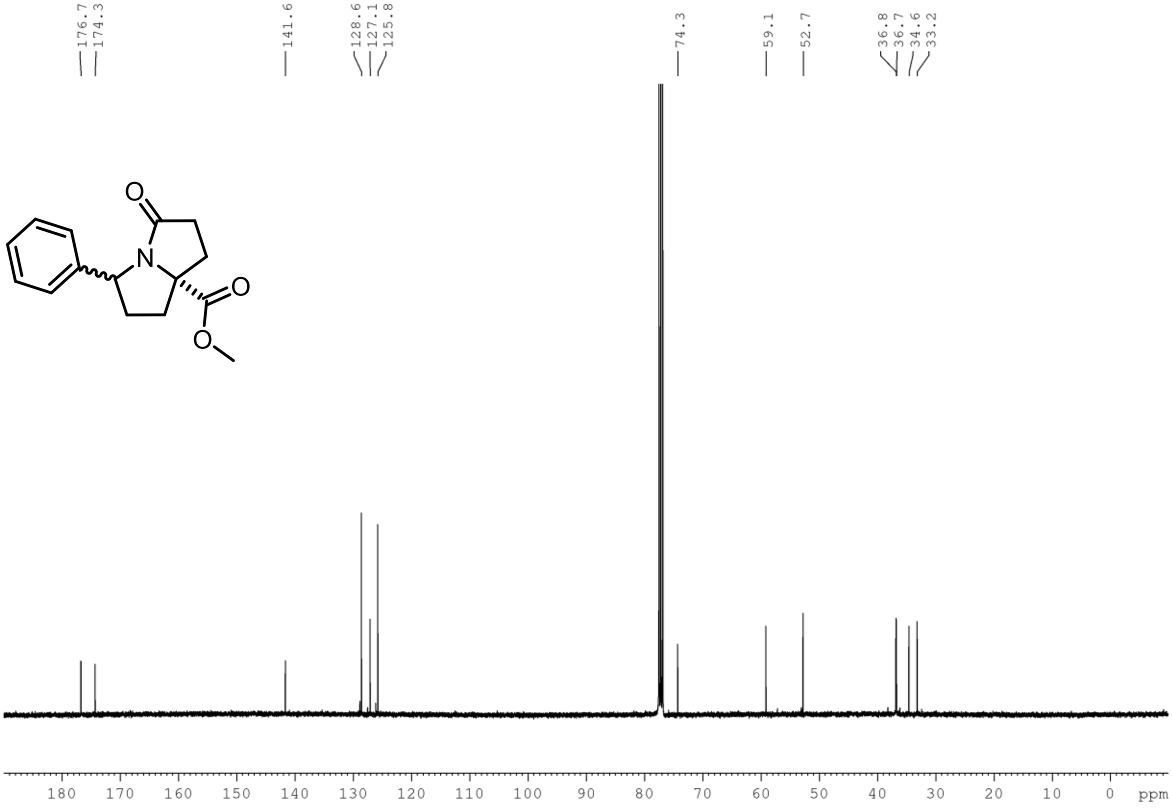

Methyl 2-(3-methoxy-3-oxopropyl)-5-phenylpyrrolidine-2-carboxylate (23b)

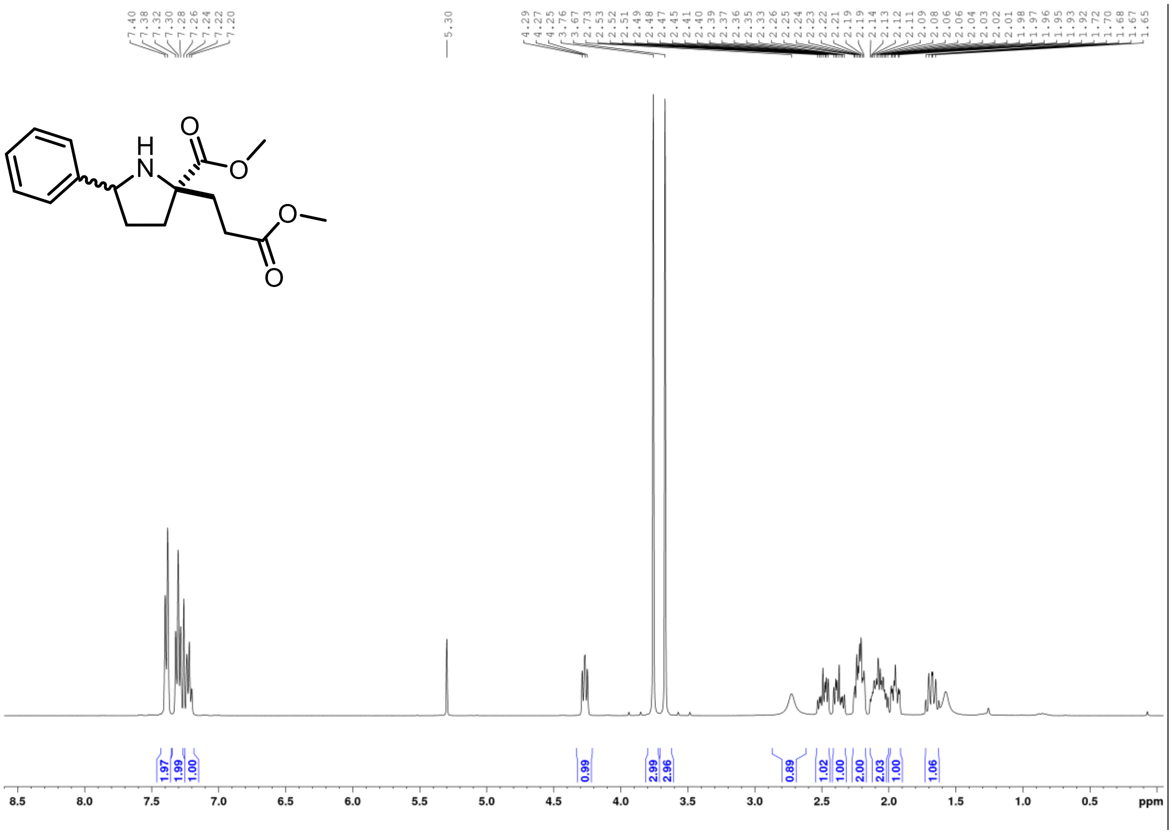

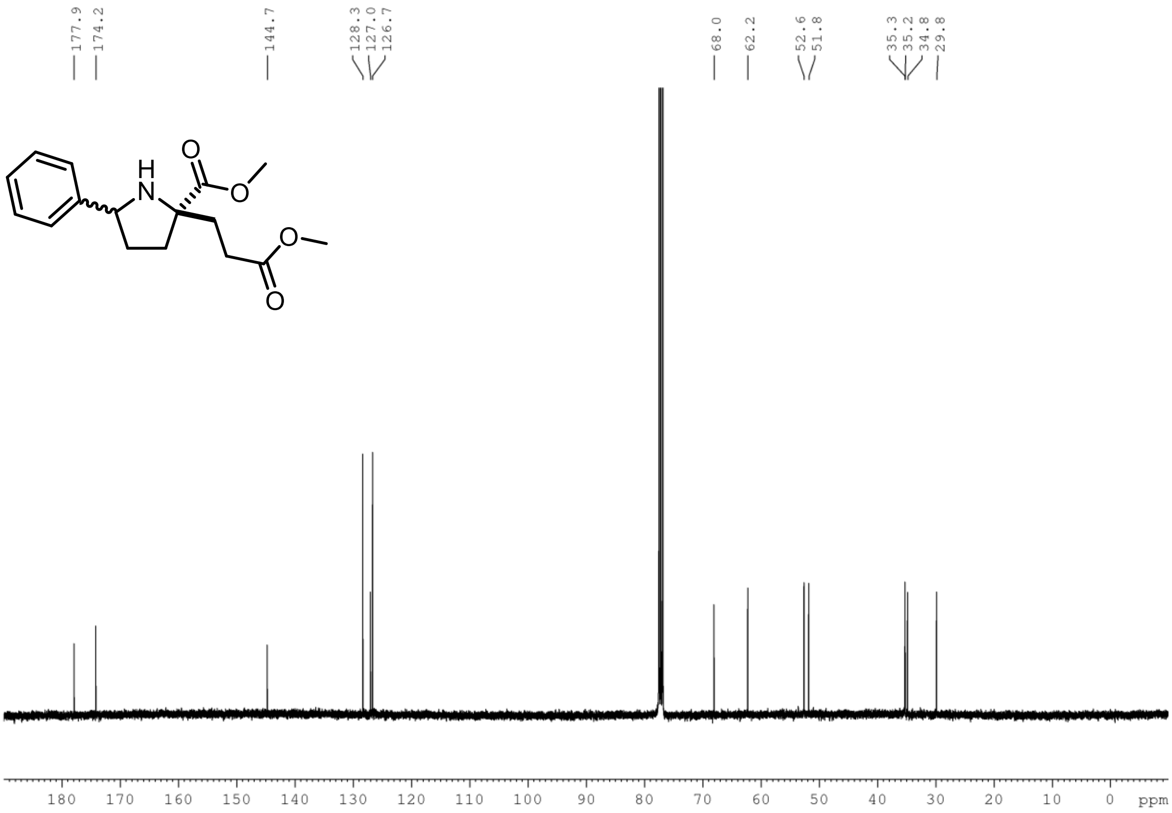

Methyl 2-(1-(*tert*-butoxycarbonyl)-3-(2-ethoxy-2-oxoethyl)azetidin-3-yl)-5-phenyl-3,4-dihydro-2H-pyrrole-2-carboxylate (24)

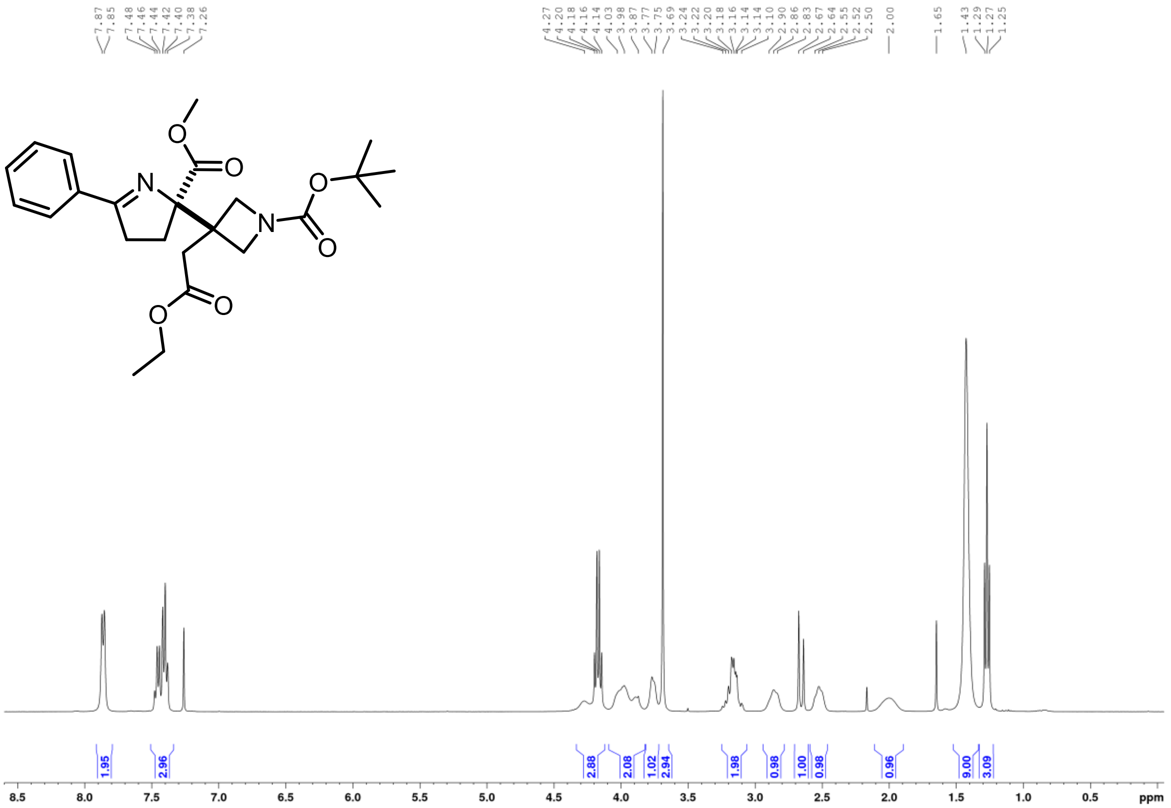

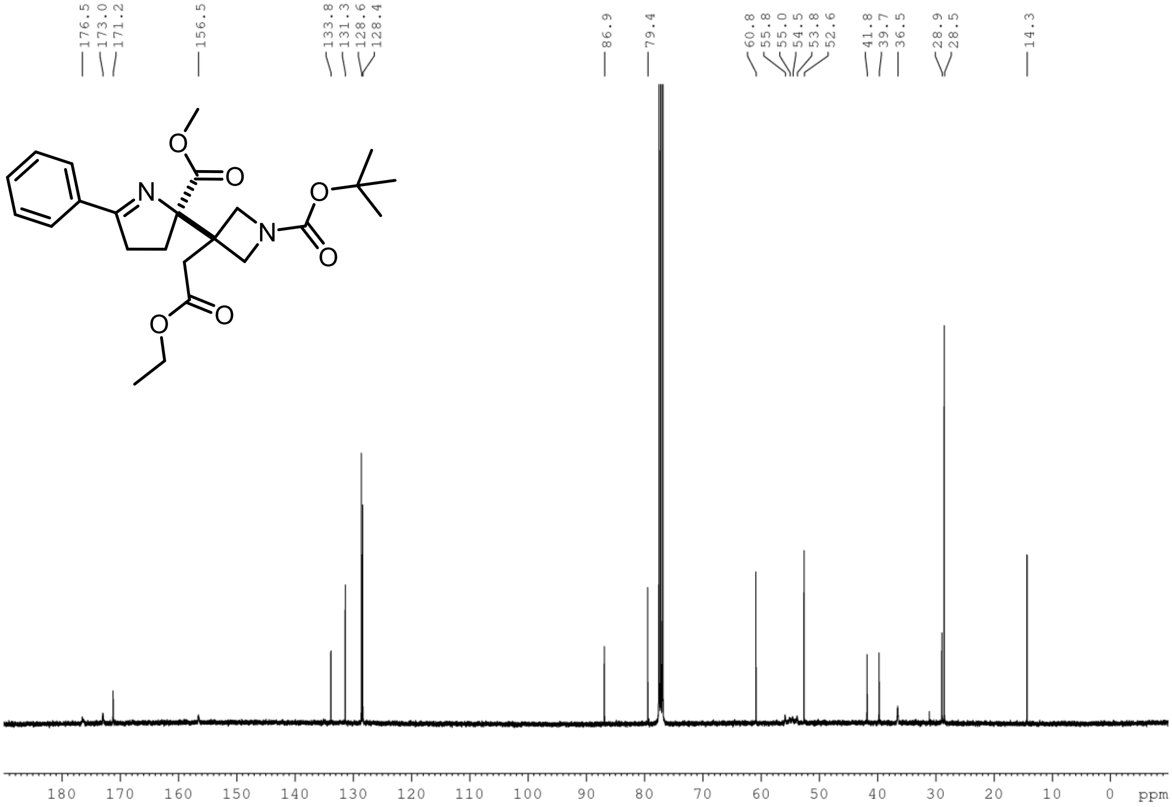

Methyl (2S)-6-(benzyloxy)-7-oxo-5-phenyl-1-azabicyclo[3.2.0]heptane-2-carboxylate (25)

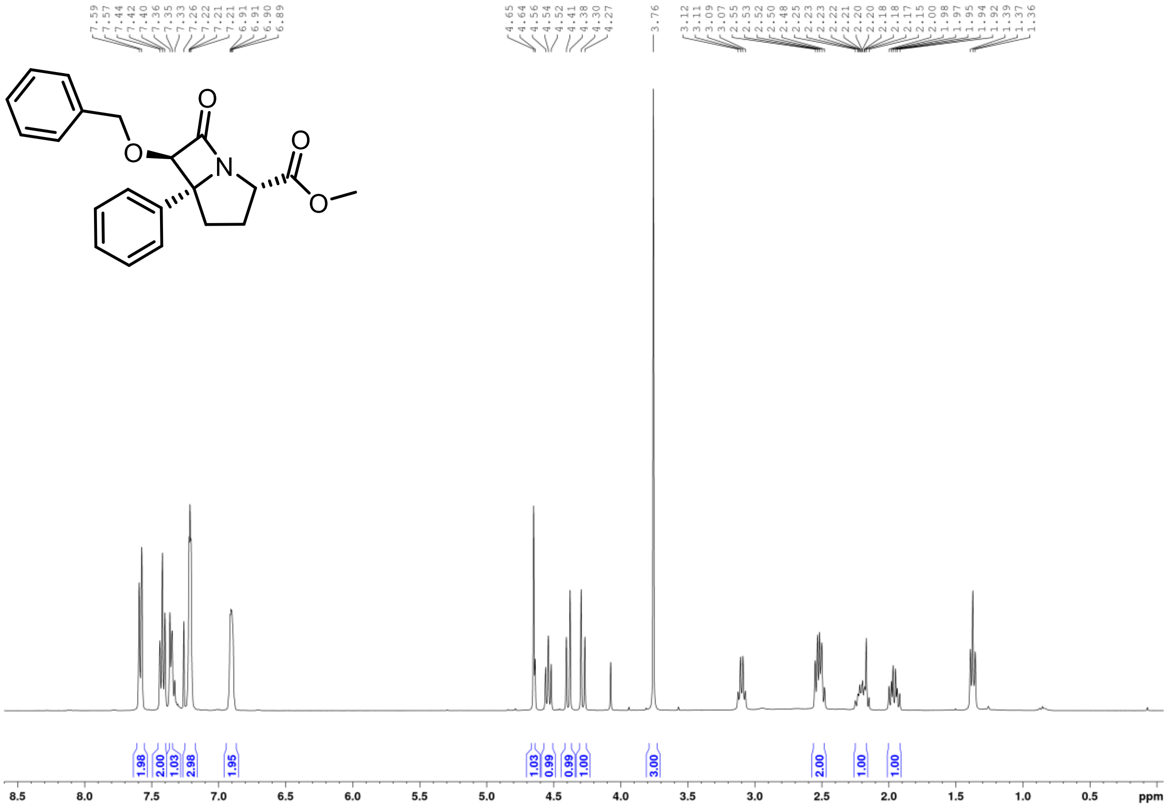

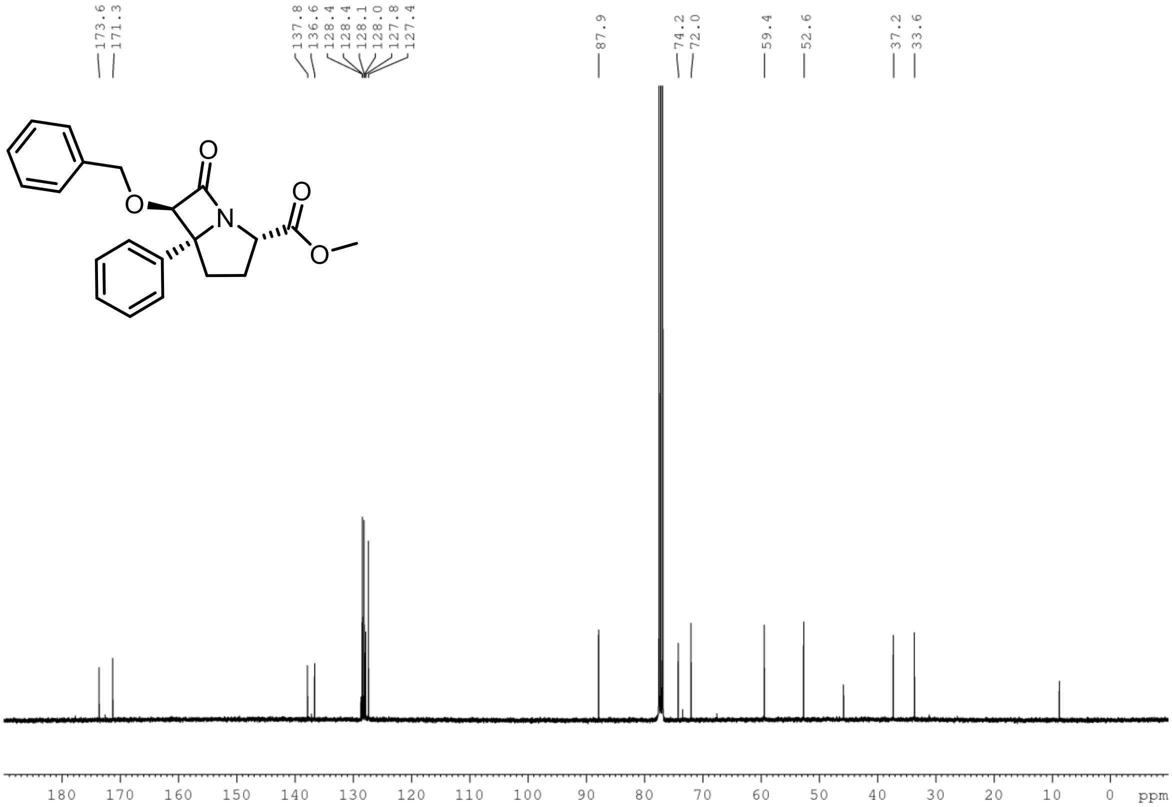

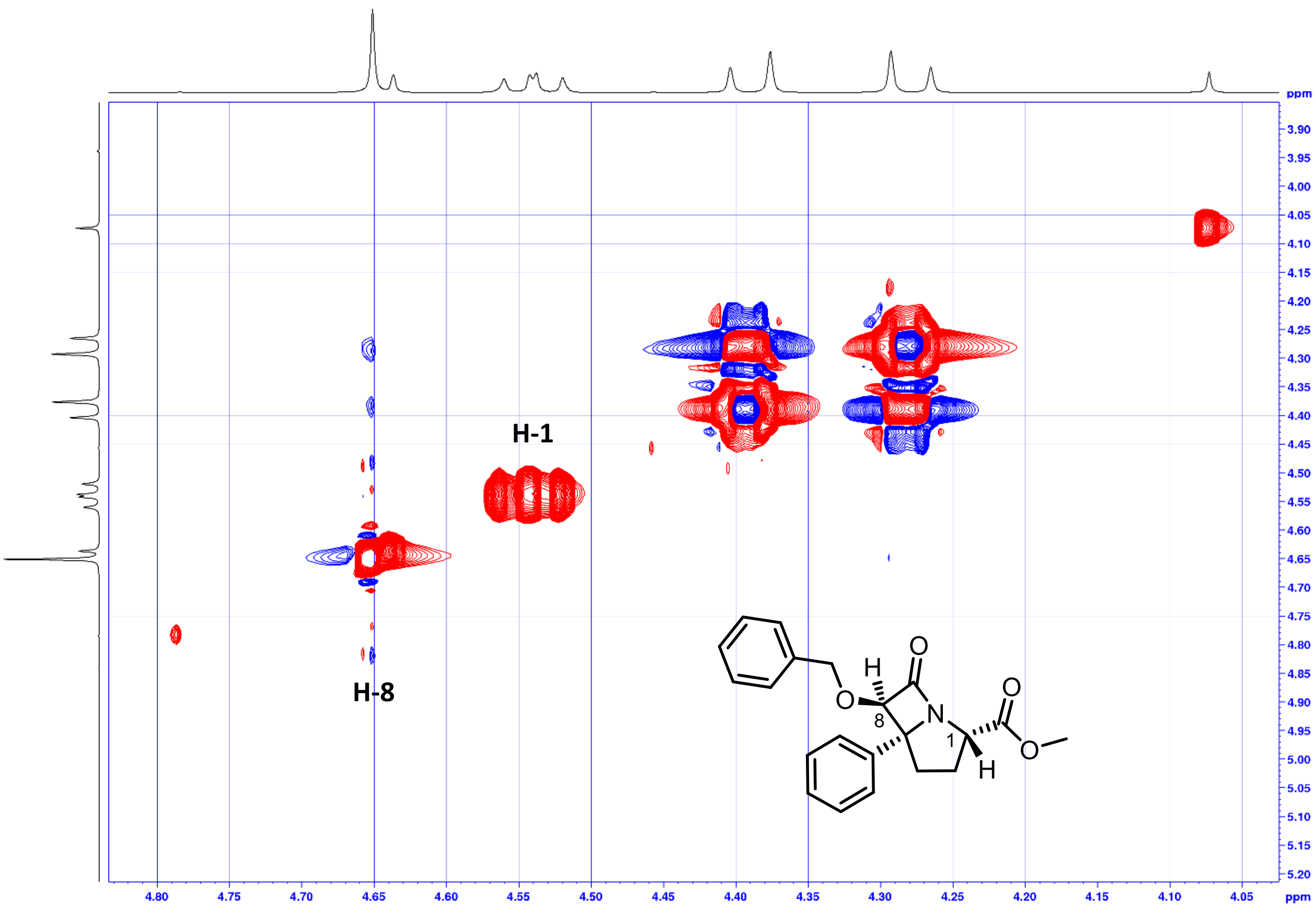

H-1
H-8
ppm

Crystallographic data

Methyl (3a*R*,6*S*,8a*S*,8b*S*)-2-methyl-1,3-dioxo-8a-phenyloctahydro-1*H*-dipyrrolo[1,2-*b*:3',4'-*d*]isoxazole-6-carboxylate (12)

CCDC deposition number	2184899	
Identification code	DS_B1_0031	
Empirical formula	$C_{17}H_{18}N_2O_5$	
Formula weight	330.33 $gmol^{-1}$	
Temperature	180(2) K	
Wavelength	1.54178 Å	
Crystal system	Monoclinic	
Space group	$P2_1$	
Unit cell dimensions	a = 6.4411(8) Å	α = 90°
	b = 30.630(4) Å	β = 101.461(6)°
	c = 8.0130(9) Å	γ = 90°
Volume	1549.3(3) $Å^3$	
Z	4	
Density (calculated)	1.416 gcm^{-3}	
Absorption coefficient	0.879 mm^{-1}	
F(000)	696	
Crystal size	0.220 × 0.020 × 0.020 mm^3	
Theta range for data collection	2.885–54.189°	
Index ranges	$-6 \le h \le 6$, $-30 \le k \le 31$, $-8 \le l \le 8$	
Reflections collected	8298	
Independent reflections	3615	
Completeness to theta = 54.189°	99.4%	
Absorption correction	Multi-scan	
Max. and min. transmission	0.7507 and 0.5557	
Refinement method	Full-matrix least-squares on F^2	
Data/restraints/parameters	3615/205/437	
Goodness-of-fit F^2	0.991	
Final R indices [I > 2σ(I)]	R1 = 0.0748, wR2 = 0.1326	
R indices (all data)	R1 = 0.1657, wR2 = 0.1673	

Largest diff. peak and hole 0.270 and –0.366 eÅ^{-3}

Flack parameter -0.1(3)

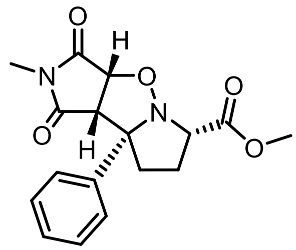

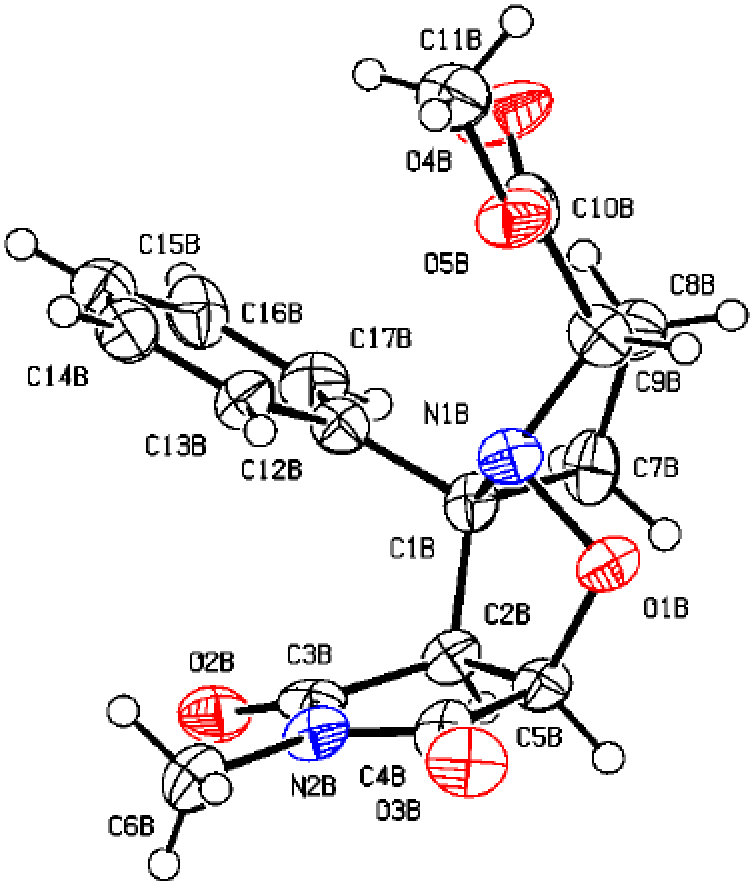

Computational Analysis

The Molecular Operating Environment (MOE) software package version 2012.10 from the Chemical Computing Group (CCG) was used to calculate physicochemical properties. For the calculations the Merck molecular force field 94X (MMFF94x), an all atom force field parameterised for small organic molecules with the Generalised Born solvation model, was applied to minimise the energy potential. Furthermore the LowModeMD search was selected for the conformation generation with detailed settings listed below.

Table S1. Conformational search settings

Rejection Limit	100
RMS Gradient	0.005
Iteration Limit	10000
MM Iteration Limit	500
RMSD Limit	0.15
Energy window	3
Conformation Limit	100

Compound collections

The physiochemical properties of the synthesised fragments were compared to two compound collections; the Maybridge Ro3 diversity set 1 and the Enamine sp3 rich fragments.

Principal moment of inertia

Principal moment of inertia (PMI) of the lowest energy conformation was calculated through the use of MOE. When applicable, the Boc group was removed virtually and indicated by "÷ Boc".

Tabel S2. Normalised PMI ratios

Compound	SMILES	npr1 (x-value)	npr2 (y-value)
**4**	O=C(OC)[C@H]1N=C(c2ccccc2)CC1	0.1528	0.9586
**21**	O=C1[C@]2(N=C(c3ccccc3)CC2)CCCN1	0.2255	0.9325
**22**	O=C1[C@@]2(C(c3ccccc3)CN1)N=C(c1ccccc1)CC2	0.2091	0.8767
**23b**	O=C(OC)CC[C@]1(C(=O)OC)N[C@H](c2ccccc2)CC1	0.4171	0.7732
**S18**	O=C(OC)[C@@]1(CCC#N)N=C(c2ccccc2)CC1	0.2068	0.8819
**S19**	O=[N+]([O-])CC(c1ccccc1)[C@]1(C(=O)OC)N=C(c2ccccc2)CC1	0.3632	0.9208
**S20**	O=C(OC)CC[C@]1(C(=O)OC)N=C(c2ccccc2)CC1	0.4286	0.7524
**23a**	O=C(OC)[C@@]12N([C@H](c3ccccc3)CC1)C(=O)CC2	0.3503	0.9053
**13**	O=C(OC)[C@H]1N2[C@](c3ccccc3)(C(C(=O)OC)=CO2)CC1	0.4258	0.7674

**25**	O=C(OC)[C@H]1N2C(=O)[C@H](OCc3ccccc3)[C@]2(c2ccccc2)CC1	0.4040	0.8483
**12**	O=C(OC)[C@H]1N2[C@](c3ccccc3)([C@@H]3C(=O)N(C)C(=O)[C@@H]3O2)CC1	0.5105	0.7627
**17**	O=C(OCC)Cn1nnc([C@H]2N[C@H](C(=O)OC)CC2)c1	0.1713	0.9015
**18**	O=C(OC)[C@H]1N[C@H](C#CC2=CCNCC2)CC1	0.1480	0.9250
**15**	O=C(OC)[C@H]1N2C(=O)C[C@@H]3C([C@@H]2CC1)=CC(=O)C3	0.2414	0.9004
**14**	O=C1N(C)CC(=O)N2[C@H](C#C)CC[C@@H]12	0.3619	0.7368
**16**	O=C(OC)[C@H]1N2C(=O)Cn3nncc3[C@@H]2CC1	0.2724	0.8546
**S14**	O=C(CC=C)N1[C@H](C(=O)OC)CC[C@H]1C#C	0.5083	0.6586
**S15**	O=C(OC(C)(C)C)N(CC(=O)N1[C@H](C(=O)OC)CC[C@H]1C#C)C	0.4736	0.8153
**S17**	O(CC=C)C[C@H]1N(CC=C)C(=O)CC1	0.3597	0.8899
**20**	O=C1N2[C@H](COCC=CC2)CC1	0.3697	0.7643
**S8-*anti***	O=[N+]([O-])C[C@@H](N1[C@H](C(=O)OC)CCC1=O)c1ccccc1	0.5525	0.7507
**10**	O=C1NC[C@H](c2ccccc2)N2C(=O)CC[C@@H]12	0.4547	0.7702
**S8-*syn***	O=[N+]([O-])C[C@H](N1[C@H](C(=O)OC)CCC1=O)c1ccccc1	0.4978	0.8375
**S6**	O=C(OC)[C@H]1N(CC#C)C(=O)CC1	0.5003	0.8729
**9**	O=C1N(C)C(C)=CN2C(=O)CC[C@@H]12	0.3480	0.6775
**8-enan.**	O=C1N2OCC=C[C@@H]2CN2C(=O)CC[C@@H]12	0.2894	0.7708
**S5**	O=C(NO)[C@H]1N(C/C=C/C=C)C(=O)CC1	0.4120	0.7417
**s4**	O=C(OC)[C@H]1N(C/C=C/C=C)C(=O)CC1	0.3943	0.8364
**S7**	O=C(NC)[C@H]1N(CC#C)C(=O)CC1	0.4927	0.8773
**S3**	O=C1N[C@@H]2[C@@H](c3ccccc3)CC=C[C@@H]2N2C(=O)CC[C@@H]12	0.3381	0.8940
**S2**	O=[N+]([O-])[C@H]1[C@H](N2[C@H](C(=O)OC)CCC2=O)C=CC[C@@H]1c1ccccc1	0.2853	0.9121
**S1**	O=C(OC)[C@H]1N([C@H]2[C@H](C#N)CCC=C2)C(=O)CC1	0.4331	0.8492
**6a**	O=C1NC[C@H]2[C@@H](N3C(=O)CC[C@@H]13)C=CCC2	0.4315	0.7490
**6b**	O=C1NC[C@H]2[C@@H](N3C(=O)CC[C@@H]13)CCCC2	0.4180	0.7762
**19**	O=C1C=CC2(OC[C@H]3N2C(=O)CC3)C=C1	0.3298	0.8403
**S16**	O(C)c1ccc(N2[C@H](CO)CCC2=O)cc1	0.2121	0.8846
**11**	O=[N+]([O-])c1cc(C#N)c(N2[C@H](C(=O)OC)CCC2=O)cc1	0.3221	0.7786
**S3**	O=C(OC)[C@H]1N([C@H]2[C@H](N)[C@@H](c3ccccc3)CC=C2)C(=O)CC1	0.2891	0.8855
**8-enant.**	O=C1N2OCC=C[C@H]2CN2C(=O)CC[C@@H]12	0.2868	0.7678

References

1) A. W. Hung, A. Ramek, Y. Wang, T. Kaya, J. A. Wilson, P. A. Clemons and D. W. Young, *Proc. Natl. Acad. Sci. USA*, 2011, **108**, 6799-6804.
2) S. Hübner, D. Michalik, H. Jiao, H. Neumann, S. Klaus, D. Strübing, A. Spannenberg and M. Beller, *Chem. Asian J.*, 2007, **2**, 734-746.
3) A. K. Weber, J. Schachtner, R. Fichtler, T. M. Leermann, J. M. Neudörfl and A. Jacobi von Wangelin, *Org. Biomol. Chem.*, 2014, **12**, 5267-5277.
4) N. Kato, E. Comer, T. Sakata-Kato, A. Sharma, M. Sharma, M. Maetani, J. Bastien, N. M. Brancucci, J. A. Bittker, V. Corey, D. Clarke, E. R. Derbyshire, G. L. Dornan, S. Duffy, S. Eckley, M. A. Itoe, K. M. J. Koolen, T. A. Lewis, P. S. Lui, A. K. Lukens, E. Lund, S. March, E. Meibalan, B. C. Meier, J. A. McPhail, B. Mitasev, E. L. Moss, M. Sayes, Y. V. Gessel, M. J. Wawer, T. Yoshinaga, A.-M. Zeeman, V. M. Avery, S. N. Bhatia, J. E. Burke, F. Catteruccia, J. C. Clardy, P. A. Clemons, K. J. Dechering, J. R. Duvall, M. A. Foley, F. Gusovsky, C. H. M. Kocken, M. Marti, M. L. Morningstar, B. Munoz, D. E. Neafsey, A. Sharma, E. A. Winzeler, D.F. Wirth, C. A. Scherer and S. L. Schreiber, *Nature,* 2016, **538**, 344–349
5) I. Bosque, E. Bagdatli, F. Foubelo and J. C. Gonzalez-Gomez, *J. Org. Chem.*, 2014, **79**, 1796-1804. Modified evaporation procedure on rotary evaporator: <10 °C at around 200 mbar following by 500 mbar at rt to heat up the round-bottomed flask and left at vacuum for 5 min. Before use the crude reagent was stirred in a minimum of DMF with molecular sieves.
6) T. Hoshikawa, S. Kamijo and M. Inoue, *Org. Biomol. Chem.*, 2013, **11**, 164-169.
7) Z. Yang, M. Zhong, X. Ma, S. De, C. Anusha, P. Parameswaran and H. W. Roesky, *Angew. Chem. Int. Ed.*, 2016, **55**, 669–673.
8) Grignard reagent was prepared according to a modified literature procedure by Cheung et al.(*Org. Lett.*, 2014, **16**, 2566–2569) To a solution of ethynyltrimethylsilane (7.1 mL, 50.9 mmol, 1.0 equiv.) in THF (28 mL) at 0 °C was slowly added EtMgBr (15.4 mL, 49.4 mmol, 3.2 M in THF, 0.97 equiv.) over 30 min under nitrogen atmosphere. The reaction mixture was stirred for 15 min at rt and then 1 h at 50 °C. The reaction mixture titrated from I_2 (1.15 M).
9) V. K. Aggarwal, C. J. Astle, H. Iding, B. Wirz, M. Rogers-Evans, *Tetrahedron. Lett.*, 2005, **46**, 945–947.
10) Z. Pei, X. Li, K. L. Longenecker, H. L. Sham, P. E. Wiedeman, WO Pat., 2005023762A1, 2005.
11) P. Capurro, L. Moni, A. Galatini, C. Mang and A. Basso, *Molecules*, **2018**, 23, 2758.
12) A. Turočkin, W. Raven and P. Selig, *Eur. J. Org. Chem.*, 2017, **2017**, 296–305.
13) T. A. King, H. L. Stewart, K. T. Mortensen, A. J. P. North, H. F. Sore and D. R. Spring, *Eur. J. Org. Chem.*, 2019, 5219–5229.
